# A Mobile Phone–Based Intervention to Reduce Mental Health Problems in Health Care Workers During the COVID-19 Pandemic (PsyCovidApp): Randomized Controlled Trial

**DOI:** 10.2196/27039

**Published:** 2021-05-18

**Authors:** Maria Antònia Fiol-DeRoque, Maria Jesús Serrano-Ripoll, Rafael Jiménez, Rocío Zamanillo-Campos, Aina María Yáñez-Juan, Miquel Bennasar-Veny, Alfonso Leiva, Elena Gervilla, M Esther García-Buades, Mauro García-Toro, Pablo Alonso-Coello, Guadalupe Pastor-Moreno, Isabel Ruiz-Pérez, Carolina Sitges, Javier García-Campayo, Joan Llobera-Cánaves, Ignacio Ricci-Cabello

**Affiliations:** 1 Health Research Institute of the Balearic Islands Palma de Mallorca Spain; 2 Primary Care Research Unit of Mallorca Balearic Islands Health Services Palma de Mallorca Spain; 3 Department of Psychology University of the Balearic Islands (UIB) Palma de Mallorca Spain; 4 Department of Health Valencian International University (VIU) Valencia Spain; 5 Research Group on Global Health & Human Development University of the Balearic Islands Palma de Mallorca Spain; 6 Department of Nursing and Physiotherapy University of the Balearic Islands Palma de Mallorca Spain; 7 Primary Care Prevention and Health Promotion Research Network Madrid Spain; 8 CIBER de Epidemiología y Salud Pública Madrid Spain; 9 Department of Clinical Epidemiology and Public Health Biomedical Research Institute Sant Pau Iberoamerican Cochrane Centre Barcelona Spain; 10 Andalusian School of Public Health Granada Spain; 11 Department of Psychology Research Institute of Health Sciences Palma de Mallorca Spain; 12 Aragon Institute for Health Research Miguel Servet University Hospital Zaragoza Spain

**Keywords:** COVID-19, randomized controlled trial, mental health, health care workers, mHealth, app

## Abstract

**Background:**

The global health emergency generated by the COVID-19 pandemic is posing an unprecedented challenge to health care workers, who are facing heavy workloads under psychologically difficult situations. Mental mobile Health (mHealth) interventions are now being widely deployed due to their attractive implementation features, despite the lack of evidence about their efficacy in this specific population and context.

**Objective:**

The aim of this trial is to evaluate the effectiveness of a psychoeducational, mindfulness-based mHealth intervention to reduce mental health problems in health care workers during the COVID-19 pandemic.

**Methods:**

We conducted a blinded, parallel-group, controlled trial in Spain. Health care workers providing face-to-face health care to patients with COVID-19 were randomly assigned (1:1) to receive the PsyCovidApp intervention (an app targeting emotional skills, healthy lifestyle behavior, burnout, and social support) or a control app (general recommendations about mental health care) for 2 weeks. The participants were blinded to their group allocation. Data were collected telephonically at baseline and after 2 weeks by trained health psychologists. The primary outcome was a composite of depression, anxiety, and stress (overall score on the Depression Anxiety Stress Scale-21 [DASS-21]). Secondary outcomes were insomnia (Insomnia Severity Index), burnout (Maslach Burnout Inventory Human Services Survey), posttraumatic stress (Davidson Trauma Scale), self-efficacy (General Self-Efficacy Scale), and DASS-21 individual scale scores. Differences between groups were analyzed using general linear modeling according to an intention-to-treat protocol. Additionally, we measured the usability of the PsyCovidApp (System Usability Scale). The outcome data collectors and trial statisticians were unaware of the treatment allocation.

**Results:**

Between May 14 and July 25, 2020, 482 health care workers were recruited and randomly assigned to PsyCovidApp (n=248) or the control app (n=234). At 2 weeks, complete outcome data were available for 436/482 participants (90.5%). No significant differences were observed between the groups at 2 weeks in the primary outcome (standardized mean difference –0.04; 95% CI –0.11 to 0.04; *P*=.15) or in the other outcomes. In our prespecified subgroup analyses, we observed significant improvements among health care workers consuming psychotropic medications (n=79) in the primary outcome (–0.29; 95% CI –0.48 to –0.09; *P*=.004), and in posttraumatic stress, insomnia, anxiety, and stress. Similarly, among health care workers receiving psychotherapy (n=43), we observed improvements in the primary outcome (–0.25; 95% CI –0.49 to –0.02; *P*=.02), and in insomnia, anxiety, and stress. The mean usability score of PsyCovidApp was high (87.21/100, SD 12.65). After the trial, 208/221 participants in the intervention group (94.1%) asked to regain access to PsyCovidApp, indicating high acceptability.

**Conclusions:**

In health care workers assisting patients with COVID-19 in Spain, PsyCovidApp, compared with a control app, reduced mental health problems at 2 weeks only among health care workers receiving psychotherapy or psychotropic medications.

**Trial Registration:**

ClinicalTrials.gov NCT04393818; https://clinicaltrials.gov/ct2/show/NCT04393818.

## Introduction

Since the declaration of the COVID-19 pandemic, the disease has spread globally, with almost 55 million known cases and a death toll of over 1.3 million people [1]. Early anecdotal evidence from Wuhan showed how this unprecedented situation impacted the mental health of frontline health care workers [2]. This was confirmed by subsequent systematic reviews [3,4], in which a remarkably high prevalence of acute stress (40%), anxiety (30%), burnout (28%), depression (24%), and posttraumatic stress disorder (13%) was observed among frontline health care workers. Working in an environment with a high risk of infection, job-related stress, heavy workloads, and lack of protective equipment were found to significantly exacerbate these psychological problems [3]. On May 8, 2020, Spain reported the highest cumulative number of COVID-19 infections among health care workers worldwide (30,663 infections, accounting for 20% of all infections in health care workers worldwide) [5]. Not surprisingly, the psychological consequences disproportionally affected the mental health of Spanish health care workers, with approximately 57% of them presenting symptoms of posttraumatic stress disorder, 59% presenting symptoms of anxiety disorder, 46% presenting symptoms of depressive disorder, and 41% feeling emotionally drained [6].

Health services worldwide are being urged to implement strategies to mitigate the severe psychological consequences experienced by health care workers. Among the different types of strategies considered, mobile health (mHealth) interventions are receiving special attention [7] not only because of their attractive implementation features but also because they can be delivered in the absence of face-to-face interactions, reducing the risk of infection with SARS-CoV-2. Further, they can address non–treatment-seeking behavior (a common issue among health care workers [8]), as they provide the opportunity to engage individuals in need of treatment in a timely and anonymous fashion. This growing interest in mHealth interventions is supported by the positive results of acceptance rates [9] and sustainability [10] observed in other contexts and populations. Recent trials have examined the efficacy of mHealth interventions addressing mental health problems, including depression [11,12], suicide [13], schizophrenia [14], substance use disorders [15], and psychosis [16], among others [17]. Recent systematic reviews investigating the efficacy of smartphone apps for mental health show that these apps can produce significant reductions in anxiety [18] and depression [19]. However, the effectiveness of mHealth interventions in health care workers in the COVID-19 pandemic context is largely unknown, as observed by recent reviews that highlighted the lack of evaluations of client-relevant outcomes [20] and the lack of both quantitative and qualitative evidence to inform the selection of interventions that are beneficial to the mental health of frontline health care workers [21]. Hence, robust, large-scale trials are urgently needed to determine the extent to which mHealth interventions can improve the mental health of frontline health care workers.

This blinded, individually randomized, parallel-group, controlled trial aimed to evaluate the effectiveness of PsyCovidApp (a self-managed and self-guided psychoeducational mobile-based intervention with no therapist support) to reduce symptoms of depression, anxiety, stress and other mental health problems in health care workers during the COVID-19 pandemic in Spain.

## Methods

### Design and Setting

We conducted a blinded, individually randomized, parallel-group, controlled trial in Spain. Because the ultimate goal of the study was to inform decisions about rolling up the intervention to make it available to all health care workers in Spain, we used a pragmatic approach. Pragmatic trials are ideally suited to inform choices between treatments because as opposed to exploratory trials (which typically examine treatment benefits under ideal conditions using carefully defined subjects), pragmatic trials enable measurement of the effectiveness of interventions under real conditions in a sample of participants to whom the treatment would be applied [22]. Participants were individually randomized with an allocation ratio of 1:1 to receive either the PsyCovidApp intervention or the control app over 2 weeks. Ethical approval was obtained by the Research Ethics Committee of the Balearic Islands (IB 4216/20 PI). The study protocol and statistical analysis plan have been published previously (ClinicalTrials.gov NCT04393818) [23].

### Participants

The target population was male and female health care workers aged >18 years who had provided health care to patients with COVID-19 during the viral outbreak in Spain. We included health care workers from any medical specialty (pneumology, internal medicine, emergency, primary care, etc) and role (physicians, nurses, nurse assistants, etc) with access to a smartphone. We included health care workers who had provided direct, face-to-face health care to patients with a diagnosis of infection with COVID-19. We excluded health care workers who were not able to download and activate the app used to deliver the intervention during the next 10 days following the baseline assessment.

Following a purposive sampling method, we sent invitations to health care workers to participate in the trial through direct contact via email and telephone to key stakeholders (115 hospital and home care centers, 138 professional associations, and 8 scientific societies and trade unions) and by social media. Health care workers who were willing to participate registered their interest by completing a web-based questionnaire, consenting to be contacted telephonically. A team of 23 health psychologists who had previously received a 2-hour training session (to ensure homogeneity in recruitment, questionnaire administration, and data entry methods) contacted the registered health care workers by telephone to confirm the eligibility criteria and obtain informed consent (audio-recorded). The recruitment period spanned 10 weeks, from May 14 to July 25, 2020. Participants were enrolled on a rolling “first-come-first-served” basis until target sample sizes were met. To incentivize trial participation, we offered participation certificates to all health care workers completing the postintervention assessment.

### Randomization and Masking

Participants were randomly assigned (1:1) to receive the PsyCovidApp intervention or the control app over 2 weeks by a designated researcher (MAF, who was not involved in data collection or analysis) using a computer-generated sequence of random numbers created by internet relay chat (IRC). Randomization was not stratified. Health care workers were blinded to group allocation (as both groups received an app). The outcome data collectors and trial statisticians were unaware of the treatment allocation.

### Procedures

Immediately after obtaining informed consent, a team of psychologists conducted a psychological (preintervention) evaluation via telephone interview and instructed participants on how to download the Clinicovery App (Apploading, Inc). Clinicovery is the app that was used to deliver either the contents of the PsyCovidApp intervention or the control contents. Within 48 hours after participants successfully downloaded and activated the app (user activation was used as a checkpoint to ensure the participants could successfully access the app), a member of our research team loaded the contents to the app according to the group the participants had been allocated to. During the following 14 days, all health care workers had access to the content of their assigned group (PsyCovidApp intervention or control). The PsyCovidApp intervention was developed by a group of psychologists (MJSR, EG, CS, RJ, MEGB), psychiatrists (JGC, MGT), and experts in healthy lifestyle promotion (AMYJ, MBV), informed by findings from an exploratory qualitative study involving in-depth interviews with 9 health care workers seeking psychological support as a result of their professional activity during the COVID-19 pandemic (unpublished results). PsyCovidApp was specifically designed to prevent and mitigate the most frequent mental problems suffered by health care workers who are dealing with the COVID-19 emergency (depression, anxiety, posttraumatic stress, and burnout). A detailed description of the intervention is available elsewhere [23]. In short, the self-managed psychoeducational intervention, based on cognitive-behavioral therapy and mindfulness approaches, included written and audiovisual content targeting four areas: emotional skills, healthy lifestyle behavior, work stress and burnout, and social support. Additionally, the intervention included daily prompts (notifications) that included brief questionnaires to monitor mental health status, followed by short messages offering tailored information and resources based on the participants´ responses. The full content of the intervention is available in Multimedia Appendix 1.

Participants in the Control App group had access through the Clinicovery app to brief written information about the mental health care of health care workers during the COVID-19 pandemic (adapted from a set of materials developed by the Spanish Society of Psychiatry; the contents are available in Multimedia Appendix 2).

After 2 weeks, the apps in both groups were disabled, and a postintervention psychological assessment was undertaken. The follow-up was undertaken via telephone between 24 hours and 10 days after the intervention concluded, and it included the same questionnaires used in the first evaluation and the System Usability Scale (SUS) [24]. Once the postintervention assessment had finished, all participants were offered free, unrestricted access to PsyCovidApp.

### Outcome Measures

The primary outcome was an overall index of depression, anxiety, and stress (overall score of the Spanish version of the Depression, Anxiety, and Stress Scale [DASS-21] instrument [25]) assessed at 2 weeks. The score ranges from 0 (best outcome) to 21 (worst outcome). The instrument contains three 7-item subscales assessing the presence and intensity of depression, anxiety, and stress. The items are based on a Likert scale ranging from 0 to 3 points. The instrument shows adequate internal consistency (Cronbach α=.91) and construct validity (3-factor structure identified after exploratory factor analysis, explaining 50% of the total variance) [26].

Secondary outcome measures were the difference between the intervention and control groups in the mean scores of the following instruments:

- Davidson Trauma Scale (DTS) [27]. The DTS is a 17-item self-report Likert scale instrument that assesses the 17 symptoms of posttraumatic stress disorder in the *Diagnostic and Statistical Manual of Mental Disorders, Fourth Edition*. Both a frequency and a severity score can be determined. The DTS yields a frequency score (ranging from 0 to 68), severity score (ranging from 0 to 68), and total score (ranging from 0 to 136). Higher scores are indicative of a worse outcome. The Spanish version of the instrument shows adequate reliability (test-retest intraclass correlation=0.87; α=.90) as well as high discriminant validity [28]. For this study, the instrument was adapted to measure posttraumatic stress disorder since the onset of the COVID-19 pandemic. This adaptation consisted of reformulating the stem of all the items, replacing “during the last week” with “since the onset of the current COVID-19 pandemic”.
- Maslach Burnout Inventory - Human Services Survey (MBI-HSS) [29], which yields specific scores for each of its three subscales (emotional exhaustion, α=.85; depersonalization, α=.58; and professional accomplishment, α=.71). The Spanish version of the instrument shows adequate internal consistency (except for the depersonalization subscale) and adequate factorial validity [30].
- Insomnia Severity Index (ISI) [31]. The ISI is a 7-item, self-reported Likert scale instrument assessing the severity of both nighttime and daytime components of insomnia. Scores range from 0 (best outcome) to 28 (worst outcome). In the Spanish version, principal component analysis showed only one factor explaining 69% of the total variance, with an internal consistency reliability of 0.91. Regarding its validity, the ISI shows statistically significant positive correlations with the Athens Insomnia Scale-5 (*r*=0.93) and negative correlations with the Mini Mental State Examination (*r*=–0.15) [32].
- General Self-Efficacy Scale (GSE) [33]. The GSE is a 10-item, Likert-scale, self-reported instrument that assesses optimistic self-beliefs to cope with a variety of difficult demands in life. Scores range from 10 (worst outcome) to 40 (best outcome). The Spanish version shows adequate internal consistency (α=.85) and construct validity [34].
- Individual subscales of the DASS-21 instrument: depression (α=.90), anxiety (α=.88), and stress (α=.88) [35,36].
- Usability of PsyCovidApp at postintervention: SUS [24]. Higher scores are indicative of higher usability, and scores above the 68-point threshold can be considered to indicate high usability. The Spanish version shows adequate internal consistency (α=.80) and concurrent validity (significant correlation with the Adjective Rating Scale; *r*=0.56) [37].

### Statistical Analysis

The sample size and power calculations have been described previously [23]. We estimated that 440 participants (220 per group, allowing for 10% attrition) would be required to detect at least an effect size of 0.25 (standardized between-group mean difference) on DASS-21 with 80% power and 5% α (one-sided).

The analyses followed the agreed statistical analysis plan, published before database lock [23]. Descriptive statistics summarizing prerandomization variables, and outcome measures at 2 weeks were reported by treatment group and overall. Differences between groups of primary and secondary outcomes were analyzed using general linear modelling (analysis of covariance) for continuous variables, adjusted by baseline score. We report standardized between-group differences in primary and secondary outcome measures at 2 weeks. In those outcomes that were interpreted as being in favor of PsyCovidApp if the standardized group difference was more than 0, the estimated effect was reflected (ie, multiplied by –1) so that outcomes for which the standardized group difference was lower than 0 could be homogenously interpreted as being in favor of the intervention. In the primary statistical analysis, all health care workers who agreed to participate were included in the analysis according to the group to which they were assigned. We used multiple imputation by chained equations to fill in missing values (50 imputation sets) [38]. In Multimedia Appendix 3, we report unstandardized between-group differences. In the analysis, *P* values and CIs were not corrected for multiple secondary outcome comparisons.

We conducted three prespecified subgroup analyses to examine the impact of the PsyCovidApp intervention on the primary and secondary outcomes based on the following baseline characteristics: use of psychotropic medications (yes vs no), use of psychotherapy (yes vs no), and symptomatology of depression, anxiety, and stress (yes vs no, based on baseline DASS-21 median overall score). We conducted statistical tests for interaction (including an interaction term in the models) to determine whether chance was an unlikely explanation for the apparent subgroup effects identified. We used the Instrument to Assess the Credibility of Effect Modification Analyses (ICEMAN) [39] to assess the credibility of our subgroup analyses. As a sensitivity analysis, we reanalyzed all outcomes on a complete case basis (ie, without imputation or adjustment for baseline predictors of missingness). We used SPSS, version 25 (IBM Corporation) and Stata, version 13 (StataCorp LLC) to conduct the statistical analyses.

### Role of the Funding Source

The funder of the study had no role in study design, data collection, data analysis, data interpretation, or writing of the report. All authors had full access to all the data in the study and had final responsibility for the decision to submit for publication.

## Results

**Figure 1:**
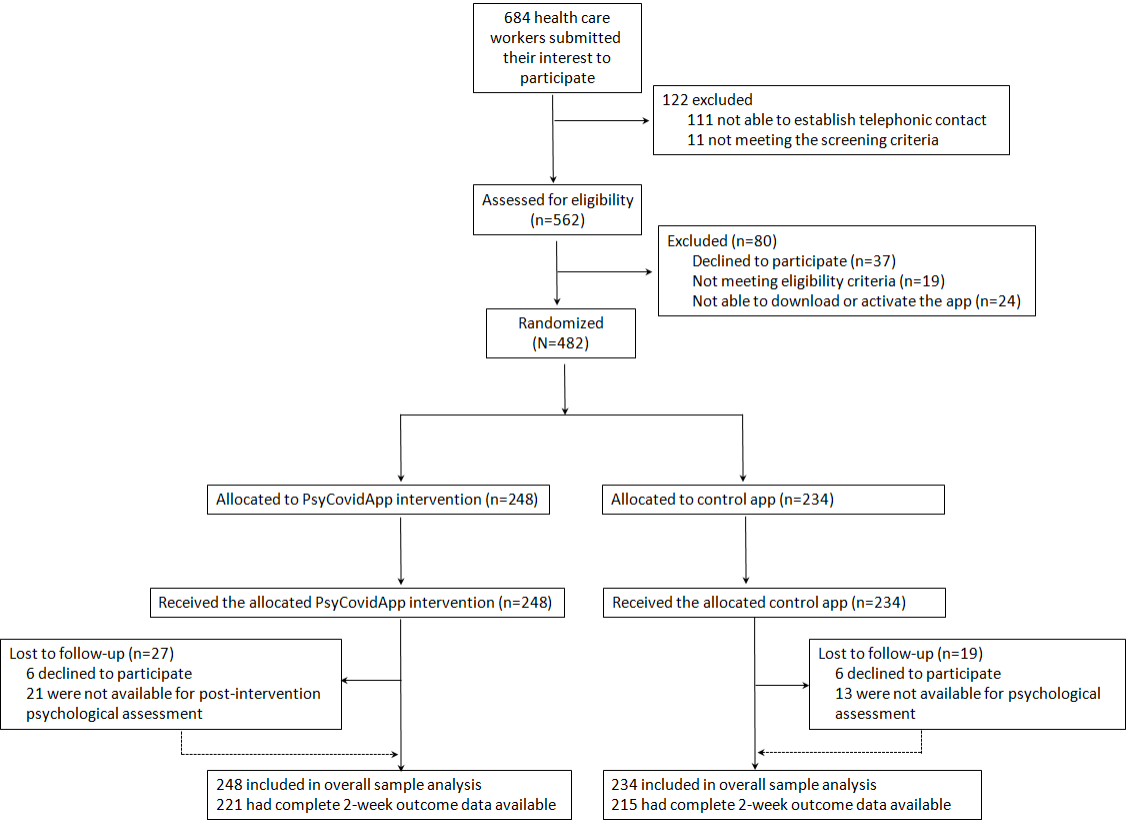
Trial profile. Multiple imputation was used to facilitate the overall sample analysis; all randomized participants contributed to the statistical analysis.

Between May 14 and July 25, 2020, 684 health care workers submitted an expression of interest in enrolling in the PsyCovidApp trial. 482 eligible participants provided informed consent and were randomly assigned to the PsyCovidApp intervention group (n=248) or the Control App group (n=234; Figure 1). Recruitment by region is shown in Multimedia Appendix 4. Participants included health care workers from all regions in Spain except the Canary Islands and Cantabria.

The baseline characteristics were balanced between the groups (Table 1). Most participants were women (401/482; 83.2%), and the median age was 42 years (IQR 33-49). Approximately one-third (161, 33.4%) of the 482 participants were nurses, 153 (31.7%) were physicians, and 147 (30.5%) were nurse assistants. Most worked in the hospital setting: 98/482 (20.3%) in internal medicine, 81/482 (16.8%) in intensive care units, 79/482 (16.4%) in hospital emergency units, 31/482 (6.4%) in infection units, and 103/482 (21.4%) in other hospital units. Of the 482 participants, 61 (12.7%) worked in primary care and 29 (6%) in home-care settings.

**Table 1:** Baseline demographic characteristics of the intention-to-treat population (N=482).

Characteristic		PsyCovidApp group (n=248)	Control App group (n=234)	Total (N=482)
**Age, years**				
	Mean (SD)	42.07 (11.0)	40.62 (9.6)	41.37 (10.4)
	Median (IQR; range)	42 (34-51; 23-63)	41 (32-47; 23-61)	41.5 (33-49; 23-63)
	<36, n (%)	75 (30.2)	82 (35)	157 (32.6)
	36-45, n (%)	79 (31.9)	81 (34.6)	160 (33.2)
	46-55, n (%)	60 (24.2)	52 (22.2)	112 (23.2)
	>55, n (%)	34 (13.7)	19 (8.1)	53 (11)
**Gender, n (%)**				
	Male	38 (15.3)	43 (18.4)	81 (16.8)
	Female	210 (84.7)	191 (81.6)	401 (83.2)
**Occupational role, n (%)**				
	Physician	76 (30.6)	77 (32.9)	153 (31.7)
	Nurse	87 (35.1)	74 (31.6)	161 (33.4)
	Nurse assistant	77 (31)	70 (29.9)	147 (30.5)
	Other	8 (3.2)	13 (5.6)	22 (4.6)
**Setting, n (%)**				
	Primary care	35 (14.1)	26 (11.1)	61 (12.7)
	Internal medicine	48 (19.4)	50 (21.4)	98 (20.3)
	Intensive care unit	40 (16.1)	41 (17.5)	81 (16.8)
	Hospital emergencies unit	31 (12.5)	48 (20.5)	79 (16.4)
	Home care	19 (7.7)	10 (4.3)	29 (6)
	Infection unit	16 (6.5)	15 (6.4)	31 (6.4)
	Other hospital unit	59 (23.8)	44 (18.8)	103 (21.4)
**Time working with patients with COVID-19 (weeks), n (%)**				
	<2	9 (3.6)	4 (1.7)	13 (2.7)
	2-4	20 (8.1)	14 (6)	34 (7.1)
	>4	219 (88.3)	216 (92.3)	435 (90.2)
**Infected with COVID-19, n (%)**				
	Yes	31 (12.5)	34 (14.5)	65 (13.5)
	No	214 (86.3)	195 (83.3)	409 (84.9)
	Unknown	3 (1.2)	5 (2.1)	8 (1.7)
**Perception about the adequacy of available measures to protect health care workers from COVID-19, n (%)**				
	Inadequate measures	84 (33.9)	77 (32.9)	161 (33.4)
	Adequate measures	163 (65.7)	157 (67.1)	320 (66.4)
**Perception about the information about the procedures to provide health care to patients with COVID-19, n (%)**				
	Inadequate information	101 (40.7)	116 (49.6)	217 (45)
	Adequate information	147 (59.3)	117 (50)	264 (54.8)
**Currently consuming psychotropic medication, n (%)**				
	No	207 (83.5)	196 (83.8)	403 (83.6)
	Yes	41 (16.5)	38 (16.2)	79 (16.4)
**Currently receiving psychotherapy, n (%)**				
	No	227 (91.5)	212 (90.6)	439 (91.1)
	Yes	21 (8.5)	22 (9.4)	43 (8.9)

By the time of recruitment, most participants (435/482, 90.2%) had been providing health care to patients with COVID-19 for more than 4 weeks, and 65/482 (13.5%) had received a diagnosis of COVID-19 infection. Approximately one-third (161/482, 33.4%) perceived that the measures offered to protect them from COVID-19 had been inadequate, and 217/482 (45%) perceived that they had received inadequate information about the procedures to provide health care to patients with COVID-19. Of the 482 participants, 79 (16.4%) were using psychotropic medications, and 43 (8.9%) were receiving psychotherapy.

In relation to their mental health, 206 of the 482 participants (42.7%) presented symptoms of depression, 250 (51.9%) had symptoms of anxiety, 292 (60.6%) had symptoms of stress, 194 (40.2%) had symptoms of posttraumatic stress, and 128 (26.6%) had symptoms of insomnia (Table 2). Concerning burnout, 282/482 participants (58.5%) presented emotional exhaustion, 165/482 (34.2%) presented emotional depersonalization, and 203/482 (42.1%) presented moderate or low professional accomplishment. The mean self-efficacy score was 32.2 out of 40 (SD 4.7), indicating a high level of self-efficacy.

**Table 2:** Baseline clinical characteristics of the intention-to-treat population (N=482).

Characteristic			PsyCovidApp group (n=248)	Control App group (n=234)	Total (N=482)
**Depression, anxiety, and stress**					
	DASS-21^a^ overall score, mean (SD)		5.8 (3.9)	6.1 (3.8)	6.0 (3.8)
	**Depression (DASS-21 subscale), n (%)**				
		No symptoms (<5 points)	143 (57.7)	133 (56.8)	276 (57.3)
		Mild (5-6 points)	26 (10.5)	32 (13.7)	58 (12)
		Moderate (7-10 points)	55 (22.2)	48 (20.5)	103 (21.4)
		Severe (11-13 points)	15 (6)	11 (4.7)	26 (5.4)
		Extremely severe (>13 points)	9 (3.6)	10 (4.3)	19 (3.9)
	**Anxiety (DASS-21 subscale), n (%)**				
		No symptoms (<4 points)	121 (48.8)	111 (47.4)	232 (48.1)
		Mild (4 points)	32 (12.9)	25 (10.7)	57 (11.8)
		Moderate (5-7 points)	40 (16.1)	42 (17.9)	82 (17)
		Severe (8-9 points)	24 (9.7)	20 (8.5)	44 (9.1)
		Extremely severe (>9 points)	31 (12.5)	36 (15.4)	67 (13.9)
	**Stress (DASS-21 subscale), n (%)**				
		No symptoms (<8 points)	104 (41.9)	86 (36.8)	190 (39.4)
		Mild (8-9 points)	28 (11.3)	32 (13.7)	60 (12.4)
		Moderate (10-12 points)	56 (22.6)	58 (24.8)	114 (23.7)
		Severe (13-16 points)	43 (17.3)	45 (19.2)	88 (18.3)
		Extremely severe (>16 points)	17 (6.9)	13 (5.6)	30 (6.2)
**Posttraumatic stress (DTS^b^), n (%)**					
	No (<40 points)		150 (60.5)	138 (59)	288 (59.8)
	Yes (≥40 points)		98 (39.5)	96 (41)	194 (40.2)
**Burnout (MBI-HSS^c^), n (%)**					
	**Emotional exhaustion**				
		Low (0-16 points)	95 (38.3)	89 (38)	184 (38.2)
		Moderate (17-26 points)	61 (24.6)	50 (21.4)	111 (23)
		High (>27 points)	92 (37.1)	95 (40.6)	187 (38.8)
	**Professional accomplishment subscale**				
		High (>39 points)	144 (58.1)	135 (57.8)	279 (57.9)
		Moderate (32-38 points)	65 (26.2)	54 (23.1)	119 (24.7)
		Low (0-31 points)	39 (15.7)	45 (19.2)	84 (17.4)
	**Depersonalization subscale**				
		Low (0-6 points)	163 (65.7)	154 (65.8)	317 (65.8)
		Moderate (17-12 points)	50 (20.2)	31 (13.2)	81 (16.8)
		High (>13 points)	35 (14.1)	49 (20.9)	84 (17.4)
**Insomnia (ISI^d^), n (%)**					
	Not clinically significant (0-7 points)		102 (41.1)	87 (37.2)	189 (39.2)
	Subthreshold insomnia (8-14 points)		89 (35.9)	76 (32.5)	165 (34.2)
	Clinical insomnia (moderate severity) (15-21 points)		49 (19.8)	61 (26.1)	110 (22.8)
	Clinical insomnia (severe) (22–28)		8 (3.2)	10 (4.3)	18 (3.7)
**Self-efficacy (GSE^e^)**					
	Mean score out of 40 points (SD)		32.4 (4.7)	32 (4.7)	32 (4.7)

^a^DASS-21: Depression, Anxiety, and Stress Scale-21.

^b^DTS, Davidson Trauma Scale.

^c^MBI-HSS: Maslach Burnout Inventory - Human Services Survey.

^d^ISI: Insomnia Severity Index.

^e^GSE: General Self-Efficacy Scale.

At 2 weeks, 27 of the 248 participants (10.9%) in the PsyCovidApp group and 19 of the 234 participants (8.1%) in the Control App group were lost to follow-up because they decided to withdraw from the study at the time of the postintervention psychological assessment (6 in the intervention group and 6 in the control group) or because we were unable to reach them for the telephonic postintervention psychological assessment (21 in the intervention group and 13 in the control group). None of the participants were deemed to be associated with reported adverse events or death.

**Figure 2:**
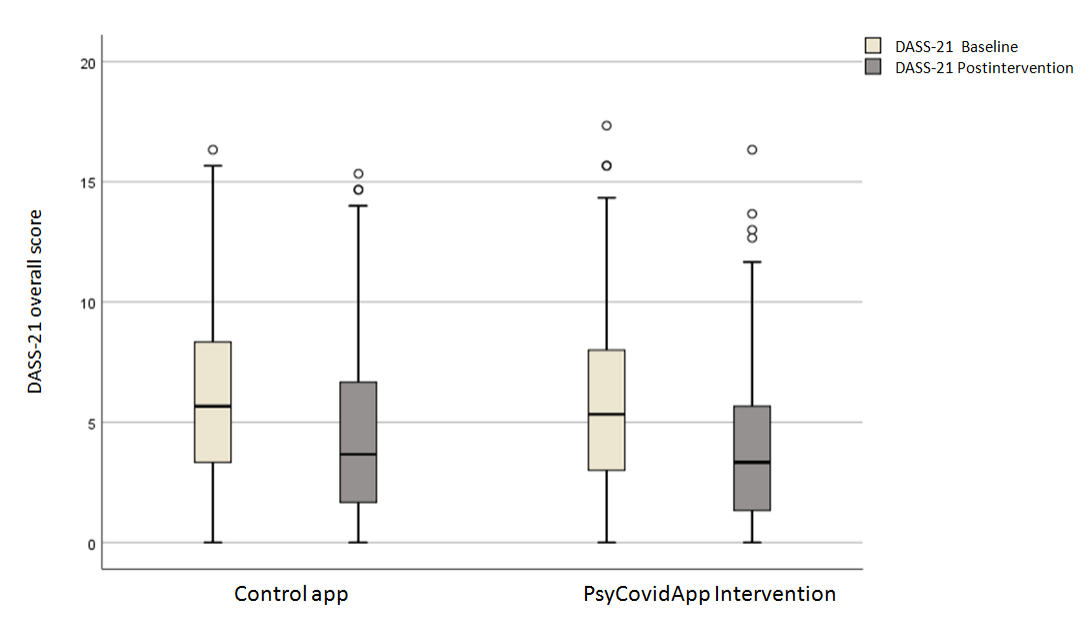
Changes in median DASS-21 scores over time, with the raw data plot of the median DASS-21 scores. Baseline scores were recorded before randomization.

Primary and secondary outcome data were available for 436 of the 482 participants (90.5%): 221 of 248 (89.1%) health care workers in the PsyCovidApp intervention group versus 215 of 234 (91.9%) in the Control App group. For the primary outcome, scale scores were lower at 2 weeks than at baseline in the PsyCovidApp and Control App groups (Figure 2). Similar reductions were observed at 2 weeks in both groups for all the secondary outcomes except for self-efficacy and depersonalization.

The effect sizes for all outcomes are shown in Table 3, Table 4, and Multimedia Appendix 5. For the primary outcome, no significant differences in the DASS-21 overall score were identified between the groups at 2 weeks (standardized mean difference –0.04; 95% CI –0.11 to 0.04; *P*=.15). Similarly, none of the secondary outcomes significantly differed between groups at 2 weeks (all *P*>.05).

**Table 3:** Descriptive summaries of the primary and secondary outcome measures at baseline and 2 weeks for the PsyCovidApp and Control App groups.

Measure		At baseline										At 2 weeks							
		PsyCovidApp group (n=248)			Control App group (n=234)			Overall (N=482)			PsyCovidApp group (n=221)				Control App group (n=215)			Completers at follow-up (n=436)	
		Mean (SD)	Range	Mean (SD)		Range	Mean (SD)		Range	Mean (SD)			Range	Mean (SD)		Range	Mean (SD)		Range
**Primary outcome**																			
	DASS-21^a^ overall score	5.84 (3.85)	0-17.33	6.14 (3.77)		0-16.33	5.99 (3.81)		0-17.33	3.83 (3.21)			0-16.33	4.27 (3.47)		0-15.33	4.05 (3.35)		0-16.33
**Secondary outcomes**																			
	DASS-21 depression subscale	4.46 (4.13)	0-18	4.58 (4.02)		0-15	4.51 (4.07)		0-18	2.97 (3.49)			0-17	3.05 (3.65)		0-18	3.01 (3.56)		0-18
	DASS-21 anxiety subscale	4.35 (3.86)	0-16	4.70 (4.25)		0-18	4.52 (4.06)		0-18	2.21 (2.43)			0-15	2.84 (3.36)		0-17	2.64 (3.13)		0-17
	DASS-21 stress subscale	8.75 (5.07)	0-21	9.15 (4.63)		0-19	8.94 (4.86)		0-21	6.11 (4.50)			0-21	6.94 (4.68)		0-21	6.51 (4.60)		0-21
	Posttraumatic stress (DTS^b^)	34.57 (23.47)	0-117	36.91 (23.18)		0-100	35.71 (23.33)		0-117	24.91 (20.41)			0-96	26.36 (21.02)		0-91	25.62 (20.70)		0-96
	Burnout (MBI-HSS^c^) emotional exhaustion subscale	23.27 (12.20)	0-54	23.57 (12.34)		0-54	23.41 (12.26)		0-54	19.43 (12.25)			0-51	19.67 (12.91)		0-54	19.54 (12.57)		0-54
	Burnout (MBI-HSS) professional accomplishment subscale^d^	39.69 (6.43)	10-48	39.59 (6.62)		15-48	39.64 (6.52)		10-48	40.33 (6.31)			13-48	39.54 (6.93)		15-48	39.94 (6.63)		13-48
	Burnout (MBI-HSS) depersonalization subscale	4.69 (5.08)	0-29	5.24 (5.41)		0-23	4.95 (5.25)		0-29	4.51 (4.96)			0-29	4.78 (5.25)		0-23	4.64 (5.10)		0-29
	Insomnia (ISI^e^)	9.80 (6.19)	0-26	10.16 (6.53)		0-27	9.98 (6.36)		0-27	8.07 (6.18)			0-28	8.44 (6.68)		0-23	8.25 (6.43)		0-28
	Self-efficacy (GSE^f^)^d^	32.42 (4.71)	19-40	32.00 (4.73)		18-40	32.21 (4.72)		18-40	33.22 (4.65)			18-40	32.54 (4.88)		17-40	32.88 (4.77)		17-40

^a^DASS-21: Depression, Anxiety, and Stress Scale-21.

^b^DTS: Davidson Trauma Scale.

^c^MBI-HSS: Maslach Burnout Inventory - Human Services Survey.

^d^Scale scores are reversed to homogeneously convey a similar treatment effect.

^e^ISI: Insomnia Severity Index.

^f^GSE: General Self-Efficacy Scale.

**Table 4:** Comparison of outcome measures between the PsyCovidApp and the Control App groups at 2 weeks. Data are adjusted standardized between-group mean differences with 95% CIs in parentheses. *P* values are not adjusted for multiple testing.

Measure	Sample of completers at follow-up (n=436)		Overall sample^a^ (N=482)	
	Adjusted standardized between-group mean differences (95% CI)	*P* value	Adjusted standardized between-group mean differences (95% CI)	*P* value
DASS-21^b^ overall score	–0.04 (-0.11 to 0.04)	.16	–0.04 (–0.11 to 0.04)	.15
DASS-21 depression subscale	0.00 (–0.07 to 0.08)	.47	0.00 (–0.07 to 0.08)	.47
DASS-21 anxiety subscale	–0.04 (–0.12 to 0.04)	.15	–0.04 (–0.12 to 0.04)	.17
DASS-21 stress subscale	–0.06 (–0.14 to 0.01)	.06	–0.06 (–0.14 to 0.01)	.05
Posttraumatic stress (DTS^c^)	0.00 (–0.06 to 0.06)	.47	0.00 (–0.06 to 0.07)	.47
Burnout (MBI-HSS^d^) emotional exhaustion subscale	0.01 (–0.06 to 0.08)	.38	0.01 (–0.06 to 0.08)	.39
Burnout (MBI-HSS) professional accomplishment subscale^e^	–0.04 (–0.12 to 0.03)	.13	–0.05 (–0.12 to 0.03)	.12
Burnout (MBI-HSS) depersonalization subscale	0.01 (–0.06 to 0.09)	.36	0.01 (–0.06 to 0.09)	.36
Insomnia (ISI^f^)	0.01 (–0.05 to 0.07)	.38	0.01 (–0.05 to 0.07)	.38
Self-efficacy (GSE^g^)^e^	–0.02 (–0.10 to 0.05)	.26	–0.02 (–0.01 to 0.05)	.27

^a^Overall sample, derived by multiple imputation (50 imputations).

^b^DASS-21: Depression, Anxiety, and Stress Scale-21.

^c^DTS: Davidson Trauma Scale.

^d^MBI-HSS, Maslach Burnout Inventory - Human Services Survey.

^e^Scale scores reversed to homogeneously convey a similar treatment effect.

^f^ISI: Insomnia Severity Index.

^g^GSE: General Self-Efficacy Scale.

**Figure 3:**
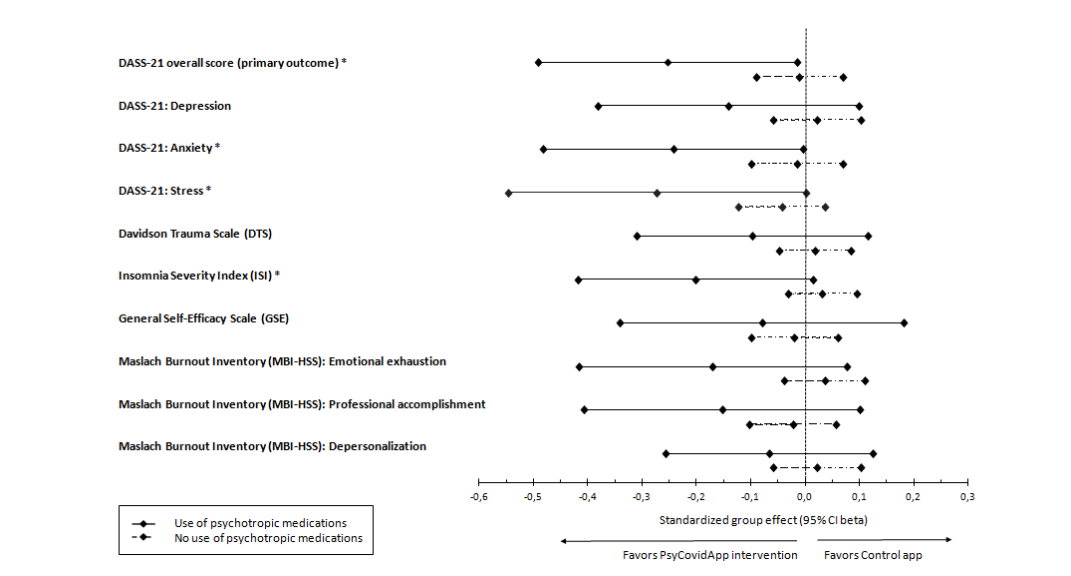
Standardized mean differences for primary and secondary outcomes in healthcare workers reporting the use of psychotropic medications at baseline. Forest plot of standardized group differences between PsyCovidApp and Control App groups for all outcomes, whereby an effect lower than 0 favored the PsyCovidApp group. Error bars show 95% Confidence Intervals (CIs). DASS-21, Depression, Anxiety, and Stress Scale. *P<.05.

**Figure 4:**
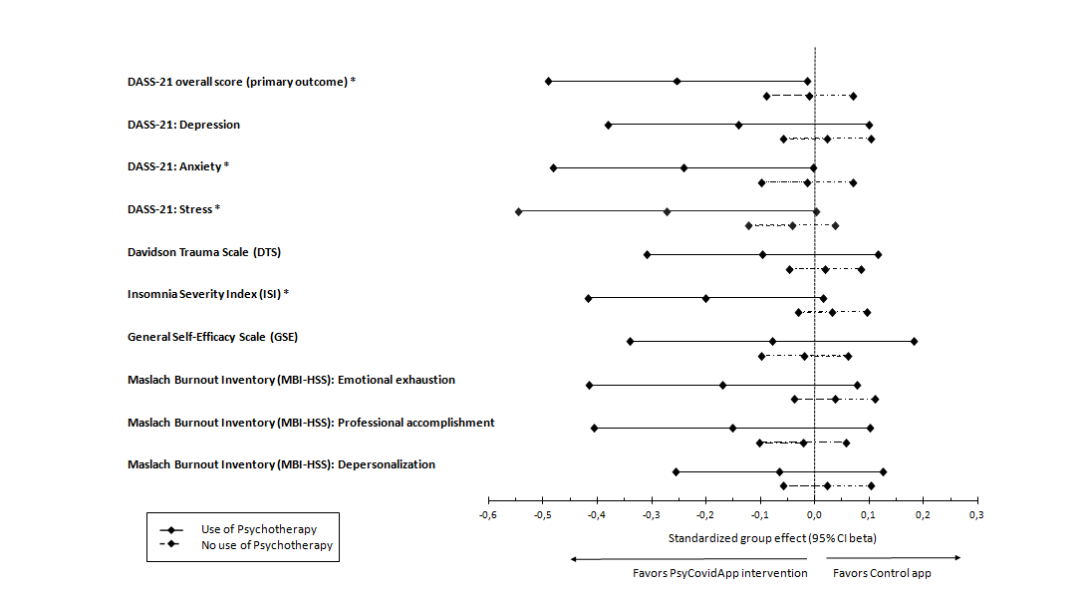
Standardized mean differences for primary and secondary outcomes in health care workers reporting the use of psychotherapy at baseline. Forest plot of standardized group differences between the PsyCovidApp and Control App groups for all outcomes, whereby an effect lower than 0 favored the PsyCovidApp group. Error bars show 95% CIs. DASS-21: Depression, Anxiety, and Stress Scale. *P<.05.

The impact of the intervention on prespecified subgroups of health care workers is presented in Figures 3 and 4 and Multimedia Appendix 3. In the subgroup of health care workers consuming psychotropic medications (n=79) (Figure 3), the PsyCovidApp group presented significantly lower DASS-21 overall scores (suggesting improved mental health) at 2 weeks than the Control App group (adjusted standardized mean difference –0.29; 95% CI –0.48 to –0.09; *P*=.004). Compared to the control app, the PsyCovidApp intervention significantly improved symptoms of anxiety (–0.26; 95% CI –0.45 to –0.08; *P*=.004), stress (–0.30; 95% CI –0.50 to –0.09; *P*=.003), posttraumatic stress (–0.20; 95% CI –0.37 to –0.03; *P*=.01), and insomnia (–0.16; 95% CI –0.30 to –0.02; *P*=.01); meanwhile, no differences were observed for symptoms of depression, emotional exhaustion, professional accomplishment, depersonalization, or self-efficacy (all *P*>.05). No significant differences were observed in any of the outcomes in the group of health care workers not consuming psychotropic medications (n=403). The interaction *P* values for anxiety, stress, posttraumatic stress, and insomnia were all <.05, suggesting that the apparent interaction was not a chance finding.

In the subgroup of participants receiving psychotherapy (n=43) (Figure 4), the PsyCovidApp group presented significantly lower DASS-21 overall scores (suggesting improved mental health) at 2 weeks than the Control App group; adjusted standardized mean difference –0.25; 95% CI –0.49 to –0.02; *P*=.02). Compared to the control app, the PsyCovidApp intervention significantly improved symptoms of anxiety (–0.24; 95% CI –0.48 to 0.00; *P*=.02), stress (–0.27; 95% CI –0.55 to 0.001; *P*=.02), and insomnia (–0.20; 95% CI –0.42 to 0.02; *P*=.03); meanwhile. no statistically significant differences were observed for symptoms of depression, posttraumatic stress disorder, emotional exhaustion, professional accomplishment, depersonalization, or self-efficacy (*P*>.05). No statistically significant differences were observed in any of the outcomes in the group of health care workers not receiving psychotherapy (n=439). The interaction *P* values for anxiety, stress, and insomnia were <.05.

No statistically significant differences (*P*>.05) were observed in the primary outcome or in any of the secondary outcomes examined in the subgroups of health care workers with higher and lower baseline DASS-21 scores (based on baseline DASS-21 median overall score).

The usability of the PsyCovidApp intervention is described in Table 5. In general, participants perceived that PsyCovidApp was highly usable (mean overall usability score 87.21/100; SD 12.65). After the trial, 208 of the 221 participants in the intervention group (94.1%) asked the research team if they could regain access to the PsyCovidApp intervention.

**Table 5:** Usability of the PsyCovidApp intervention by the PsyCovidApp group (n=221) measured with the System Usability Scale (the theoretical score range is 0-4 for single items; higher scores are indicative of higher usability).

Item	Mean score (SD)
I would like to use this App frequently	3.00 (1.02)
The App is not unnecessarily complex	3.39 (1.03)
The App is easy to use	3.56 (0.88)
No need for the support of a technical person to use this App	3.73 (0.81)
The various functions in this App are well integrated	3.40 (0.83)
Not too much inconsistency in this App	3.61 (0.81)
Most people would learn to use this App very quickly	3.43 (0.82)
The App is not very cumbersome to use	3.70 (0.70)
Confidence using the App	3.35 (0.95)
No need to learn a lot of things to use the App	3.69 (0.84)
Overall usability score^a^	87.21 (12.65)

^a^Overall System Usability Scale theoretical score range: 0-100 (higher scores are indicative of higher usability).

The sensitivity analysis of all outcomes on a complete case basis (ie, without imputation or adjustment for baseline predictors of missingness) is shown in Table 4. The results of these sensitivity analyses had little effect on the results, with similar findings for the primary outcome and the secondary outcomes when compared with the results of the main analyses.

## Discussion

### Principal Results

The global health emergency generated by the COVID-19 pandemic is posing an unprecedented challenge to frontline health care workers, who are facing high levels of workload under psychologically difficult situations with scarce resources and support. To our knowledge, this is the first randomized controlled trial to date to assess the efficacy of a mental mHealth intervention for frontline health care workers fighting the health emergency generated by the COVID-19 pandemic. Our analysis showed that at 2 weeks, PsyCovidApp only produced significant improvements in the primary and secondary outcomes of health care workers who were receiving psychotherapy or psychotropic medications.

### Implications for Clinical Practice

Although the results of our trial indicate that the PsyCovidApp intervention was not effective in comparison with the control app in the overall health care worker population, we cannot rule out the possibility that the intervention produced beneficial effects that our trial was not able to detect for various reasons, including the choice of an active comparator and the level of use of the intervention. Concerning the choice of an active comparator, most of the outcomes at 2 weeks improved similarly in both the intervention and control group. The improvements in the Control App group could be attributed to the natural progression of the disease in a context of decreasing levels of external stressors (as the impact of the first wave of COVID-19 in Spain was starting to decrease by the time the trial was initiated). However, it is also plausible that the intervention in the control group (which consisted of a similar app but with access limited to general information and contents) also had a positive effect. The impact of the control app may have been enhanced by the Hawthorne effect [40] because the stimulus introduced by the control app could have induced a positive behavioral change due to awareness of being observed. Therefore, we cannot rule out the possibility that the use of a passive comparator (eg, waiting list) in our trial may have resulted in a different outcome for PsyCovidApp.

It is plausible that the intervention did not produce the desired effects because of the short trial duration (ie, too short for the intervention to produce the intended benefits). During the time of the study, health care workers in Spain were overwhelmed with heavy workloads, and it is likely that a large proportion struggled to find time to use PsyCovidApp during the 2-week intervention period. Suboptimal use of mental health apps is indeed a widely acknowledged challenge: user retention rate for smartphone apps in the general population is low, and approximately 25% of users abandon apps after one use [41]. As pointed out by a recent systematic review of apps for depression and anxiety, more than 70% of users stopped using mental health apps after 6 weeks [42]. Unfortunately, we were not able to register the time of use, and we were therefore not able to explore a potential dose-effect relationship.

In any case, as it stands, the trial showed that the PsyCovidApp intervention did not produce significant improvements in the primary and secondary outcomes in the overall population when compared with a control app. It could be interpreted that the PsyCovidApp intervention was not effective in improving mental health outcomes in the short term in this specific population and context. It could be argued that considering all the issues health care workers are required to deal with, providing psychological aid only through a mHealth intervention may not be sufficient to produce significant improvements.

In our subgroup analyses, we observed that the intervention did not produce significant effects among those health care workers using the intervention in absence of additional mental help. This finding is consistent with findings from a recent systematic review, which showed a lack of effect of mental mHealth interventions when used as a standalone therapy [43]. However, PsyCovidApp was effective in improving the primary outcome and some secondary outcomes when used in conjunction with evidence-based treatments (such as psychotherapy or psychotropic medications). This result supports findings from a recent trial, which showed that a web-based psychoeducation approach was not sufficiently effective in improving depressive symptoms in a general population of workers but was effective for workers who had recently sought help for mental health [11]. It also supports findings from another recent trial, which reported that a web-based psychoeducational intervention produced a significantly greater reduction in depression severity among participants who had undergone psychotherapy before enrolling in the study [12]. According to the ICEMAN criteria (Multimedia Appendix 6), the results of our subgroup are highly credible because they were correctly hypothesized a priori, are supported by prior evidence [11,12], are supported by tests of interaction, are the result of analyzing a small number of effect modifiers, and show consistent effect modification across related outcomes. We hypothesize that the level of motivation to engage with the PsyCovidApp intervention may account for some of the differences in the effects observed among health care workers using and not using psychotherapy or psychotropic medications. Motivation has indeed been identified as a key element in behavior change interventions. Following the transtheoretical model of behavior change [44], health care workers using psychotherapy and psychotropic medications may be more likely in the “action” stage (as they had already made specific overt modifications in acquiring new healthy behaviors) and therefore may have been more motivated to engage with PsyCovidApp, whereas the rest of the health care workers may be more likely in the “precontemplation” or “contemplation” stages (ie, not intending to take action in the foreseeable future) and therefore may have been less motivated to engage with and follow the techniques recommended by PsyCovidApp. Future studies are needed to analyze this hypothesis. However, altogether, our results suggest that although the mHealth interventions may not be effective as a standalone strategy, one possibility to benefit from apps could be to integrate them into a clinical setting and use them in conjunction with other evidence-based treatments. This is a relevant field that is still not well understood and should be further investigated.

The fact that female health care workers were overrepresented in our trial (83% in our trial vs 68% overall in Spain [45]) could have biased our results if the intervention had produced a differential effect by gender. However, as far as we know, there is no evidence in the literature that such a difference exists. In our postprotocol subgroup analyses, we did not find gender differences in any of the outcomes considered (results not shown); therefore, this overrepresentation of female participants is unlikely to have substantially influenced the results of our trial.

PsyCovidApp presented a high usability level, with an overall score of 87.2 points—clearly above the threshold of 68 points used to determine high usability [24]. Usability factors have been widely recognized as key factors to enhance the acceptance of information and communication technologies tools. According to the technology acceptance model, the intention to use a product in the future is strongly correlated with its ease of use [46]. We observed that more than 90% of the participants asked to regain access to the PsyCovidApp intervention after the trial. This finding supports the correlation of ease of use and intention to use, and at the same time, it suggests that the intervention was acceptable and perceived as useful.

In terms of future research needs, it is worth noting that PsyCovidApp was based on a group of software features related to the intervention (eg, learning and in situ use) and communication (eg, prompting) deployed in smartphone interventions that mostly mimic more traditional mobile phone and mHealth solutions. More innovative use of the capabilities of smartphones, such as sensing, alternative delivery paradigms, and advanced analytics, could have produced a more beneficial effect. The possibilities of current smartphone technology have only just been tapped, and further research is needed to explore them fully, as are studies to rigorously analyze the empirical effectiveness of these systems.

A process evaluation is now underway, which will shed light on the mechanisms and contexts in which the intervention did or did not work. In this process evaluation, we will retrospectively investigate the “reach” of interventions (the extent to which the study participants came into contact with the intervention and how they did so). Although a recent meta-analysis of a range of mental health apps concluded that age does not impact treatment effect [19], we cannot rule out the possibility that in our trial, older professionals experienced more problems engaging with PsyCovidApp than younger participants. Therefore, as part of the process evaluation, we will specifically examine the extent to which intervention engagement differed across age groups. We will also use qualitative research methods to gain a deeper understanding of implementation barriers and facilitators and to identify suggestions about how to improve the intervention to maximize its effects on a broader range of health care workers. Once PsyCovidApp becomes publicly available, we will prospectively follow up with new users to identify patterns of use associated with higher intervention benefits. The findings of this process evaluation will inform future developments of the PsyCovidApp intervention.

### Limitations

The study has several limitations. First, the 2-week follow-up period may not have been sufficient to detect clinically meaningful differences in mental health. A longer period of time may be needed to produce the desired positive effects. There were two main reasons for this short follow-up period: (1) according to available literature [42], the use of mental health apps substantially decreases after the initial weeks, and (2) the short follow-up allowed us to obtain evidence in a timely manner, which was critical to inform decisions about scaling up the intervention in a time when health care workers in Spain were experiencing remarkably high prevalence rates of stress, anxiety, posttraumatic stress disorder, and insomnia. Second, the mental health of the participants was not evaluated through a diagnostic clinical interview but rather using instruments indicated for symptomatology assessment rather than for clinical diagnosis. Third, we did not restrict our sample to health care workers with mental health problems at baseline. Including a large proportion of participants with no (or minor) mental health problems in our study may have limited our ability to observe mental health improvements. Fourth, we did not include a waiting list or treatment-as-usual control group; thus, we are unable to determine whether the apparent reduction in mental health symptoms in both groups at 2 weeks represents equal effectiveness of the treatment allocations or the natural progression of the symptoms. Fifth, it was technically unfeasible to monitor use of the intervention, which prevented us to explore a dose-response effect. Finally, our study was remarkably specific to the current pandemic context, which limits the external validity of our results. The trial population was restricted to health care workers who had provided direct health care to patients with COVID-19. The intervention was specifically designed to address the most common mental health problems experienced under these special circumstances, included specific content acknowledging the key challenges health care workers face during the COVID-19 situation, and provided recommendations about how to overcome them. Therefore, and according to best practice guidelines [47], for the investigation of the impact of mobile health interventions in health care workers in a broader, nonpandemic context, the findings of our trial should only be taken into consideration as indirect evidence.

### Strengths

The strengths of this study include the pragmatic design, large sample size, and high follow-up rates. Moreover, the trial participants, outcome assessors, and data analysts of the research were blinded to the intervention allocation to reduce biases in the evaluation of the effects of the intervention. A common limitation of previous mHealth trials is that researchers do not have control of the proportion of participants having actual access to their interventions. In our trial, we ensured that all participants successfully downloaded and activated the app before their enrollment in the trial, which is a novel and important strength.

### Conclusion

For the first time, the PsyCovidApp trial studied the impact of a cognitive behavioral therapy and mindfulness-based mHealth intervention specifically designed to protect the mental health of health care workers fighting on the front lines of the COVID-19 pandemic. No significant differences were observed between the intervention and control groups at 2 weeks in the primary outcome and in the rest of the outcomes. However, significant improvements were observed among health care workers who were consuming psychotropic medications or receiving psychotherapy in the primary outcome, as well as in posttraumatic stress, insomnia, anxiety, and stress. PsyCovidApp may therefore improve mental health among health care workers who are already using other effective interventions, such as psychotherapy or pharmacological treatments.

### Data Sharing

Deidentified data collected for the study, including individual participant data and a data dictionary defining each field in the set, will be made available to others upon request to the corresponding author, following a signed data access agreement.

## Appendix

Multimedia Appendix 1 Content of the PsyCovidApp intervention.

Multimedia Appendix 2 Content of the control app.

Multimedia Appendix 3 Subgroup analyses: comparison of outcome measures between the PsyCovidApp and the Control App groups at 2 weeks in prespecified subgroups (high DASS-21 baseline scores; users of psychotherapy; consumers of psychotropic medications).

Multimedia Appendix 4 Number of health care workers recruited by Spanish region.

Multimedia Appendix 5 Trial results reported in terms of adjusted mean between-group differences (overall sample).

Multimedia Appendix 6 Instrument to Assess the Credibility of Effect Modification Analyses (ICEMAN) used in the study.

Multimedia Appendix 7 CONSORT-EHEALTH checklist (v 1.6.1).

## Abbreviations

DASS-21: Depression, Anxiety, and Stress Scale
DTS: Davidson Trauma Scale
GSE: General Self-Efficacy Scale
IdISBa: Balearic Islands Health Research Institute
ISI: Insomnia Severity Index
MBI-HSS: Maslach Burnout Inventory - Human Services Survey
mHealth: mobile health
SUS: System Usability Scale
