# Supplementary material for: A Mobile Phone–Based Intervention to Reduce Mental Health Problems in Health Care Workers During the COVID-19 Pandemic (PsyCovidApp): Randomized Controlled Trial
Source: JMIR Mhealth Uhealth. 2021 May 18;9(5):e27039. doi: 10.2196/27039 (PMC8133164; doi:10.2196/27039)
Supplement: Multimedia Appendix 1 [file mhealth_v9i5e27039_app1.pdf]

Multimedia Appendix 1. Content of the PsyCovidApp intervention

0. INTRODUCCIÓN

0.1 MENSAJE DE BIENVENIDA

Estimado compañero/a: ser profesional sanitario y estar expuesto a diferentes situaciones durante la actual pandemia del COVID-19, tales como cuidar de los demás, tener mucha carga de trabajo, situaciones nuevas y a veces confusas, no disponer de todo el material necesario, entre otros, puede generar estrés, ansiedad, miedo y otras emociones intensas. La forma en que gestionas estas emociones puede afectar a tu bienestar y a diferentes áreas de tu vida tanto dentro como fuera del trabajo. Estás desempeñando un rol fundamental en la lucha contra esta pandemia y estás haciendo lo mejor que puedes con los recursos disponibles.

En la situación de crisis sanitaria que estás viviendo, es importante que sepas reconocer los signos de estrés, que aprendas medidas para desarrollar resiliencia y manejar el estrés, y acudir a buscar ayuda en caso de necesitarla.
Para ayudarte en ello, un equipo de psicólogos de varios centros de investigación de España hemos diseñado PsiCovidApp, un programa de ayuda psicológica al que se accede a través de la aplicación Clinicovery.

El objetivo de PsiCovidApp es poner a tu disposición información y recursos clave para prevenir o contribuir a la mejora de posibles problemas de salud mental tales como la depresión, el estrés post-traumático, *burnout* o ansiedad, los cuales aparecen con frecuencia en sanitarios al frente de crisis como la actual. Esta herramienta puede ayudarte tanto si has percibido síntomas de este tipo como si no.
Para que te sirva de ayuda, es necesario que revises en profundidad el contenido de esta App, te recomendamos que lo hagas en varias ocasiones y aproveches los recursos prácticos que te ofrecemos. Ello es especialmente importante en estos momentos, ya que como sabes PsiCovidApp está actualmente en fase de evaluación, a través del ensayo clínico en el que estás participando.
En el módulo siguiente te explicamos brevemente el contenido de la App y cómo utilizarla, para que le saques el máximo provecho.
¡Esperamos serte de ayuda!

0.2. GUÍA DE CONTENIDOS

Los contenidos de PsiCovidAPP se agrupan en 4 grandes apartados. Para cada uno, hay varios módulos específicos con información y/o recursos. Te recomendamos empezar revisando el módulo 1.1 (Formación emocional: comprendiendo las emociones), que sentará las bases para que puedas seguir por el resto de contenidos.

A continuación, te presentamos los cuatro apartados son sus módulos correspondientes y una breve descripción de lo que encontrarás en cada uno. En el menú principal podrás identificar todos los módulos de un mismo Apartado por su icono:

- Apartado 1: Formación emocional
- Apartado 2. Estilos de vida saludables
- Apartado 3. Trabajo
- Apartado 4. Apoyo social

Apartado 1: Formación emocional

- Módulo 1.1 Información dirigida a conocer e identificar las emociones más comunes que puedes estar experimentando en estos momentos. ¡Te recomendamos que empieces por aquí!
- Módulo 1.2 Breve introducción al Mindfulness y tres audios para que puedas iniciarte en su práctica. ¡No dejes de visitarlo y prueba con la práctica!

- Módulo 1.3 Un conjunto de estrategias y recomendaciones muy prácticas para la regulación de tus emociones. ¡Te pueden ser de gran utilidad!
- Módulo 1.4 Consejos y herramientas para poder mejorar un momento de crisis y/o bloqueo emocional momentáneo. Seguro que encuentras los que mejor se adaptan a tu situación.

Apartado 2. Estilos de vida saludables

- Módulo 2.1. Información sobre qué entendemos por estilos de vida saludable, y cuáles son los que están más relacionados con tu bienestar psicológico: actividad física, alimentación, exposición a la luz solar, sueño y hábitos tóxicos. Encontrarás también algunos enlaces interesantes que te ayudarán a evaluar cómo te encuentras en estos aspectos.
- Módulo 2.2 Ideas prácticas para fomentar los estilos de vida saludables. Visítalo, seguro que encontrarás ideas y recursos muy interesantes.

Apartado 3. Aspectos laborales

- Módulo 3.1 Contenidos relativos al concepto de estrés laboral y consejos prácticos para aprender a manejarlo.
- Módulo 3.2 Contenidos relativos al concepto de *burnout* (estar quemado).

Apartado 4. Apoyo social

- Módulo 4.1 Contenidos relativos a los diferentes tipos de apoyo social existentes, ¡encuentra el que más te puede ayudar!
- Módulo 4.2 Encontrarás dos interesantes recursos web que te ayudará a profundizar en el concepto de apoyo social, además de una serie de consejos, estrategias o habilidades para conseguir apoyo social y para que los puedas integrar en tu código de conducta social.

0.3. ¿CÓMO USAR PSICOVIDAPP?

Puedes instalar PsiCovidApp en cualquier dispositivo móvil con sistema operativo Android o iOS (smartphone o tableta).

En la página principal encontrarás los diferentes módulos a los que puedes acceder.

Una vez dentro, para navegar por el contenido, desplázate con los botones de ATRÁS y SIGUIENTE (o *BACK - NEXT)* que encontrarás en la parte inferior de la pantalla.

Para los contenidos más extensos, desplázate arriba y abajo en la pantalla para verlos completos *(scroll*).

Puedes encontrar enlaces que te redirigen a una página diferente fuera de la App, podrás volver a ella con el botón de *atrás* de tu propio teléfono.

Si la letra te resulta muy pequeña o te cuesta leerlo, puedes ampliar el tamaño del texto desde la propia configuración de tu teléfono. Accede a "*Tamaño de la Fuente", "Tamaño del contenido de pantalla" o " Tamaño de texto y visualización"* (varía según el tipo de dispositivo).

Sobre los permisos que solicita la APP.

Al instalar PsiCovidApp y/o acceder a este primer contenido, es posible que se te hayan solicitado algunos permisos. Estos permisos **NO** son necesarios para el correcto uso de la aplicación. El permiso que te podría resultar útil es "**acceder a las carpetas**", ya que permite hacer uso de las carpetas para almacenar el contenido

de la App de forma temporal, y que la aplicación pueda funcionar offline (sin necesidad de tener accesos a internet).

Puedes denegar los permisos, sin que ello resulte en ningún perjuicio. Para evitar que la App te los solicite constantemente, te recomendamos que deniegues el permiso de forma permanente.

0.4. ACERCA DE PSICOVIDAPP

PsiCovidApp es una herramienta que ha sido desarrollada por un grupo de psicólogos expertos de diversos centros de investigación de varias Comunidades Autónomas, incluyendo el Instituto de Investigación Sanitaria de las Islas Baleares, el Hospital Universitario Son Espases, y la Universidad de las Islas Baleares (Islas Baleares), el Hospital Sant Pau (Barcelona, Cataluña), Hospital Miguel Servet (Zaragoza, Aragón) y la Escuela Andaluza de Salud Pública (Andalucía).

PsicovidApp ha sido desarrollada gracias al programa de financiación impulsado por la Consejería de Salud de las Islas Baleares, a través de la convocatoria competitiva “Proyectos Innovadores para hacer frente a la enfermedad COVID-19”, gestionada por parte del Instituto de Investigación Sanitaria Islas Baleares (proyecto COVID-19/06) y por la Fundación Española de Psiquiatría y Salud Mental, que junto a la sociedad Española de Psiquiatría, la Sociedad Española de Psiquiatría Biológica y la Fundación Johnson & Johnson, han financiado la fase final de este proyecto.

Equipo de profesionales de la salud que han desarrollado los contenidos de PsiCovidApp:

Maria Jesús Serrano Ripoll. Doctora en Psicología. Instituto de Investigación Sanitaria de las Islas Baleares. Editora.

Elena Gervilla . Doctora en Psicología. Universidad de las Islas Baleares.

Rafael Jiménez. Doctor en Psicología. Universidad de las Islas Baleares.

Mauro García Toro. Doctor en Psiquiatría. Universidad de las Islas Baleares.

Carolina Sitges Quirós. Doctora en Psicología. Universidad de las Islas Baleares.

Miquel Bennasar-Veny. Doctor en Ciencias Biosociosanitarias. Universidad de las Islas Baleares.

Aina María Yáñez. Doctora en Medicina y Cirugía. Universidad de las Islas Baleares.

Javier García Campayo. Doctor en Psiquiatría. Hospital Universitario Miguel Servet.

Esther García Buades. Doctora en Psicología. Universidad de las Islas Baleares.

Entidades colaboradoras

Institut d'Investigació Sanitària Illes Balears

GOIB Conselleria Salut i Consum
Gerència Atenció Primària Mallorca

UIB Universitat de les Illes Balears

Escuela Andaluza de Salud Pública
Consejería de Salud y Familias

Hospital de la Santa Creu i Sant Pau
Universitat Autònoma de Barcelona

salud servicio aragonés de salud
Hospital Universitario Miguel Servet

MÓDULO 1.1. FORMACIÓN EMOCIONAL

En este módulo de **Formación emocional** encontrarás información relativa a los estados emocionales que más frecuentemente aparecen en situaciones de crisis sanitaria como la que estás viviendo, así como para ayudarte a su identificación, como paso previo a su comprensión.

Este módulo es el primer paso para aprender a gestionar las emociones, mediante estrategias y habilidades específicas, que te iremos contando a lo largo de los módulos siguientes.

¿Conoces el concepto de **resiliencia**? Lo definimos como el proceso de adaptarse bien ante la adversidad, el trauma, la tragedia, las amenazas o las fuentes importantes de estrés, como la pandemia actual que estás viviendo. A través de estos módulos intentaremos ayudarte a aumentar tu resiliencia, ¡déjanos ayudarte!

1. ¿Por qué son buenas las emociones?

Como profesional sanitario al frente de una crisis de grandísima magnitud como la causada por Covid19, es natural sentir estrés, ansiedad, dolor y preocupación, tanto durante, como después de la crisis. Cada persona puede reaccionar de manera diferente, y tus propios sentimientos cambiarán con el tiempo. Observa, gestiona y acepta cómo te sientes. Cuidar de tu salud emocional durante esta emergencia te ayudará a pensar con claridad y reaccionar ante las necesidades urgentes de protegerte y proteger a tu familia.

Estamos soportando una gran carga emocional, a todos los niveles, por ello es bueno no dejar de lado nuestras emociones.

Las emociones forman parte de nuestra esencia y tienen tres funciones básicas:

LAS EMOCIONES COMUNICAN INFORMACIÓN E INFLUYEN EN LAS PERSONAS

• Tanto si lo deseamos como si no, la comunicación de emociones ejerce una influencia sobre los demás.

• Cuando nos encontramos en situaciones en las que es importante comunicar o enviar un mensaje, puede ser muy difícil cambiar nuestras emociones.

• Por ejemplo, nuestra expresión facial es una parte fundamental de las emociones y las expresiones faciales comunican más rápido que las expresiones verbales. A veces una expresión vale más que mil palabras, seguro que lo vives a diario en tu lugar de trabajo (al dar una buena o mala noticia, al hablar con tus compañeros, al intentar no mostrar cómo te sientes, etc).

LAS EMOCIONES ORGANIZAN Y MOTIVAN PARA LA ACCIÓN

• Las emociones suscitan respuestas específicas. Ciertas emociones se hallan ligadas "naturalmente" a determinadas respuestas. Las emociones nos preparan para la acción.

• Las emociones nos ahorran tiempo en momentos importantes en los que necesitamos actuar sin pensar.

• Las emociones fuertes nos ayudan a superar obstáculos, en nuestra mente y en nuestro entorno. Con la fuerza de nuestras emociones estamos aguantando los envites de esta pandemia.

• Las emociones excesivamente intensas pueden tener el efecto contrario, por ello aprenderemos a gestionarlas.

LAS EMOCIONES PUEDEN AUTOVALIDARSE

• Nuestras reacciones emocionales frente a los demás y frente a los acontecimientos pueden proporcionarnos información acerca de la situación. Las emociones pueden ser señales o alarmas de que algo está sucediendo. Si en algún momento tienes emociones muy intensas, pueden ser señal de que no te encuentras bien, ¡préstales atención y no las evites!

• Pero, cuando llevamos este razonamiento al extremo, tratamos las emociones como hechos, cuidado con eso: "Si me siento incompetente, es que lo soy". "Si me deprimo cuando estoy solo, no debería estar solo". "Si siento que algo está bien, es que eso es bueno". "Si tengo miedo, eso es amenazante". "Le amo, así que debe

ser la persona adecuada para mí". A lo largo de los módulos de esta App aprenderás a mejorar la gestión de estas intensas emociones.

2. La preocupación

La palabra preocupación se define como “una cadena de pensamientos e imágenes cargados de sensaciones negativas y relativamente incontrolables”.

Todo el mundo se preocupa de vez en cuando, pero hacerlo en exceso puede ser perjudicial, ya que nos hace sentir tensión y ansiedad. Las personas que se preocupan excesivamente son expertas en descubrir posibles problemas y son ineficaces a la hora de generar soluciones o respuestas de afrontamiento útiles. Aunque podríamos decirnos a nosotros/as mismos/as – “no merece la pena”, ”no lo pienses más”, ”piensa en otra cosa”, “deja de preocuparte… no tiene sentido… no servirá de nada…” - hay algo que hace que sea difícil detener esta reacción, ya que el acto de preocuparse puede resultar útil.

Preocuparse es útil si nos moviliza, si no es inútil

La preocupación es una respuesta normal ante situaciones que pueden perturbar nuestra vida. Si el problema es de una importancia considerable y la preocupación que genera es proporcionada, no solamente no debemos vivirlo como algo negativo, sino que es un afrontamiento que nos prepara para actuar adecuadamente ante el problema.

La percepción de incertidumbre, como la que estamos viviendo ahora, es una fuente importante de preocupación y ansiedad, pero el objetivo no consistirá en intentar eliminarla, sino, por el contrario, en reconocer, aceptar y desarrollar estrategias de afrontamiento cuando se presente. Se trata, por tanto, de aprender a manejarla.

¿Vale la pena preocuparse?

En esta situación de crisis sanitaria actual, es probable que hayas desarrollado preocupación excesiva por diversos temas: temor al contagio propio y de tus familiares, preocupación por los pacientes a los que atiendes, por tus compañeros de trabajo, por los cambios constantes a los que te ves sometido, etc.

Pero hay cuatro cosas de las que no vale la pena preocuparse, y sin embargo representan una gran parte de nuestras preocupaciones: lo que no tiene importancia, lo improbable, lo incierto y lo incontrolable. Si relegamos este tipo de preocupaciones a un segundo plano, nos sentiremos mejor.

a. Lo que no tiene importancia

Es fácil llenar tu vida con preocupaciones por cosas pequeñas. Pregúntate: “¿Es tan importante lo que me preocupa?” Éstos son las tres reglas que te ayudarán a responder a esta pregunta:

i. la regla de los 5 años. Pregúntate: “¿Que pasará con este asunto dentro de 5 años?”. Esta es una forma de mirar su preocupación desde una perspectiva a largo plazo.

ii. la varilla de medición. Pregúntate: “¿En una escala de malas experiencias, dónde estaría lo que me preocupa?”. Piensa en una muy mala experiencia que hayas tenido. ¿Dónde situarías tu preocupación actual en comparación con ésta?.

iii. la calculadora. Pregúntate: “¿Me preocupo mucho?, ¿Me merece la pena?”. Perdemos mucho tiempo y energía con esto. Asegúrate de que no te preocupas sobre el problema más de lo que se merece. Necesitas tu tiempo y energía para cosas más importantes.

b. Lo improbable

Muchas de las preocupaciones son “¿Qué pasaría si ...?”. Imaginamos una serie de cosas terribles que podrían ocurrir hoy o mañana, pero la mayoría de las cosas son muy poco probables. Si te permites preocuparte por lo improbable siempre encontrarás un motivo para estar preocupado/a. Abordar los problemas reales ya supone suficiente trabajo. No pierdas el tiempo, la energía y felicidad en problemas que no existen.

c. Lo incierto

Muchas veces no sabemos cómo saldrá algo. Hay muchas cosas que nos preocupan aún cuándo no han sucedido y sobre las que sólo podemos actuar una vez que ocurran. Por ejemplo, la preocupación excesiva por temor a contagiarse a diario, no va a hacer que tu actuación sea más correcta, sino que al estar más nervioso/a puede hacer que cometas más errores.

d. Lo incontrolable

Nosotros no tenemos control sobre muchas de las cosas que nos preocupan. Por ejemplo, la preocupación por que llueva en un acontecimiento familiar no cambia la probabilidad de que suceda. Nada de lo que hagas o pienses evitará que haya tormenta ese día.

3. La irritabilidad

Experimentar irritabilidad o enfado en algún momento, puede ser normal, dada la situación actual de crisis sanitaria que nos está afectando. Y decimos que es normal siempre que no interfiera en las diferentes áreas de tu vida.

El enfado y la irritabilidad excesivos pueden alterar las relaciones interpersonales y familiares, pueden tener una influencia negativa sobre el desempeño en tu trabajo, te pueden llevar a decir cosas de las que te puedes luego avergonzar o te puedes sentir culpable, disminuyen tu autoestima y te hacen sentir que no tienes control emocional.

Existen muchos **ejemplos** de esto: algunas personas que están sometidas a una sobrecarga de tareas en su trabajo pueden sentirse irritables y enfadadas con sus jefes; una persona que está sufriendo un confinamiento prolongado o una cuarentena, pueden sentir emociones de ira e irritabilidad.

La irritabilidad se convierte en un problema cuando es: demasiado intensa, sucede con demasiada frecuencia, dura demasiado tiempo, echa a perder relaciones o empleos, y particularmente, cuando conduce a violencia o agresión.

Enfadarse demasiado nunca es útil y la creencia de que expresar airadamente un enfado puede hacerle sentir mejor no es cierta.

Imagina que vas conduciendo tu vehículo y te encuentras con un atasco debido a unas obras en la carretera, puedes pensar "¡Esto es increíble!", "¡Qué poco respeto!", "¡Qué desastre de ayuntamiento!", "¡Llegaré tarde por culpa de estos operarios!". La persona que piensa de esta forma cree que las demás personas son las causantes de su problema.

Es natural creer que los sucesos externos te están molestando. Cuando te enfureces con alguien, automáticamente lo conviertes en el causante de todos tus malos sentimientos, y piensas "me estás molestando", "me está sacando de quicio". Así crees que las demás personas son las causantes de tu problema.

Un vecino podría comprarse una mascota ruidosa, una amiga puede dejarte plantada a última hora, un compañero de trabajo puede rechazar una petición que le hagas... Independientemente de lo injustos que te parezcan los demás, estas personas no pueden hacer que te sientas mal, no lo han hecho nunca ni lo harán. **La realidad es que tú eres quien genera toda la rabia que experimentas.**

No son los acontecimientos exteriores sino la percepción que tienes de ellos y los pensamientos que te provocan lo que crea tu respuesta emocional. Esto explica que la misma situación produzca en unas personas un profundo malestar y a otras le sea indiferente o que el mismo acontecimiento unas veces nos irrite y otras no.

La irritabilidad afecta al modo en que nos sentimos, pensamos y nos comportamos. Causa un gran número de síntomas en nuestro cuerpo. Puede ser provocada por nuestras reacciones a sucesos que ocurren a nuestro alrededor o por nuestros propios pensamientos y preocupaciones. En muchos casos, su irritación es

consecuencia de sutiles errores de pensamiento o distorsiones cognitivas. Muchas de éstas son retorcidas, unilaterales o simplemente erróneas. Esto ocurre sin darnos cuenta, porque siempre tenemos una misma forma de ver las cosas, como si aplicáramos una plantilla o esquema o nos pusiéramos unas gafas con cristal de un determinado color o de un determinado aumento. Aprendemos a hacerlo así, lo repetimos miles de veces y es difícil cambiarlo si no hacemos un trabajo extra.

4. El estrés

Es posible que este escenario de emergencia sanitaria actual sea único y sin precedentes para ti, especialmente si no has estado involucrado/a en situaciones similares. No obstante, las estrategias para aliviar el estrés son las mismas incluso si cambia el escenario. Cada persona reacciona de manera distinta a las situaciones de estrés. La forma en que respondes a la situación causada por la emergencia sanitaria puede depender de tus antecedentes, de los aspectos que te diferencian de otras personas y del lugar en el que vives.

Para comenzar, estos son ejemplos de pensamientos de algunas personas que están experimentando estrés, quizás te identificas con alguno de ellos:

“No consigo relajarme ni estando con mis amigos, continuamente pienso en lo que tengo pendiente por hacer”

“Antes de terminar de hacer algo ya estoy haciendo lo siguiente. Hago muchas cosas a la vez y no termino ninguna...”

“ Siempre que alguien me pide ayuda no soy capaz de negarme, por lo que nunca logro encontrar tiempo para mí, esto hace que me sienta muy cansado e irritable“

“Pienso constantemente en todas las cosas pendientes de la semana y no consigo conciliar el sueño...”

“Intento hacerlo todo perfecto, me preocupo tanto que a veces no me concentro y olvido detalles importantes…”

El estrés es la palabra que muchas personas utilizan para describir un estado en el que las exigencias de la vida se vuelven demasiado grandes como para hacerles frente. Esto varía de una persona a otra y lo que una encuentra estresante puede no serlo para otra.

Casi todas las personas han sufrido estrés en alguna ocasión, sin embargo cuando éste se mantiene de forma constante y durante mucho tiempo terminará afectando a su salud. La vida es estresante. Lo que importa es que reconozcas cuándo puede llegar a ser perjudicial en su vida.

¿Estás bajo demasiado estrés?

¿Cómo reacciona tu cuerpo?

- falta de aliento
- sensación de malestar o mareo
- dolores de cabeza
- cansancio constante
- desmayos
- inquietud
- dolores en el pecho
- problemas para dormir
- tendencia a sudar
- estreñimiento o diarrea
- tics nerviosos
- excesivo apetito o falta de apetito
- calambres o espasmos musculares
- indigestión o ardores
- hormigueos
- aumento de la tensión arterial
- problemas sexuales.

¿Cómo te sientes?

- con agresividad
- deprimido/a
- irritable
- descuidada/o
- con miedo a enfermar
- aprehensión por el futuro
- temor al fracaso
- falta de interés en tu vida
- sensación de baja autoestima
- falta de interés por las demás personas
- perder su sentido del humor
- no confiar en nadie.

¿Cómo te comportas?

- dificultad para tomar decisiones
- incapacidad para mostrar los verdaderos sentimientos
- problemas de concentración
- evitar situaciones difíciles
- negar que hay problemas
- llanto frecuente
- cambios en los hábitos alimenticios
- beber o fumar demasiado.

5. La ansiedad

Es la respuesta mental y física que se produce ante situaciones de peligro. En la situación actual de emergencia sanitaria que estamos viviendo, tener algo de ansiedad en algún momento puede considerarse una reacción normal (por ejemplo, antes de la prueba médica, al hablar con pacientes y familiares, antes de una evento importante, etc.). Nos puede dar una ayuda adicional para estar más alerta o puede darnos un mayor nivel de energía para afrontar las altas demandas que nos está generando la situación del COVID. Entendida como tal, es un mecanismo de defensa del organismo y por tanto no necesariamente negativa.

El objetivo no es suprimirla sino aprender a controlarla. Se trata de vivir con menos ansiedad y no de vivir sin ansiedad.

La ansiedad se convierte en un problema cuando los síntomas:

- Son graves y desagradables.
- Duran mucho tiempo.
- Ocurren con demasiada frecuencia.
- Aparecen ante situaciones que realmente no deberían ser estresantes.
- Nos impiden hacer lo que queremos hacer.

La ansiedad nos puede afectar en 3 áreas importantes:

1) Cómo pensamos: Cuando no se controla la ansiedad, las personas describen pensamientos como ”No voy a poder soportarlo”, ”Esto es horrible”, “Me dará un infarto”, ”Enloqueceré”. Estos pensamientos pasan por la mente automáticamente, como un flash, cuando se está ansioso.

Dos cosas deben tenerse en cuenta sobre los pensamientos automáticos:

- Que son irracionales y poco realistas – no morirás o enloquecerás.
- Son situaciones que se pueden controlar: habitualmente la mente nos advierte de un peligro mucho mayor del que en realidad tenemos que afrontar.

Aprenderemos a controlar tales pensamientos en los módulos de gestión de la ansiedad y el estrés.

2) Qué hacemos: Las personas con ansiedad a menudo evitan cosas, por ejemplo enfrentarse a situaciones laborales complicadas, posponer tareas que nos generan ansiedad, entre otras. Evitando la situación, puede que te sientas mejor y experimentes un alivio inmediato de la ansiedad que estabas sintiendo al imaginarte realizando la actividad. Pero a largo plazo la evitación siempre empeora el problema. Esto ocurre porque la ansiedad se asocia cada vez más con aquello que se evita y así se hace más difícil afrontarlo.

3) Cómo reacciona nuestro cuerpo: Hay muchos síntomas físicos de ansiedad. Surgen como una manera de preparar al cuerpo para hacer frente a una supuesta amenaza. Los síntomas son muy desagradables y algunas veces aparecen sin razón aparente, de manera que puedes pensar que padeces un problema físico grave o que algo terrible va a suceder. Es importante saber que estos síntomas no son peligrosos y no te harán ningún daño aunque sean severos.

SI TE HAS VISTO REFLEJADO/A EN ESTOS LISTADOS DE SÍNTOMAS, TE INVITAMOS VISITAR EL MÓDULO DE CONSEJOS PARA ABORDAR EL ESTRÉS

Estrés y fatiga por compasión

Experimentar o ser testigo de eventos traumáticos o que ponen en riesgo la vida afecta a cada persona de modo diferente. En algunas circunstancias, es posible manejar la angustia correctamente para reducir los resultados negativos conductuales y de salud asociados. En otros casos, algunas personas podrían experimentar niveles de angustia o deterioro clínicamente signicativos, como trastorno de estrés agudo, trastorno por estrés postraumático o estrés traumático secundario (también conocido como trauma indirecto). La fatiga por compasión y el agotamiento mental también pueden ser el resultado del estrés crónico en el lugar de trabajo y la exposición a eventos traumáticos durante la pandemia del COVID-19.

Si estás lidiando con muchas tragedias en tu día a día, lógicamente, esta situación te impactará en de forma negativa. El problema llega cuando esta afección se prolonga en el tiempo.

La fatiga por compasión se revela en forma de **tres síntomas:** la re-experimentación constante de los sucesos, el embotamiento afectivo y la hiperactivación, el estado sostenido de nervios (muy parecidos a síntomas de estrés postraumático).

SI CREES QUE ÉSTE PUEDE SER TU CASO, CONSULTA CON UN PROFESIONAL DE LA SALUD MENTAL, SEGURO QUE EN TU HOSPITAL O SERVICIO TE PUEDEN AYUDAR DE FORMA CONFIDENCIAL

6. El estado de ánimo

Los momentos de tristeza, de duda o cuestionamiento forman parte de la vida, y especialmente en la situación de crisis sanitaria que estamos viviendo. El hecho de pertenecer al colectivo de profesionales sanitarios que lucháis contra el COVID-19 te expone a situaciones complicadas en muchos aspectos. En relación con los acontecimientos que nos van sucediendo, las personas experimentamos una amplia gama de sentimientos, del más triste al más optimista. Dentro de esta variedad de emociones, la tristeza, el desánimo y la desesperación son experiencias humanas normales. Estas disminuciones en el estado de ánimo no deben confundirse con las que experimenta una persona deprimida. El hecho de sentirse triste, "deprimido", de tener "pensamientos negativos" o dificultad para dormir no significa que sufras una depresión, pero tienes que tener en cuenta que la a depresión es una enfermedad, como lo es la diabetes o una úlcera de estómago. Para hablar de depresión, y por lo tanto de enfermedad se requiere:

• que estos sentimientos se presenten de forma (casi) continua durante un período de al menos dos semanas (que uno de estos síntomas debe ser el estado de ánimo depresivo o pérdida de interés o placer);

• que causen un malestar importante en una o varias áreas de la vida diaria (dificultad o imposibilidad de levantarse, de ir a trabajar, salir a hacer las compras...).

Cualquier persona puede padecer depresión en algún momento de su vida. No obstante, la situación actual puede que aumente tus posibilidades de padecerla.

Síntomas que pueden aparecer en la depresión

• **Conducta:** Ataques de llanto, aislamiento de los demás, descuido de las responsabilidades, pérdida de interés por la apariencia, pérdida de la motivación.

• **Emociones:** Tristeza, ansiedad, culpabilidad, ira, cambios de humor, falta de emociones (embotamiento afectivo), sentimientos de desesperanza, sentimientos de incapacidad.

• **Pensamientos:** Autocrítica, sentimiento de culpa, preocupación, pesimismo, problemas de memoria, falta de concentración, dificultad para la toma de decisiones, confusión, pensamientos referentes a que otras personas te vean mal, incluso pensamientos de muerte o suicidio. Muchas veces son frecuentes las ideas de muerte, pero desaparecerán cuando mejores de tu situación. ***Si tienes miedo de perder el control y hacerte daño debes acudir de inmediato a un servicio de urgencias donde un compañero te escuchará y aconsejará lo más conveniente de forma confidencial.**

• **Síntomas Físicos:** Sensación de cansancio, falta de energía, dormir mucho o muy poco, comer en exceso o la pérdida de apetito, estreñimiento, pérdida o aumento de peso, ciclo menstrual irregular, pérdida de apetito sexual, dolores sin explicación aparente.

Hay muchos tratamientos efectivos disponibles para la depresión hoy en día. Pero a pesar de ello, muchas personas con depresión nunca buscan ayuda. **Si te has reconocido en los síntomas anteriores, busca ayuda profesional.**

Pero también te puede ser útil **identificar los signos precursores de la depresión** y así ayudarte con las estrategias y consejos que te ofrecemos a lo largo de estos módulos:

- un cambio en el estado de ánimo (principalmente tristeza y el llorar sin motivo);
- la pérdida de interés en actividades que son por lo general placenteras;
- problemas con el sueño (despertar en las primeras horas de la mañana, sueño no reparador...);
- ansiedad constante con momentos en que se agudiza, incluso en situaciones hasta ahora consideradas de rutina y sin riesgo (ir de compras, por ejemplo);
- irritabilidad inusual que requiere una gran cantidad de energía para controlarla;
- cansancio excesivo o enlentecimiento de los movimientos;
- una incapacidad para funcionar, para llevar a cabo las tareas cotidianas;
- una mayor sensibilidad al ruido;
- cambios no habituales en el apetito (disminución o aumento).

7. Angustia o daño moral

La angustia o daño moral es el resultado de tener que actuar de manera opuesta a los propios valores y creencias.

Quizá actualmente te asalten sentimientos de culpa o vergüenza por algo que hiciste, dejaste de hacer o permitiste que se hiciera en tu ámbito de competencias durante la crisis sanitaria. Actos que consideras no acordes con tus principios o valores o que simplemente crees que deberías haber sido capaz de evitar. Por ejemplo:

- Contagiarte del COVID 19 y quizá transmitir el virus a tus compañeros o fuera del entorno sanitario, incluyendo a tu familia.
- Haber faltado al trabajo por cualquier causa en días en que sabías que eras muy necesario.
- Haber cometido errores en tus tareas.
- Haber estado implicado en la atención de pacientes fallecidos que crees que quizá podrían haberse salvado con una atención más cuidadosa, o diligente, o intensiva.
- Cuando los requisitos para la propia seguridad interfieren con las necesidades de un paciente. Por ejemplo, cuando un paciente infectado con COVID-19 que no puede levantarse de la cama sin ayuda se cae inesperadamente mientras la enfermera se toma el tiempo para ponerse el EPP adecuadamente.

- En el caso de que seas médico, el no haber podido evitar la muerte de pacientes por falta de conocimientos que quizá ahora sí tengas sobre cómo manejar las complicaciones de la infección por COVID 19. También el haber decidido no intubar a los pacientes de peor pronóstico por falta de respiradores o haber limitado el uso de fármacos potencialmente útiles por su carestía, o simplemente por seguir los protocolos de tu centro.

Quizá no has compartido con nadie estos sentimientos o a lo mejor sí lo has hecho y te han intentado consolar al respecto, pero sigues sufriendo una culpa o vergüenza dolorosa.

Queremos que sepas que no estás solo, porque lo que te ocurre es frecuente en situaciones como a la que nos estamos enfrentando. Se le ha llamado "Daño moral". Se describió en veteranos de guerra y, aunque es controvertido, también se acepta como un problema frecuente en supervivientes de situaciones de crisis sanitarias tan graves como a la que tú te has enfrentado.

Déjanos compartir contigo algo que sabes, pero quizá no sientes; nadie estaba preparado para esto, ni personal ni colectivamente, y, por ello, no tiene sentido exigirse no cometer errores en estas circunstancias. De hecho, sanitarios y no sanitarios cometemos errores constantemente en todas las circunstancias, y a veces repercuten en la salud y en la vida de la gente. Bienvenido al club de los humanos. Si puedes hacer algo para reparar o compensar ese posible error adelante. Eso puede ayudarte a encontrarte mejor psicológicamente. Incluso tomar iniciativas preventivas para el futuro, o implicarte en algunas que seguro otros compañeros han iniciado, puede ser una vía para canalizar esas emociones negativas que te agobian y usarlas como combustible para algo positivo.

Mención aparte merecen los actos por acción u omisión que pudiste protagonizar y que ahora te remueven la conciencia. Entre ellos, seguir prescripciones o pautas que te parecen moralmente censurables, pero que te sentiste presionado a atender. De nuevo humildemente te sugerimos que aceptes que el trabajo del sanitario debe ser coordinado, con asunción compartida de responsabilidades, y basado en el interés de toda la población a criterio de las autoridades sanitarias. Por supuesto estas autoridades pueden cometer errores y deberán depurarse responsabilidades sobre los más graves. Pero un sanitario individual es una pieza de un enorme engranaje y, por ello, no puede hacerse responsable de sus carencias ni nadie en justicia puede hacerlo.

Existe el riesgo de que quieras lidiar con estas emociones negativas de forma escapista. Retrayéndote del contacto social, tomando alcohol en exceso u otras drogas, refugiándote en cualquier conducta o actividad de huida que pueda convertirse por ello en adictiva. No te lo aconsejamos. Es contraproducente y no resolverá el problema de fondo.

También nos atrevemos a hacerte **algunas propuestas** por si pueden ayudarte.

1. Te animamos a hablar con compañeros que merezcan tu respeto por su autoridad moral, muy especialmente si tienen un carácter acogedor y saben escuchar. También puede ayudarte un miembro de tu familia que te conozca bien y te acepte incondicionalmente, o incluso un líder espiritual o religioso que conecte con tus creencias y valores. Anímate a compartir con ellos tus sentimientos íntimos en un momento y lugar apropiados. Es muy probable que te sientas comprendido, apoyado y aliviado.
2. Otra alternativa que te puede ser útil como sustitutivo de lo anterior es plantearte en tu imaginación qué te diría una de estas personas si tuvieras ese encuentro. También algo en la misma línea: piensa qué le dirías tú a un buen amigo que hubiera pasado por el trance que tú has pasado y te expresara sentirse avergonzado o culpable. ¿Serías igual de duro que eres contigo mismo? Probablemente no. Si te ayuda este enfoque te animamos incluso a poner estas reflexiones a tu supuesto amigo por escrito y luego leerlas dejando que su mensaje te vaya calando. Con frecuencia somos nuestros mayores críticos lo cual no beneficia a nadie. Sabemos que cambiar estas pautas de relacionarnos con nosotros mismos no es fácil ni rápido, pero mucha gente lo consigue poco a poco, cada uno a su ritmo, quizá con pasos adelante y atrás. Al final algunas personas incluso consiguen llegar a ser amigas de sí mismas, lo cual les ayuda muchísimo a afrontar la adversidad.
3. Tenemos que insistir sobre el pedir y aceptar la ayuda de tu red social de apoyo como algo crucial. Si piensas que no tienes derecho a preocupar a tu familia y amigos con tu sufrimiento quizá te estés equivocando. Aunque tú no hables de ello, es muy probable que lo intuyan y lo sufran también en silencio. Si les das la oportunidad de compartir lo que te pasa quizá os aliviéis mutuamente.
4. Existe gran evidencia sobre la ayuda que aporta el ejercicio físico regular, la dieta saludable, el descanso suficiente y el contacto con la naturaleza para afrontar el malestar psicológico del tipo que sea mientras nuestra mente y nuestro cerebro van metabolizándolo. VISITA EL MÓDULO DE ESTILOS DE VIDA SALUDABLE.

5. Ciertamente el tiempo puede ayudar a cicatrizar también las heridas morales, pero si ves que pasan las semanas y los meses y eso no ocurre en tu caso, puede ser de mucha ayuda consultar a un profesional de la salud mental. Tu Colegio profesional te puede ayudar en este aspecto de forma confidencial. Recuerda: no hay nada por lo que avergonzarse.

MÓDULO 1.2 MINDFULNESS

En este Módulo vas a encontrar contenido relativo al Mindfulness. Esperamos que te sea muy útil. Te animamos a que participes, no te desanimes si al principio no va tan bien como tu esperabas, ¡si practicas da resultado!

¡Vamos a ello!

¿QUÉ ES MINDFULNESS Y POR QUÉ ES UTIL?

MINDFULNESS ES un estado de la mente que consiste en estar atento al momento presente con aceptación.

ESTE ESTADO se asocia a un gran bienestar físico y psicológico porque permite reducir, de forma importante, el estrés de la vida diaria. También es útil para el tratamiento y prevención de enfermedades psicológicas como la depresión o la ansiedad, y trastornos somáticos como la hipertensión o los cuadros dolorosos.

LA FORMA DE DESARROLLAR este estado es la práctica de mindfulness, que es una técnica para el desarrollo de la atención

¿CÓMO SE PRACTICA MINDFULNESS? Es necesario saber qué postura corporal adoptar y qué hay que hacer con la mente.

LA POSTURA recomendada es sentado, con los pies bien apoyados en el suelo, con la espalda recta sin tensión y manos apoyadas en las piernas. Los ojos es preferible que estén cerrados cuando uno empieza a practicar.

¿QUÉ HAY QUE HACER CON LA MENTE? Toda práctica de mindfulness se estructura en 4 pasos:

1.- PONER LA ATENCIÓN EN UN PUNTO DE ANCLAJE: Habitualmente se sitúa la atención en la respiración (en fosas nasales, en el centro del esternón o dos centímetros debajo del ombligo) o en el cuerpo.

2.- ESPERAR A QUE LA MENTE DIVAGUE y deje de poner la atención en ese punto.

3.- DARSE CUENTA DE QUE LA MENTE SE HA IDO DEL ANCLAJE: Es el paso más difícil, porque tendemos a quedarnos divagando durante minutos sin ser conscientes.

4.- VOLVER AMABLEMENTE LA ATENCIÓN AL PUNTO DE ANCLAJE: Este proceso se repite una y otra vez, redirigiendo continuamente la atención, el tiempo que dura la práctica.

¿Cuándo y cuánto practicar?

Al principio, con practicar 5-10 min al día es suficiente. Conforme os vayáis sintiendo a gusto, podéis alargar la sesión 5 min cada 15 días hasta llegar a los 30 min (que pueden realizarse en dos sesiones de 15 min).

A las 8-10 semanas de practicar vais a notar importantes cambios psicológicos: mayor tranquilidad y bienestar, mayor capacidad de atención y menor tendencia a la rumiación.

Ejemplos de prácticas: Incluimos en audio la práctica de mindfulness en la respiración y el *body scan*, así como la práctica de 3 minutos. Esta última es ideal para hacerla en cualquier momento del día.

Otra práctica sencilla y efectiva es contar respiraciones: En cada espiración contar mentalmente 1, 2, 3 hasta llegar a 10 y volver a empezar. Si no perdéis la cuenta ni os pasáis de 10 durante el tiempo de la práctica la atención empieza a ser estable.

Mindfulness es muy sencillo y efectivo. Millones de personas lo practican ya en todo el mundo.

La mayoría de facultades de medicina y enfermería de los paises anglosajones incluyen esta formación

MÓDULO 1.3. GESTIÓN EMOCIONAL

En este Módulo vas a encontrar contenidos relativos a habilidades y estrategias para regular tus emociones. **Gestionar tus emociones** no es una tarea sencilla, requiere de consciencia y de constancia. Las actividades y trucos que te proponemos necesitan de práctica, no te desanimes si al principio no va tan bien como tu esperabas, **¡si practicas da resultado!**

¡Vamos a ello!

1. OBSERVAR LA RESPIRACIÓN

A continuación encontrarás siete ejercicios que te ayudarán a calmarte y relajarte, están basados en observar tu respiración. Se trata de centrar tu atención en la respiración, en cómo entra y cómo sale el aire. Observar la respiración como vía para controlar tu mente sin luchar contra la realidad, aceptando lo que nos pasa.

Anímate a realizar cada uno de estos ejercicios, de forma gradual, al principio puede que te cueste un poco, pero… ¡Te serán de mucha ayuda!

a. Respiración profunda

Tumbarse sobre la espalda. Respirar suavemente centrando la atención en el movimiento del estómago. A medida que empezamos a inspirar, permitamos que nuestro estómago se expanda de manera que entre el aire en la parte baja de los pulmones. A medida que los espacios superiores de los pulmones empiezan a llenarse de aire, el pecho se eleva y el estómago baja.

No nos cansemos. Sigamos así durante 10 respiraciones. La espiración ha de ser más larga que la inhalación.

b. Medir la respiración mediante los pasos

Caminar despacio por un prado, una acera o un sendero. Respirar con normalidad.

- Determinar la longitud de la respiración, la inspiración y la espiración, mediante el número de zancadas que damos. Hacer el ejercicio durante unos cuantos minutos.

- Después, alargar la espiración **sólo** un paso. No forzar la inspiración para ponerla en consonancia con la espiración. Ser natural. Observar la inspiración con atención para comprobar si se da una tendencia a hacerla más larga. Continuar durante 10 respiraciones.

- Ahora alarguemos la espiración **un paso más**. Comprobar si la inhalación también se alarga un paso más. Sólo alargar la inhalación cuando sintamos que nos apetece hacerlo. Después de 20 respiraciones, volver a respirar con normalidad.

- Al cabo de 5 minutos, podemos intentar de nuevo alargar la respiración.

- Cuando nos sintamos un poco cansados, podremos volver a la respiración normal.

- Después de varias sesiones de practicar este ejercicio, la inspiración y la espiración tenderán a acompasarse de manera natural.

c. Contar la respiración

Sentarse en el suelo con las piernas cruzadas (si sabemos hacerlo, nos sentaremos en la posición de loto o medio loto); o sentarse en una silla con las plantas de los pies tocando el suelo (también podemos arrodillarnos, tendernos en el suelo o pasear). A medida que se inhala, prestemos atención al proceso y pensemos "Estoy inspirando, 1". Cuando se espira, estemos atentos a que "Estoy espirando, 1". Recordar que debemos respirar desde el estómago. Cuando empecemos la segunda inhalación, estemos atentos a que "Estoy espirando,2". Y espirando lentamente, estemos atentos a que "Estoy espirando, 2". Continuar hasta hacerlo 10 veces. Cuando lleguemos a 10, volver al 1. Siempre que perdamos la cuenta volvamos al 1.

d. Observar la respiración mientras se escucha música

Escuchar una melodía. Hacer respiraciones largas, suaves y regulares. Observar la respiración; seamos conscientes del movimiento y sentimiento de la música al mismo tiempo que observamos la respiración. No nos perdamos en la música, continuemos acompasando nuestra respiración.

e. Observar la respiración mientras se mantiene una conversación

Hacer respiraciones largas, suaves y regulares. Observar la respiración mientras escuchamos las palabras de un amigo y nuestras propias respuestas. Continuar como con la música.

f. Observar la respiración

Sentarse en el suelo con las piernas cruzadas (si sabemos hacerlo, nos sentaremos en la posición de loto o medio loto); o sentarse en una silla con las plantas de los pies tocando el suelo (también podemos arrodillarnos, tendernos en el suelo o pasear). Empezar inspirando suave y normalmente (desde el estómago), dándonos cuenta de cómo "Inspiro con normalidad". Espirar dándonos cuenta de lo que hacemos y pensar "Espiro con normalidad". Hacerlo tres veces. A la cuarta, alargar la inspiración, siendo conscientes de que "Espiro de forma prolongada". Hacerlo tres veces. Ahora, observemos la respiración con atención, atentos a cada movimiento del estómago y los pulmones. Observemos la entrada y salida del aire. Estemos atentos a cómo "Estoy inhalando y observando la inhalación desde el principio hasta el final. Estoy exhalando y observo la exhalación desde el principio hasta el final". Hacerlo durante 20 respiraciones. Volver a la normalidad. Después de 5 minutos, repetir el ejercicio. Mantener una media sonrisa mientras se respira. Una vez que dominemos este ejercicio, pasemos al siguiente.

g. Respirar para calmar la mente y el espíritu

Sentarse en el suelo con las piernas cruzadas (si sabemos hacerlo, nos sentaremos en la posición de loto o medio loto); o sentarse en una silla con las plantas de los pies tocando el suelo (también podemos arrodillarnos, tendernos en el suelo). Hacer media sonrisa. Observar la respiración.. Cuando cuerpo y mente se hallen relajados, sigamos inspirando y espirando muy suavemente; estemos atentos a la respiración y pensemos "Estoy inspirando y pacificando cuerpo y mente. Estoy espirando y pacificando cuerpo y mente." Hacerlo tres veces, intensificando el pensamiento "Estoy espirando mientras mi cuerpo y mi mente están en paz. Estoy espirando mientras mi cuerpo y mi mente están en paz. Estoy espirando mientras mi cuerpo y mi mente están en paz". Mantener este pensamiento de 5 a 30 minutos, según la habilidad que tengamos para hacerlo y el tiempo del que dispongamos. Tanto el inicio como el final de este ejercicio debe ser relajado y suave. Cuando queramos detenernos, démonos un masaje en los músculos de las piernas antes de volver a sentarnos en una posición normal. Esperar un momento antes de ponernos de pie.

2. La relajación

Cualquier método de relajación, en su inicio, puede no producir los efectos deseados, es necesario **practicar muchas vece**s, cuantas más mejor, personalizar las técnicas y lograr pequeños objetivos cada vez hasta alcanzar la meta final: obtener el estado de relajación en cualquier momento a nuestra voluntad, independientemente de las circunstancias que nos rodeen. Es similar a aprender a montar en bicicleta, al principio cuesta trabajo dominar la técnica, pero una vez que se aprende, se adquiere para siempre.

Consideraciones previas que debemos tener en cuenta para que el entrenamiento sea lo más eficaz y beneficioso posible:

1. Asegúrate que tendrás tranquilidad y silencio durante el tiempo que practiques.

2. Busca un momento en el que sepas que nadie te va a molestar, y practica sin prisa, para centrar toda tu atención en la relajación.

3. Intenta incorporar la relajación a tu vida diaria haciendo de su práctica una rutina más. Igual que tienes tu tiempo para comer, asearte, ver televisión, etc., también puedes buscar un tiempo cada día para practicar relajación.

4. Es conveniente practicar siempre a la misma hora y en la misma habitación, sobre todo al principio del entrenamiento. Una vez que vayas dominando las técnicas, puedes ir flexibilizando el horario y practicando en diversos lugares.

5. Ponte ropa cómoda y suelta, quítate gafas, relojes y zapatos para reducir, de este modo, estimulaciones extrañas y permitir la libertad de movimientos.

6. Adopta una postura cómoda donde la cabeza y el tronco estén en línea recta, y asegúrate de que la habitación esté bien ventilada y con temperatura adecuada.

7. Es conveniente dejar transcurrir unas dos horas después de comer, para que no interfiera con la digestión.

8. Adopta una actitud de abandono activo, dejándote llevar y no resistiéndote a las sensaciones corporales que pudiesen aparecer. Si durante la relajación te vienen a la cabeza pensamientos distractores, la mejor postura a adoptar ante ellos es una actitud de indiferencia. No te esfuerces en tratar de eliminarlos o de que no aparezcan, no te alarmes ante su presencia y sigue concentrándote en practicar la relajación.

9. Es importante que tengas a mano alguna manta o prenda de abrigo, ya que durante la relajación desciende la temperatura corporal y puedes sentir frío.

10. Empieza poco a poco, sin juzgarte ni enfadarte.

¿Cómo hacerlo?

La técnica de Jacobson se basa en tensar y relajar sistemáticamente varios grupos de músculos y aprendiendo a atender y a discriminar las sensaciones resultantes de la tensión y la relajación, se puede llegar a eliminar casi completamente las contracciones musculares y experimentar una sensación de relajación profunda. Te aconsejo no realizar todo el cuerpo en las primeras sesiones, prueba por partes para experimentar.

No te desanimes si al principio no te va todo lo bien que esperas, es normal, se trata de un aprendizaje. ¡Con la práctica lo conseguirás!

Aquí te dejo unos links a los que puedes acceder para tu práctica:

https://www.youtube.com/watch?v=m5DAzMq_ls0

https://www.youtube.com/watch?v=RbQ-_RZPPaE

3. La relajación mental para crear contextos imaginarios

Aquí te propongo hacer uso de tu imaginación para aprender a relajarte. En la relajación imaginativa aprendemos a interiorizar ciertas sensaciones: color, olor, frescor, calor, gusto, movimiento, etc. que se relacionan con distintos mundos imaginarios: un bosque, un viaje al fondo del mar, un vuelo entre las nubes, etc.

El contexto de referencia lo constituyen lugares y situaciones concretas en los que nos sentimos a gusto y en los que nos puede resultar fácil relajarnos Te describo a continuación algunos ejemplos para que puedas empezar a practicar, seguro que con el tiempo tú puedes crear los tuyos propios. Al final podrás escuchar unas grabaciones que te ayudarán a comenzar a practicar.

La habitación

Vamos a empezar con una relajación con la imaginación. Ponte en una posición cómoda, cierra los ojos, y escucha atentamente, imaginando todo aquello que te describo: Es de día, entras en una habitación rectangular y de color rosa. Las paredes son de un color rosa pálido. Cuando miras al suelo, ves que hay una gran alfombra de color marrón oscuro. Caminas hasta ella, la pisas y sientes la alfombra mullida bajo tus pies. No hay nada en la habitación excepto una mesa de madera en el medio. Sobre la mesa hay una jarra de cristal transparente llena de agua, junto a un vaso alto también transparente. Estás sediento y te alegras de ver el agua. Tu boca está seca. Caminas hacia la mesa, levantas la jarra con una mano y el vaso con la otra. Llenas el vaso de agua. Sientes un frescor muy intenso en la mano que sostiene el vaso a medida que éste se llena de agua. Vuelves a dejar la jarra en la mesa y aproximas el vaso hacia tus labios. El agua enfría tu boca y refresca todo tu cuerpo cuando tomas el primer trago. Te bebes todo el vaso de agua, que te refresca y a la vez te relaja. Realizas tres respiraciones profundas: 1…, 2… y 3… Te preparas para abandonar este ejercicio conservando todos los beneficios que te proporciona, contamos lentamente uno, dos y tres, abre cierra las manos, toma aire con más intensidad y abre los ojos.

Recuerda que éste es tu lugar especial para relajarte al que puedes venir siempre que lo desees.

La biblioteca misteriosa

Sentado en tu silla, con la espalda muy recta, respira tranquilamente. Cierra los ojos, concéntrate en el recorrido del aire desde el exterior, y cómo entra en tu interior y ve haciendo que tu respiración sea cada vez más profunda. Imagina que tu cuerpo es una gran casa, llena de habitaciones, una casa a la que adoras. Los pies, las piernas y los muslos son el sótano; las caderas, el vientre y la cintura, la planta baja; el estómago, el pecho y el tórax, el primer piso. La columna vertebral y la espalda, son las escaleras que unen todos los aposentos. Tus hombros, el cuello y la cabeza forman el último piso. Imagina ahora que en la parte más alta de tu cabeza se levanta una torre que alberga una extraña biblioteca, llena de hermosos libros. Imagina que estás ahí y que tienes un libro muy hermoso entre las manos: siente el tacto de piel de la portada, y la textura del papel; intenta recordar el olor de sus hojas impregnadas de tinta y de esas tapas de piel ya vieja. En esa torre, coincidiendo con el centro de tu frente, hay una gran ventana que se abre a un espacio infinito. Asómate a ella y contempla ese cielo limpio y esas nubes blancas y esponjosas durante unos segundos,…tienes una sensación de paz muy intensa, hacía tiempo que no te sentías tan bien. Realiza con mucha calma tres respiraciones profundas: 1…, 2… y 3… Te preparas para abandonar este ejercicio conservando todos los beneficios que te proporciona, contamos lentamente uno, dos y tres, abre cierra las manos toma aire con más intensidad y abre los ojos.

Recuerda que éste es tu lugar especial para relajarte al que puedes venir siempre que lo desees.

Relajación en el bosque

Vamos a empezar con una relajación con la imaginación. Ponte en una posición cómoda, cierra los ojos, y escucha atentamente, imaginando todo aquello que te describo. Concéntrate en el recorrido del aire desde el exterior, y cómo entra en tus pulmones y ves haciendo que tu respiración sea cada vez más profunda. Vas a comenzar visualizando un prado verde, de ese color verde que tanto te gusta, ves una extensión de césped verde que se extiende hasta un bosque cercano: observas la hierba verde, los reflejos dorados del sol sobre la pradera, y extiendes la mirada hacia el bosque antiguo que hay al fondo, y ves hojas de todos los tonos de verde posibles, lo cual es relajante, muy relajante…El sol se refleja en las verdes hojas de los árboles y se forma ante tus ojos una infinidad de delicadas tonalidades de verde que se reflejan entre sí creando un espacio tridimensional de color verde, es muy hermoso y lo disfrutas.... Respiras tranquilo y

en calma un aire puro, lleno del oxígeno que producen los árboles del bosque, te encuentras respirando ese aire puro, verde, relajante, y con cada respiración te relajas más y más… Escuchas el susurro que produce el aire al pasar entre las hojas de los árboles, es relajante, muy relajante..., escuchas ese sonido del viento en las hojas y te relajas más y más. Te quedas por unos instantes observando el prado verde, los viejos árboles centenarios, sus hojas hermosas y sintiendo tu respiración. Observa tu respiración, sin modificarla, tomas conciencia de que algo respira en ti, no te cuesta esfuerzo alguno respirar, respiras... y te llenas de calma, respiras... y te llenas de paz. Realiza tres respiraciones profundas: 1…, 2… y 3… Te preparas para abandonar este ejercicio conservando todos los beneficios que te ha proporcionado. Cuentas lentamente de nuevo uno, dos y tres... abre y cierra las manos, toma aire con más intensidad y abre los ojos.

Recuerda que éste es tu lugar especial para relajarte al que puedes venir siempre que lo desees.

4. Las preocupaciones excesivas

En la situación de crisis sanitaria mundial que estamos viviendo, es normal sentirse preocupado/a por diversos aspectos. Pero si te inundan las preocupaciones, intenta reservar media hora al día regularmente para preocuparte. Dedica un lugar y un tiempo que será “la hora de la preocupación” y cúmplelo. Si empiezas a preocuparte a otras horas, pospón la preocupación hasta “la hora de la preocupación” y vuelve a concentrarte en lo que estabas haciendo.

Durante “la hora de la preocupación” deja a tu mente preocuparse libremente. Coge un papel y un lápiz y escribe tus preocupaciones una a una.

Para ayudar a determinar si una preocupación se refiere a un problema basado en la realidad, puedes preguntarse lo siguiente:

- ¿Tengo alguna prueba real de que la preocupación es sobre un problema inmediato?
- ¿Tengo alguna prueba de que el problema sobre el que me preocupo aparecerá en un futuro próximo?
- ¿Me estoy preocupando por un problema que, de hecho, no existe en realidad?

Y a continuación, es importante determinar si se refiere a un problema modificable:

- ¿He resuelto alguna vez un problema como este?
- ¿Podría resolverlo actualmente?

Comparte tus preocupaciones

Las preocupaciones no sobreviven bien fuera de su mente. Hablar con alguien de ellas, nos puede ayudar a ampliar nuestra perspectiva de los problemas y a encontrar posibles soluciones. Trata de quedar con un amigo/a o familiar y dile lo que está pasando por tu mente. Todos/as tenemos preocupaciones: seguro que estas personas también tendrán cosas que les preocupan, por lo que no les será difícil comprenderte.

5. Controlando los pensamientos de irritabilidad

Para situaciones de intenso enfado, te puede ser útil intentar pensar de forma más equilibrada. Los procesos cognitivos a menudo distorsionan la realidad y producen más ira; debes estar atento para identificar y modificar estos pensamientos, hará disminuir tu ira y te dejará en una mejor posición para el afrontamiento, con más recursos. Un modo de conseguirlo es escribir dos columnas, una para los pensamientos que están fomentando tu irritabilidad y otra para los pensamientos más tranquilizadores. Por ejemplo:

- Pensamientos que fomentan irritabilidad: Es horrible, No puedo soportarlo, Es imbécil, Me va a volver loco, etc

- Pensamientos tranquilizadores: Esta situación es desagradable, Me siento mal pero se pasará, A veces se equivoca, Me hace enfadar.

Controlando los síntomas físicos de la irritabilidad

La relajación y los métodos de autocontrol pueden ayudar a reducir los sentimientos de la irritabilidad. Para reducir los síntomas físicos asociados a la irritabilidad es importante controlar la respiración. Si empieza a sentir ansiedad o estrés, pruebe a controlar su respiración, puede ser una buena manera de que se sienta más tranquilo/a. Nadie notará que está haciendo este ejercicio, por lo que puede realizarlo en cualquier lugar. Puedes visitar la información sobre la respiración de este módulo.

Controlando el comportamiento de la irritabilidad

Otra manera de reducir la irritabilidad sería controlando los comportamientos irritables que mostramos normalmente. Podemos hacerlo en tres fases:

1) Averigua cómo son tus comportamientos irritables - qué sucede antes y después de ellos. Puede ayudarte llevar un diario durante un periodo corto de tiempo. Anota durante un par de semanas cómo sueles comportarte en estas situaciones de enfado.

2) Haz una lista de comportamientos alternativos que puedes hacer cuando estás enfadado, y elige la opción que te parezca más adecuada para aplicarla en situaciones difíciles. Algunos ejemplos de comportamientos alternativos apropiados pueden ser:

• Pedir disculpas.

• Respirar profundamente y relajarse un segundo.

• Abandonar la situación de tensión unos momentos: salir a dar un paseo, cambiar de habitación…

• Intentar entender a la otra persona, pedirle una explicación. Buscar un momento para hablar de lo sucedido. El mejor momento para hablar las cosas no es cuando siento mayor irritación, esperaré a controlarme antes de conversar.

• Intentar adoptar el nuevo comportamiento en las situaciones de enfado. Escribe un diario sobre cómo fue, cuál fue la conducta alternativa que utilizaste y cómo te sentiste. Notarás cómo al cambiar tu forma de expresar la ira, también te SENTIRÁS distinto.

Recuerda: Para controlar los comportamientos de irritabilidad necesitas:

- saber cuáles son tus comportamientos de irritabilidad
- pensar cómo puedes comportarte de manera diferente
- intentar estas nuevas conductas alternativas

6. Recomendaciones para abordar el estrés

• **Darse cuenta.** Ser consciente de que el estrés está suponiendo un problema para ti constituye el primer paso para vencerlo.

• **Gestiona tu tiempo.** Planea tu tiempo, haz una cosa detrás de otra, no varias a la vez y realiza descansos regularmente. Alterna tareas aburridas con interesantes. Tómate vacaciones al menos una vez al año.

• **Ordena tus preocupaciones.** Divídelas entre las que puedes hacer algo al respecto (ya sea ahora o pronto) y las que no puedes hacer nada. No tiene sentido preocuparte por cosas que no puedes cambiar. VISITA EL MÓDULO “LUCHANDO CON LAS PREOCUPACIONES”

• **Cambiar lo posible.** Mira los problemas que puedes resolver, y pide toda la ayuda que sea necesaria para llevarlo a cabo. Aprende a decir “no”. Por ejemplo, si tu trabajo está generándote estrés piensa - ¿Cuál es el problema? ¿Cuáles son mis opciones? ¿Qué aspectos son estresantes?¿Podría mejorar en algo esos aspectos de mi trabajo?¿Podría delegar? ¿Podría obtener más apoyo?

• **No te exijas demasiado.** Trata de ponerte metas realistas.

• **Trata de concentrarte en el presente.** Trata de no insistir en preocupaciones pasadas o futuras.

• **Mira tus prioridades a largo plazo.** Mira hacia atrás y examina aquello que en tu vida te está provocando demasiado estrés. ¿Qué puedes desechar o cambiar? ¿Cómo puedes introducir un mayor equilibrio entre el trabajo, vida social y vida familiar? ¿Es hora de volver a evaluar tus prioridades?

• **Mejora tu estilo de vida.** Encuentra tiempo para comer adecuadamente (coma lentamente y sentado, dedíquele al menos una hora), haz ejercicio, duerme lo suficiente y evita el tabaco y el alcohol. VISITA EL MÓDULO DE ESTILOS DE VIDA SALUDABLES

• **Confía en alguien.** No mantengas las emociones reprimidas siempre, habla con tus amigos y cultiva su amistad. Concéntrate en los aspectos positivos de su vida. VISITA EL MÓDULO DE APOYO SOCIAL.

• **Aprende a relajarte.** Dedícale cada día un espacio de tiempo para la relajación y el ocio. Practica nuevas formas de relajación como el yoga o *taichi*. La relajación es una habilidad como cualquier otra que puede aprenderse con un poco de tiempo.

TODO ESTO PUEDE PARECERTE DIFÍCIL, AFRÓNTALO PASO A PASO ¡CON PRÁCTICA SEGURO QUE LO CONSIGUES!

7. ¿Cómo puedo regular mejor mi ansiedad?

Como hemos aprendido, la ansiedad no es una enfermedad y, por lo tanto, no se puede curar. Es una respuesta normal del organismo, incluso beneficiosa, pero que en algunas ocasiones puede resultar excesiva. Por tanto nuestro objetivo será aprender a regularla.

Para que sea más manejable, podemos trabajar en al menos cuatro áreas diferentes:

a. Entender mejor la ansiedad y empezar a abordar algunas de sus causas.
b. Reducir los síntomas físicos.
c. Modificar los pensamientos relacionados con la ansiedad.
d. Cambiar los comportamientos relacionados con la ansiedad.

a. Entender mejor la ansiedad.

• Haz un diario de ansiedad. Durante un período de dos semanas (o más si lo prefieres) mantén un diario de horas de ansiedad y nivel de actividad. Califícala de 0-10 (donde el 0 implicaría ninguna ansiedad y 10 el máximo grado). Anota todo lo que parezca importante. ¿Estabas en el trabajo o en casa?, ¿qué estabas haciendo?, ¿en qué estabas pensando? Podrás ser más consciente de las situaciones que te causan ansiedad o las que evitas. Esta información te ayudará a comenzar a hacerles frente.

• Estilo de vida estresante. La situación que estamos viviendo puede llegar a ser estresante y es fácil que existan presiones. No siempre podemos controlar el estrés que viene de fuera, pero podemos encontrar formas para reducir la presión que nos ponemos nosotros/as mismos/as: VISITA EL MÓDULO DE RECOMENDACIONES PARA ABORDAR EL ESTRÉS

b. Reducir los síntomas físicos de la ansiedad

• Relajación. Para reducir la intensidad de los síntomas físicos sería mejor “cortar el círculo vicioso de la ansiedad” al reconocer los primeros signos de tensión. Una vez que los has detectado, podrás prevenir la ansiedad con el uso de técnicas de relajación. Algunas personas pueden relajarse a través del ejercicio, escuchar música, ver televisión, leer un libro, acudir a clases de yoga, etc.

Para otras personas es más útil tener una serie de ejercicios a seguir. La relajación es una habilidad como cualquier otra que debe ser aprendida y requiere tiempo. VISITA EL MÓDULO DE RELAJACIÓN.

• La respiración controlada. Es muy común que las personas estén nerviosas por los cambios que se producen en su respiración. Puedes empezar a “tragar aire”, pensando que vas a asfixiarte o respirar muy rápido. Esto probablemente hará que sientas mareo y por lo tanto más ansiedad. Esto se llama exceso de respiración o hiperventilación. Trate de reconocer si estás haciendo esto y respira más despacio. A veces puede resultar útil respirar en una bolsa de plástico, cubriéndote la nariz y la boca. VISITA EL MÓDULO DE EJERCICIOS DE RESPIRACIÓN

• Distracción. Si distraes la mente de tus síntomas a menudo éstos desaparecen. Trata de mirar a tu alrededor. Estudia las cosas en detalle: qué tipo de decoración hay, qué ropa lleva la persona que está sentada a tu lado, etc. Necesitarás distraerte durante al menos tres minutos para que los síntomas empiecen a reducirse.

Si bien la relajación, los ejercicios de respiración y las técnicas de distracción pueden ayudar a reducir la ansiedad, es sumamente importante tener en cuenta que no es perjudicial o peligrosa, incluso si no usamos estas técnicas, no sucederá nada terrible. La ansiedad no puede hacernos daño, pero puede ser incómoda. Estas técnicas pueden ayudar a reducir el malestar.

c. Modificar los pensamientos relacionados con la ansiedad

Los pensamientos tienen un importante papel en el mantenimiento del círculo vicioso de la ansiedad. A veces también pueden aparecer imágenes en tu mente.

Por ejemplo, imagínate que estás en el hospital y, de repente notas cómo el corazón te late apresuradamente y sientes que te asfixias. El pensamiento que pasa por tu mente es: ”Me está dando un ataque al corazón”. Esto te puede generar mucho miedo, lo que hace que el latido cardíaco se acelere aún más y pienses que efectivamente se trata de un infarto, es probable que te imagines siendo atendido/a por tus compañeros de urgencias.

Pero no siempre es fácil saber lo que estás pensando y lo que está haciendo que tu ansiedad empeore.

Los pensamientos que experimentan las personas ansiosas pueden aparecer y desaparecer en un instante y pueden ser tan habituales que se producen automáticamente. Suelen ser tan familiares que puedes sentirlos como parte tuya.

Ahora, trata de imaginar la última vez en la que te sentiste muy nervioso/a. Trata de recrearte en ese recuerdo como si fuera una película, con el mayor detalle posible. Intenta escribir los pensamientos que experimentaste. Cualquier pensamiento puede ser importante. Ninguno es demasiado pequeño o demasiado tonto. Incluso “otra vez igual” o ”ya está pasando de nuevo” puede aumentar la tensión y la ansiedad. Algunos pensamientos son como imágenes desagradables en su mente. Trata de cambiar esos pensamientos negativos por otros más racionales y positivos (por ejemplo “en esta ocasión me he equivocado, pero no quiere decir que sea torpe”, “esta situación no será eterna, se trata de un mal trago temporal”).

d. Cambiar los comportamientos relacionados con la ansiedad

• Trata de reconocer cuando estás evitando las cosas (por ejemplo, posponiendo tareas importantes, pero necesarias).

• Siempre que sea posible trata de hacer frente a estos temores, no todos a la vez pero sí de una manera gradual.

• Establece metas pequeñas.

• Las personas a menudo establecen como hábito escapar de las situaciones que le generan ansiedad. En lugar de hacer esto aumenta gradualmente la duración de tu estancia en esa situación. La ansiedad a menudo llega a un punto álgido y, a continuación, comienza a desaparecer de forma natural. La gente suele pensar que cada vez irá a peor. Cuando compruebes que tras un periodo la intensidad de los síntomas empieza a descender, mejorará la confianza en tus posibilidades.

• Trata de hacer cosas para probar si tus pensamientos de ansiedad son realistas, por ejemplo, si piensas que yendo a una determinada zona del hospital te desmayarás, ves y comprueba lo que de hecho ocurre.

• Realmente es muy importante reconocer que **cuanto más se evita algo, más difícil será superarlo y cada vez te generará más ansiedad.**

8. Diez reglas para afrontar el miedo

Si en algún momento te bloqueas, aumenta tu miedo y piensas que puedes colapsar y entrar en pánico, sigue estas 10 recomendaciones para hacer frente al miedo (si es algo que te sucede con frecuencia, ten estas recomendaciones a mano para recurrir a ellas rápidamente).

1. Recuerda que las sensaciones que experimentas no son más que una exageración de las reacciones corporales normales al estrés (a la tensión).
2. No son, en absoluto, perjudiciales ni peligrosas. Sólo desagradables. No sucederá nada peor de lo que ya experimentas.
3. Deja de aumentar el pánico con pensamientos atemorizadores sobre lo que está sucediendo y dónde podría conducirte.
4. Observa lo que está sucediendo en tu cuerpo justo ahora, no lo que temes que pueda pasar.
5. Espera y deja tiempo a que el miedo pase. No luches en contra ni huyas. Simplemente acéptalo.
6. Observa que cuando dejas de aumentar el miedo añadiendo pensamientos atemorizadores, éste empieza a desaparecer por sí mismo.
7. Recuerda que el objetivo de la práctica se encuentra en aprender cómo hacer frente al miedo–sin evitarlo-. Por tanto, esta es una oportunidad para progresar.
8. Piensa en los avances que has conseguido hasta ahora, a pesar de todas las dificultades. Piensa en lo satisfecho que estarás cuando lo consigas, esta vez.
9. Cuando empieces a encontrarte mejor, mira al tu alrededor y empieza a planear qué harás después.
10. Cuando estés dispuesto a continuar, empieza de forma tranquila, relajada. No hay ninguna necesidad de esfuerzos ni de prisas.

9. Mejorando tu estado de ánimo

Con la difícil situación que estás viviendo, quizás tu estado de ánimo está más bajo de lo normal. Por eso, te queremos recomendar una serie de **estrategias que puedes poner en marcha para ayudarte a estar mejor.**

Estas estrategias no sustituyen la ayuda profesional, si te sientes mal no dudes en recurrir a los servicios de Salud Mental de tu hospital o zona.

1. Expresa tu sufrimiento y acepta la ayuda.

Intenta hablar aquellas cosas que te provocan sufrimiento con alguien próximo y explícale cómo te sientes. A menudo, repasar las experiencias dolorosas varias veces, llorar, y hablar de tus problemas con alguien es útil y es una forma natural que tiene nuestra mente para curarse. Además, resulta muy útil y beneficioso expresar, cuando estás mal, lo que sientes a las personas en las que confías. También es importante, una vez que aceptes la ayuda, que no te abrumes por sentimientos de baja autoestima o por el temor a que se te juzgue o no se te valore, porque esto podría llevarte a ocultar información importante. Además de estas relaciones importantes y estables, practica con frecuencia los “pequeños intercambios” (intercambia cada día unas palabras y una sonrisa con tus vecinos/as, comerciantes de tu barrio, compañeros de trabajo, el portero de tu vivienda o la administrativa de tu hospital...). Esto te permitirá sentirte mejor, reducir el aislamiento y sentirte más cómodo/a en tu entorno. En los momentos difíciles estos pequeños soportes emocionales son de gran valor.

2. Procura mantenerte activo.

Haz algo de ejercicio, aunque sea sólo pasear, te ayudará a mantenerte en forma y a dormir mejor. Puede que no te sientas capaz de trabajar, pero siempre es bueno intentar hacer alguna actividad: podrías hacer tareas domésticas, bricolaje (aunque sea tan poca cosa como cambiar una bombilla) o cualquier otra actividad de tu rutina normal. Esto te puede ayudar a distraerte de los pensamientos dolorosos que te hacen estar con menos ánimo. VISITA LOS MÓDULOS DE ESTILOS DE VIDA SALUDABLE

3. Procura realizar una dieta saludable.

Intenta llevar una dieta sana y equilibrada, incluso aunque no tengas ganas de comer. La fruta y las verduras frescas son especialmente buenas. Tu sufrimiento te puede hacer perder peso y vitaminas, lo que sólo serviría para empeorar tus problemas. VISITA LOS MÓDULOS DE ESTILOS DE VIDA SALUDABLE

4. Evita el consumo excesivo de alcohol y de otras sustancias tóxicas.

La angustia mental que experimentas podría favorecer el consumo de alcohol, ya que en principio puede dar la impresión de que alivia, de que te ayuda a distanciarse de tus problemas, de que tiene un efecto tranquilizante o calmante. Sin embargo, estos efectos inmediatos son una trampa: la impresión de mejora se disipa rápidamente. El alcohol tiene efectos depresores (disminución de las funciones cerebrales, fatiga, dificultad de concentración, tristeza) que están relacionados con la interferencia en el funcionamiento de varios neurotransmisores. En otros casos, el sufrimiento también puede conducir a consumir otras sustancias (exceso de fármacos ansiolíticos, tabaco, cannabis, cocaína, anfetaminas...). Como el alcohol, estas sustancias son tóxicas y tienen directa o indirectamente efectos depresores que seguramente agravarán la situación. Si tienes algún problema con los tóxicos, te recomendamos que busques ayuda profesional de tu zona. VISITA LOS MÓDULOS DE ESTILOS DE VIDA SALUDABLE

5. Intenta mantener una actitud positiva.

Recuerda que estás sufriendo una experiencia que mucha otra gente también está pasando, que al final saldrás de ella, aunque ahora te sea difícil creerlo. Quizás esta experiencia te haga más fuerte y con más capacidad para sobrellevar los problemas. Te puede ayudar a ver más claramente las situaciones y las relaciones, a ser más capaz de tomar decisiones importantes y a hacer cambios en tu vida, cambiando tu escala de valores. En todas las caídas resurgimos con más fuerza, aunque ello signifique pasar por una mala racha o experiencia y aceptar ese sufrimiento siendo amables con nosotros mismos.

MÓDULO 1.4. HABILIDADES DE SUPERVIVENCIA A LAS CRISIS EMOCIONALES

En este módulo de Habilidades de supervivencia encontrarás muchas estrategias relativas a qué puedes hacer cuando estás en situación de bloqueo o crisis emocional. ¡Elige la que más se ajuste a ti!
En la situación que estás viviendo son normales y no temas si en alguna ocasión te sientes perdido o no sabes qué hacer, te aseguro que este bloqueo pasará, es temporal.
¡Saldrás de esta!

Habilidades de supervivencia a las crisis emocionales

En la actual situación que ha generado el COVID-19, es normal que en algún momento puedan aparecer crisis emocionales. Todos experimentamos crisis personales. Las crisis son eventos agudos y de tiempo limitado que experimentamos como reacciones emocionales abrumadoras ante la percepción y como reacción a un evento.

Debido a la gravedad de los efectos de la pandemia que estamos viviendo, tus estrategias normales de afrontamiento pueden no ser efectivas momentáneamente. Y puede que lo primero que se te ocurra sean estrategias no saludables para escapar del dolor que estás sintiendo.

Te queremos ofrecer un conjunto de estrategias que van dirigidas a aprender a tolerar el dolor de una situación de crisis sin recurrir a conductas que mantengan o aumenten el sufrimiento, ¡elige las que mejor se adapten a ti!

1. Distraerte con diferentes estrategias

• Llevar a cabo actividades distractoras: Practicar una afición, limpiar, ver algún espectáculo por Internet, llamar o visitar a un amigo, pasear un rato, practicar un deporte, tomarse un café o un té descafeinado, arreglar el jardín, centrarse en alguna actividad de bricolaje o manualidad, leer un libro.

• Ayudar a los demás: Ayudar a alguien (un vecino que no puede salir), hacer algún trabajo voluntario, dar algo a alguien, hacer algo hermoso por alguien, preparar una sorpresa.

• Comparar nuestra situación con otras: Compararse con gente que está en la misma situación que nosotros o peor. Compararse con gente menos afortunada.

• Generar emociones opuestas a la que estamos sintiendo: Leer libros, cartas o cuentos que provoquen las emociones opuestas; o ver películas que nos animen; escuchar música a un alto volumen, que nos motive (asegurémonos de que el evento crea en nosotros diferentes emociones.) Ideas: películas de miedo, libros cómicos, comedias, discos divertidos, música divertida, etc

• Dejar de lado situaciones negativas (no significa que tengas que evitar pensar en ella, sólo cuando estás en crisis): Dejar de lado la situación durante un rato a nivel mental. Construir un muro imaginario entre uno mismo y la situación. O hacerlo bloqueando la situación en la mente. Rechazar pensar en los aspectos negativos de la situación. Poner el dolor en un estante, encerrarlo en una caja y dejarlo ahí durante un rato.

• Tener pensamientos distractores: Contar hasta 10 (o más si hace falta); centrarte en contar los colores de un cuadro, de las ventanas, de cualquier cosa; hacer rompecabezas o mandalas; ver la televisión; leer.

• Experimentar sensaciones intensas: Sostener hielo en la mano; apretar una bola de goma con la mano; ducharse con agua muy caliente; escuchar música a volumen alto; practicar sexo; darse un masaje muy reconfortante.

2. Proporcionarse estímulos positivos. Se trata de relajar cada uno de los cinco sentidos:

- Vista: Comprar una flor hermosa, arreglar y decorar de manera especial un espacio de la casa. Encender una vela y mirar la llama. Poner la mesa con esmero para una comida concreta. Ir a un museo de arte hermoso (o mirarlo por Internet). Contemplar la naturaleza, como un científico que lo ve por primera vez. Salir a la terraza durante la noche a contemplar las estrellas. Caminar por una zona hermosa de la ciudad. Mirar unas fotos hermosas en un libro. Ser consciente de lo que vemos al caminar, sin detenernos en nada en especial.
- Oído: Escuchar una música hermosa y tranquilizadora (o excitante y animada). Prestar atención a los sonidos de la naturaleza (olas, pájaros, lluvia y hojas). Cantar una canción agradable. Tararear una melodía agradable. Aprender a tocar un instrumento. Llamar a un teléfono de información o similar para oír una voz humana. Ser consciente de cualquier sonido que aparezca y dejar que entre por un oído y salga por el otro.
- Olfato: Usar el perfume o las lociones favoritas o probarlas en diferentes partes del cuerpo. Darte un masaje con crema aromática. Ambientar la casa con una fragancia agradable. Encender una vela aromática. Poner aceite al limón en los muebles. Poner un cuenco de flores aromáticas en la habitación. Hervir canela ; cocinar galletas, un pastel o pan. Oler rosas. Caminar por una zona arbolada y respirar conscientemente los frescos olores de la naturaleza.
- Gusto: Cocinar una buena comida; tomar la bebida preferida (sin alcohol), como té de hierbas o chocolate caliente. Autoinvitarse a un postre. Ponerse crema de leche en el café (descafeinado). Probar diferentes sabores en una heladería. Tomarse un caramelo, muy despacito. Mascar nuestro chicle favorito. Comprar un poco de esa bebida o comida en la que normalmente no nos gastamos mucho dinero, como zumo de naranja natural. Probar y disfrutar con todo detalle el sabor de la comida. Comer algo con plena conciencia.
- Tacto: Darse un baño de espuma; poner sábanas limpias en la cama y disfrutar de ellas. Acariciar al perro o al gato. Que nos den un masaje. Mojar los pies en algún lugar. Ponerse loción o crema lentamente en el cuerpo. Ponerse una compresa fría en la frente. Sentarse en una silla realmente

cómoda en casa. Ponerse una blusa, un vestido o una bufanda de seda. Ponerse unos guantes o un chaquetón de piel. Cepillarse el pelo durante un rato. Abrazar a alguien. Acariciar a nuestro animal de compañía. Experimentar con detalle cualquier cosa que toquemos; notar el tacto suave de algo.

3. Mejorar el momento actual, a través de nuestra mente:

- Imaginación: Imaginar escenas muy relajantes. Imaginar que tenemos una sala en nuestro interior y contemplar cómo está decorada. Entrar en esa sala siempre que nos encontremos amenazados. Cerrar la puerta de esa sala siempre que nos sintamos amenazados. Imaginar que todo va bien. Imaginar que resolvemos las cosas muy bien. Inventar un mundo de fantasía calmado y hermoso y dejar que la mente entre en él. Imaginar que las emociones dañinas se agotan como agua de una fuente.
- Hallar significado: Encontrar (o crear) algún propósito, significado o valor al dolor. Recordar, escuchar o leer acerca de valores espirituales. Centrarse en los aspectos positivos de las situaciones dolorosas que podemos encontrar. Repetirlos una y otra vez en la mente. Hacer de la necesidad virtud.
- Si eres creyente, practicar la oración: Abrir el corazón al ser supremo, a una sabiduría superior, a Dios, a nuestra propia mente sabia.
- Pedir ayuda para superar el dolor del momento. Levantar nuestras plegarias a Dios o a un ser superior.
- Practicar la relajación (VISITA EL MÓDULO DE RESPIRACIÓN Y RELAJACIÓN): Intentar la relajación muscular tensando y relajando cada grupo muscular, empezando por las manos y los brazos, siguiendo hacia la parte superior de la cabeza y después recorriendo el resto del cuerpo. Escuchar una cinta de relajación; hacer mucho ejercicio; tomar un baño caliente; darse un masaje en el cuello y en el cuero cabelludo o en las pantorrillas y pies. Darse un baño con agua muy caliente o fría y quedarse allí hasta que el agua esté tibia. Respirar profundamente; hacer una media sonrisa; cambiar la expresión facial.
- Vivir el presente en cada momento (VISITA EL MÓDULO DE MINDFULNESS). Centrar toda la atención en lo que estamos haciendo. Quedarse en el preciso momento en el que estamos; situar la mente en el presente. Centrar la atención en las sensaciones físicas que acompañan a las tareas no mentales (por ejemplo, caminar, hacer la colada, lavar los platos, limpiar, arreglar algo). Darse cuenta de cómo se mueve el cuerpo durante una tarea. Hacer ejercicios de conciencia.
- Tomarse unas breves vacaciones: Buscar el momento para concederse unas vacaciones. Meterse en la cama y cubrirse la cabeza durante 20 minutos. Pedir a nuestro compañero de piso que nos traiga el café a la cama o que nos prepare la cena (ofrecerse para hacer lo recíproco en otro momento). Comprarse una revista del corazón, meterse en la cama con chocolatinas y pasarse la tarde leyéndola. Preparase una merienda que nos apetezca, sentarse en una silla cómoda y saborearla durante un buen rato. Llevarse una manta a la terraza y pasar un rato sentado allí. Descolgar el teléfono durante todo un día o conectar el contestador automático para no tener que responder. Darse un respiro de una hora tras haber realizado un duro trabajo.
- Darse ánimo: Animarse a sí mismo. Repetir una y otra vez: "Puedo superarlo", "No durará para siempre", "Saldré de ésta", "Estoy haciendo lo mejor que puedo".

4. Pensar en los pros y contras: Hacer una lista de los pros y contras de tolerar el malestar. Hacer otra lista de los pros y contras de no tolerar el malestar: esto es, de abusar del tabaco o el alcohol (o de las drogas) o hacer algo impulsivo. Centrarse en los objetivos a largo plazo, la luz al final del túnel. Recordar las ocasiones en que el dolor ha finalizado. Pensar en las consecuencias positivas de tolerar el malestar. Imaginar cuán bien nos sentiremos cuando consigamos nuestros objetivos. Darse cuenta de que para ello no hemos de actuar impulsivamente. Pensar en todas las consecuencias negativas de no tolerar el malestar actual. Recordar lo que ha sucedido en el pasado cuando actuamos compulsivamente para escapar al momento.

MÓDULO 2.1. ESTILOS DE VIDA SALUDABLE

Este módulo tiene como objetivo recordarnos que los estilos de vida están relacionados con nuestro bienestar mental y que, en situaciones de presión o estrés, como es el caso de la pandemia actual, es especialmente importante seguir un estilo de vida saludable. Encontrarás información relativa a diferentes factores de nuestro estilo de vida, así como estrategias y habilidades específicas, que te irán ayudando a modificar aquello que necesites para un estilo de vida más saludable ¡ánimo con ello!

1. Actividad física y exposición solar

Mantenernos activos en nuestra vida cotidiana y la exposición a la luz del día tienen una estrecha relación con nuestro estado de ánimo. Además, cierto tipo de ejercicio físico, como salir a caminar o un trote suave, mejorarán de forma inmediata nuestro bienestar emocional. Cómo el tiempo es limitado te sugerimos que compatibilices **la exposición solar** con el ejercicio físico, es decir que hagas ejercicio en el exterior y durante el día.

- En general te recomendamos un mínimo de 30 minutos de ejercicio moderado-intenso 5 días a la semana (ej. caminando a paso ligero sin poder mirar escaparates). Y recuerda, cuantos más días mejor.
- Sabemos que puede ser difícil conseguirlo, pero intenta sacar al menos 10 minutos 3 veces al día. Puedes empezar paulatinamente e ir progresando.
- Recuerda que puedes incorporar la actividad física en tu vida. Sube por la escalera en lugar de usar el ascensor, cambia tu forma de desplazarte, siempre que puedas ve caminando o en bici en vez de coche, bájate del autobús una parada antes... Intenta reducir los periodos sedentarios de más de 2 horas seguidas, levantándote y dando un paseo corto o haciendo estiramientos.
- El mejor momento para hacer ejercicio es a primera hora del día, no obstante, elige el momento que mejor te venga.
- Y no olvides que mejorar la movilidad de tus articulaciones es importante, recuerda realizar actividades de flexibilidad.

Además, es importante que identifiques qué barreras te impiden realizar ejercicio físico. En caso de que aún no las utilices, te animamos a instalar en tu móvil alguna aplicación (como Google Fit) que te permita programar y registrar tu actividad física semanal e incluso recordarte si te has olvidado realizarla ya que lo que sabemos sobre nosotros puede ayudarnos a mejorar.

- ¡Ahora es tu turno! Encuentra tu motivo y da el primer paso (enlace a vídeo motivador sobre actividad física: https://www.youtube.com/watch?v=wYwHIkpQmqQ)
- ¿Eres una persona activa? Comprueba en 5 minutos si haces suficiente actividad física: https://estilosdevidasaludable.sanidad.gob.es/actividadFisica/descubrelo/home.htm

Enlaces:

Google Fit: https://www.google.com/fit/

Runkeeper (monitorización actividad física):

Android: https://play.google.com/store/apps/details?id=com.fitnesskeeper.runkeeper.pro&hl=es_419

iOS: https://apps.apple.com/es/app/runkeeper-registro-por-gps/id300235330

Yoga gratis para principiantes:

Android: https://play.google.com/store/apps/details?id=com.downdogapp.intro

iOS: https://apps.apple.com/es/app/yoga-down-dog/id983693694

Fitbit Coach:

Android: https://play.google.com/store/apps/details?id=com.fitstar.pt

iOS: https://apps.apple.com/es/app/fitbit-coach/id535640259

Ejercicios caseros:

Android: https://play.google.com/store/apps/details?id=com.ejercicioscaseros

iOS: https://apps.apple.com/us/app/ejercicios-caseros/id1321412738?l=es

Entrenamiento de 7 minutos:

Android: https://play.google.com/store/apps/details?id=com.popularapp.sevenmins

iOS: https://apps.apple.com/us/app/7-minute-workout-fitness/id1014299720

Ten en cuenta que nuestra motivación variará en el tiempo, y que, aunque en ocasiones nos parece difícil adoptar estos estilos de vida saludables, no significa que hayamos fracasado o que no podamos remontar la situación.

2. Alimentación saludable

Todos conocemos la importancia de una dieta equilibrada tanto para nuestra salud física como mental, aunque en ocasiones es difícil poder mantenerla de forma regular. A veces, cuando llegas a casa después de un agotador día de trabajo, con muchas situaciones complicadas, quizá lo que más nos apetece es optar por alimentos fáciles de preparar, o precocinados, pero sabemos que no es lo más adecuado para nosotros.

Gestionar la culpa

Comer de vez en cuando aquello que no es tan adecuado para nosotros no es preocupante y normalmente lo aceptamos. Sin embargo, cuando aparece la culpa después de comerlo es porque de alguna manera sabemos que lo que estamos haciendo no es bueno para nosotros mismos. Lo que pasó pasado está, pero en este momento párate a pensar en otra opción de comida más saludable pero igualmente satisfactoria que no alimente la culpa. Esto te ayudará a no añadir preocupaciones a las que ya tienes y además evitarás emociones desagradables en el momento de la comida, un momento que está muy presente en nuestras vidas, pues comemos más de 3 veces cada día.

Comer equilibrado

Habrás oído este concepto millones de veces, pero a la hora de la verdad a todos nos cuesta reconocer qué es comer equilibrado y sano al mismo tiempo. Por eso, **aquí te dejamos unas pautas para ponerlo en práctica (sigue las que puedas, poco a poco irás incorporando las demás):**

- Asegura una ración de verduras frescas o cocinadas en cada comida y en cada cena como por ejemplo una ensalada, judía verdes con zanahoria, tortilla de espinacas, salteado de champiñones…
- Consume 3 piezas de fruta al día, por ejemplo en el desayuno, media mañana y de postre en la comida o en la cena.
- Come legumbres al menos 3 veces por semana.
- Cambia la pasta, el arroz y el pan por las opciones integrales.
- Incluye el pescado, la carne y los huevos en 2-3 ocasiones por semana cada uno.
- No te olvides de un puñado de frutos secos cada día, por ejemplo en la merienda de la tarde.
- Usa el aceite de oliva virgen extra como grasa principal para cocinar y aliñar.
- La mejor bebida siempre es el agua.
- Evita el consumo de alimentos precocinados, *fast-food*, carnes procesadas, sopas solubles, snacks, bollería y bebidas azucaradas.
- Cuanta menos sal, mejor.

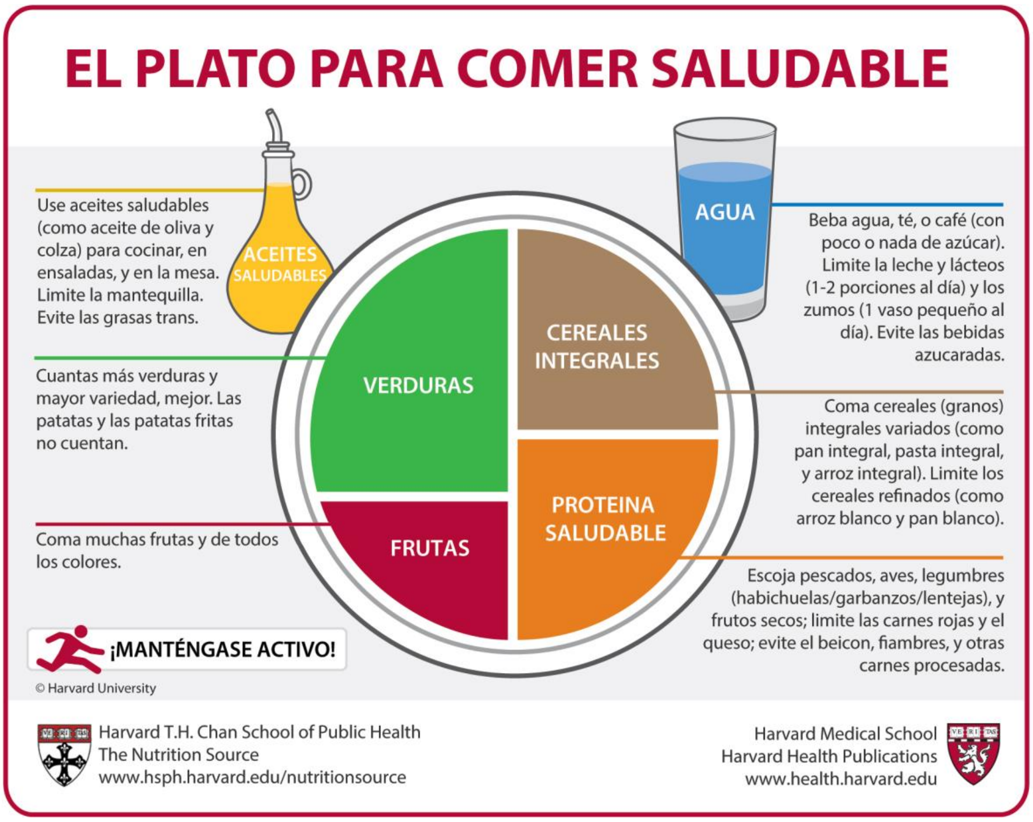

- ¿Cómo es mi alimentación? https://encuestas.msssi.gob.es/limesurvey/index.php/814367

Comer con atención

También debemos tener en cuenta que comer estando distraídos (mirando televisión, jugando con el móvil, navegando por internet o leyendo) hace que comamos más y durante más tiempo sin darnos cuenta. Además, somos menos conscientes de los tipos de alimentos que ingerimos. Degustar lo que nos llevamos a la boca, apreciando las sensaciones sensoriales que produce sin darle tampoco una importancia excesiva a la potencia del sabor y centrándonos más en otras cualidades como la textura, la cremosidad, la frescura de un mordisco de lechuga o el jugo de un tomate al romperlo entre tus dientes. Experimenta el placer de sentir la comida y el acto de comer en su totalidad. Presta atención a los sonidos, los colores y la satisfacción de llevarte algo al estómago. Valora la compañía de las personas que te acompañan o de la voz en la radio o la televisión si estás solo/a. Entiende el acto de comer como algo más allá del simple sabor salado o dulce e interiorízalo como un proceso de autocuidado y placer para tu cuerpo y tu mente simultáneamente.

Ejemplos prácticos para comer sano y equilibrado sin complicarte la vida

- Ensalada de garbanzos, lentejas o alubias
- Hervido de verduras, patata y huevo duro
- Cuscús salteado con verduras y tofu
- Pasta integral con mejillones y sofrito de tomate, pimiento y cebolla
- Guiso de bacalao/merluza con sofrito, brócoli y patata
- Hamburguesa de pollo con calabacín, pimiento rojo, aguacate, brócoli, cebolla y champiñones
- Arroz con pollo, calabaza y espárragos
- Verduras salteadas con alubias
- Tortilla de berenjenas con ensalada de tomate y pepino
- Salmón a la plancha con judías verdes y zanahoria
- Quinoa con tiras de ternera, pimiento rojo, cebolla y calabacín

Más recursos prácticos:

Tu menú saludable: https://www.tumenusaludable.com/descargas/

Fundación Dieta Mediterránea: https://dietamediterranea.com/

Ten en cuenta que nuestra motivación variará en el tiempo, y que, aunque en ocasiones nos parece difícil adoptar estos estilos de vida saludables, no significa que hayamos fracasado o que no podamos remontar la situación.

3. Consumo de alcohol y tabaco

La situación actual nos obliga a modificar nuestros hábitos y rutinas, lo que nos puede afectar emocionalmente y puede provocar un aumento en el consumo de alcohol y tabaco, que como profesional sanitario, ya sabes que puede ser perjudicial para tu salud.

En relación al alcohol:

- Existen muchos mitos como por ejemplo que el consumo de alcohol relaja y ayuda a aliviar el estrés, pero la realidad es muy diferente, ya que su consumo puede generar estrés y ansiedad, alterar el sueño, interferir en nuestras relaciones y en la capacidad de afrontamiento.

- ¿Tengo que preocuparme de mi consumo de alcohol?: https://encuestas.msssi.gob.es/limesurvey/index.php/768276?lang=es

En relación al tabaco (y derivados):

El tabaquismo sigue siendo la primera causa evitable de enfermedad y muerte prematura en España. Seguro que conoces los riesgos que el tabaco puede tener para nuestra salud, pero también existen falsos mitos (https://estilosdevidasaludable.sanidad.gob.es/tabaco/falsosMitos/home.htm). ¡No importa la edad que tengamos, ya que, si dejamos de fumar encontraremos un montón de beneficios para nuestra salud y la de nuestros familiares y amigos, así como para nuestra economía! Si te interesa conocer tu consumo de tabaco, puedes accede al siguiente cuestionario: https://encuestas.msssi.gob.es/limesurvey/index.php/464684?lang=es.

Hay 3 aspectos fundamentales a los que tendrás que enfrentarte si quieres dejar de fumar:

1. El poder de adicción de la nicotina, que actúa en nuestro cerebro y es la responsable de la dependencia física.

2. La relación psicológica con el tabaco, ya que su consumo está íntimamente ligado con nuestras emociones (tristeza, alegría, estrés, etc.) y nuestros gestos y rutinas más habituales.

3. El aspecto social, ya que su consumo acompaña en muchas ocasiones nuestras relaciones sociales y el entorno influye.

- Recuerda que fumar es una adicción que implica múltiples riesgos para la salud, por tanto, es todo lo contrario de ayudar o controlar nada.
- Debes tener en cuenta que la nicotina es un estimulante y su falta genera ansiedad en los fumadores. Al dejar de fumar eliminarás esa ansiedad y serás más libre.
- Intenta cambiar tus rutinas para romper con el consumo de tabaco. Si fumas al levantarte, intenta realizar ejercicio físico a primera hora. Ponte en marcha rápidamente despúes de comer y evita la sobremesa o lávate los dientes. Los cigarrillos no son el postre.

Aquí te dejamos unos enlaces interesantes que pueden ayudarte a liberarte del tabaco:

Vídeo campaña antismoking: https://www.youtube.com/watch?v=aHrdy6qcumg&feature=youtu.be

Guía para dejar de fumar:
https://www.mscbs.gob.es/ciudadanos/proteccionSalud/adolescencia/docs/guiaTabaco.pdf

S’Acabó:

Android: https://play.google.com/store/apps/details?id=com.mcvendrell.sintabaco

iOS: https://apps.apple.com/es/app/sacabo/id956633440

Respirapp:

Android: https://play.google.com/store/apps/details?id=com.kodens.respirapp

iOS: https://apps.apple.com/es/app/respirapp/id1356204710

Ten en cuenta que nuestra motivación variará en el tiempo, y que, aunque en ocasiones nos parece difícil adoptar estos estilos de vida saludables, no significa que hayamos fracasado o que no podamos remontar la situación.

4. Sueño

Invertimos un tercio de nuestra vida en dormir, ya que es una actividad necesaria para restablecer el equilibrio físico y psicológico básico. En momentos como los que estamos actualmente viviendo, esto es aún más necesario si cabe. La necesidad de sueño cambia en cada persona, de acuerdo con la edad, el estado de salud, el estado emocional y otros factores. Por ejemplo, el sueño de las personas mayores se hace más ligero (suelen pasar más tiempo en las fases 1-2) y son más frecuentes los despertares nocturnos, porque influyen los cambios de vida (por ejemplo, jubilación), la mayor incidencia de problemas de salud y el mayor uso de medicación. Por tanto, el tiempo ideal de sueño es aquel que nos permita realizar las actividades diarias con normalidad.

Recuerda que dormir adecuadamente es imprescindible para equilibrio físico y psíquico de las personas. Cuando dormimos el cuerpo produce melatonina y serotonina, hormonas que contrarrestan los efectos del estrés. Posiblemente tus ciclos de sueño pueden estar alterados, por el intenso trabajo al que estás sometido, las guardias, los cambios horarios, etc.

Se considera que alguien tiene **insomnio** cuando los problemas de calidad y/o cantidad con el sueño sobrepasan una intensidad que los hace intolerables y cuando hay dificultades para adaptarse a las consecuencias negativas que derivan de estos problemas, provocando síntomas como alteraciones del estado de ánimo, dificultad para concentrarse, somnolencia diurna, cansancio físico y mental, etc. Las **manifestaciones** más características del insomnio son las dificultades para conciliar o mantener el sueño, acompañadas de una falta de sueño reparador (que tiene lugar durante el denominado sueño profundo o de ondas lentas, en las fases 3-4).

Para evitar alteraciones del sueño es recomendable seguir los siguientes consejos:

- Utiliza la cama sólo para dormir y tener relaciones sexuales. No debes dormir con luz ni utilizar dispositivos electrónicos (TV, móvil, tablet), puesto que la glándula pineal reacciona a la luz y en consecuencia no permite la segregación de melatonina (hormona del sueño), y el sistema de alerta se activa de forma que el inicio del sueño se retrasa. Tampoco te recomendamos trabajar en la cama ni justo antes de dormir.
- Mantén la habitación oscura, silenciosa y a temperatura agradable (entre 19 y 22º).
- La ropa para dormir tiene que ser ligera o inexistente.
- Mantén un horario y una rutina de sueño cada día de la semana, ayuda a evitar alterar el ciclo interno de sueño y vigilia. Intenta irte a dormir y despertarte a la misma hora (en la medida de lo posible).
- Realiza actividades relajantes en la hora previa de irte a dormir (un baño, tomar una infusión relajante, meditar…). VISITA LOS MÓDULOS DE RELAJACIÓN Y MINDFULNESS
- Olvida los problemas por la noche (no pienses en ellos, date un respiro). Mañana será otro día. Si hay algo que te ronda en la cabeza y no puedes dormir, anótalo en un papel y ya lo retomarás mañana.
- Intenta cenar al menos dos horas antes de irte a dormir. Mejor si la cena es ligera, sin alimentos ni bebidas estimulantes como cacao, café, té o refrescos con cafeína.

- No tomes bebidas alcohólicas antes de irte a dormir, ya que disminuyen la calidad del sueño produciendo interrupciones del sueño, pesadillas y dolor de cabeza matinal.
- Se desaconseja fumar y mucho menos antes de ir a dormir.
- No picotees durante la noche si te despiertas, porque puede constituir un refuerzo positivo para los despertares nocturnos, haciendo que te despiertes más noches.
- Si realizas siestas que sean cortas (20 minutos).
- La actividad física ayuda a dormir mejor, pero no se recomienda hacer ejercicio físico justo antes de ir a dormir, ya que actúa como excitante.
- No te esfuerces o empeñes en dormir, dormir es un acto pasivo. Si no puedes dormir no le des demasiada importancia, levántate de la cama y realiza alguna actividad relajante hasta que tengas sueño (VISITA LOS MÓDULOS DE RELAJACIÓN Y RESPIRACIÓN).

Enlaces:

App Sleep Time para controlar los ciclos de sueño: https://www.azumio.com/s/sleeptime/index.html

Ten en cuenta que nuestra motivación variará en el tiempo, y que, aunque en ocasiones nos parece difícil adoptar estos estilos de vida saludables, no significa que hayamos fracasado o que no podamos remontar la situación.

MÓDULO 2.2. ESTILOS DE VIDA SALUDABLE. EJERCICIOS PRÁCTICOS

Este es un módulo más práctico. Encontrarás unas recomendaciones que te ayudarán a sentirte mejor.

Aunque nuestros patrones de conducta en general constituyen hábitos que hemos ido adquiriendo a lo largo de nuestra vida, podemos cambiarlos y mejorarlos para cuidar nuestra salud. La práctica de ejercicio físico de forma regular, una dieta saludable, y una adecuada higiene del sueño favorecen nuestro bienestar global, tanto emocional como físico.

En ocasiones, cuando nos sentimos estresados o ansiosos bajamos la guardia y no le damos suficiente importancia a estos estilos de vida y nos alimentamos mal, abusamos de la comida procesada, tendemos al sedentarismo (exceso de TV o uso de tablets o móviles), no nos exponemos adecuadamente a la luz solar y se deterioran nuestros hábitos de sueño. Con todo ello podemos entrar en una espiral que se retroalimenta y hará que nuestro malestar psicológico se pueda agravar. Por ello, es tan importante identificar las conductas saludables como reconocer qué excusas y obstáculos nos impiden adoptar un estilo de vida más saludable.

Por tanto, queremos incidir en la importancia de recordar que los estilos de vida saludables constituyen un factor esencial en la prevención de complicaciones derivadas del estrés al que hemos estado sometidos durante esta crisis sanitaria.

1. Ideas prácticas ejercicio físico.

En estos momentos quizá te está costando más realizar la actividad física que sueles hacer, pero puedes ser más activo/a siguiendo estas recomendaciones:

1. Empieza. Si no te mueves mucho, empezar es lo importante. Algo de actividad física es mejor que nada. Sea cual sea tu estado de salud, sólo por empezar a moverte ya obtienes beneficios. Mantente activo/a hasta donde te sea posible y, sobre todo, hazlo de manera progresiva.
2. Encuentra tu motivo. Piensa en qué razones te pueden animar a empezar. Repasa los beneficios de ser una persona más activa y da el primer paso.
3. Adáptala a tu vida, que quizá ahora es muy diferente a la que tenías antes de la pandemia. Incorpora la actividad física a tu rutina diaria, yendo a hacer recados andando o subiendo por las escaleras en lugar de usar el ascensor. Intenta no estar más de 2 horas seguidas sentado/a, realizando períodos activos o “pausas saludables” cada 1 o 2 horas con sesiones cortas de estiramientos o dando un breve paseo. Limita el tiempo delante de una pantalla (televisión, ordenador, teléfono móvil, tabletas, consolas de videojuegos, etc.).
4. Muévete más y genera movimiento a tu alrededor. A veces, puede resultar más fácil motivarte si realizas actividad física con otras personas. Anima a las personas que conviven contigo. ¡Así conseguirás además que otras personas sean más activas!

5. Cambia tu forma de desplazarte. Siempre que puedas, ve caminando o en bicicleta, en vez de en coche. ¡Además de cuidar de tu salud, cuidarás del medio ambiente!
6. Infórmate y explora los recursos y actividades de tu zona. Parques, rutas para caminar o ir en bici, paseos organizados, polideportivos, centros para mayores, centros culturales, clubes deportivos y asociaciones, federaciones deportivas o cualquier otra actividad de ocio.

Para más información:

Consulta la página web del Ministerio de Sanidad, Consumo y Bienestar Social: www.estilosdevidasaludable.mscbd.gob.es.

Si necesitas más información o apoyo para ser una persona más activa, en tu centro de salud podrán ayudarte. También podrás encontrar qué recursos hay disponibles en tu localidad en: https://localizasalud.mscbs.es/maparecursos/main/Menu.action.

Enlaces:

Google Fit: https://www.google.com/fit/

Runkeeper (monitorización actividad física):

Android: https://play.google.com/store/apps/details?id=com.fitnesskeeper.runkeeper.pro&hl=es_419

iOS: https://apps.apple.com/es/app/runkeeper-registro-por-gps/id300235330

Yoga gratis para principiantes:

Android: https://play.google.com/store/apps/details?id=com.downdogapp.intro

iOS: https://apps.apple.com/es/app/yoga-down-dog/id983693694

Fitbit Coach:

Android: https://play.google.com/store/apps/details?id=com.fitstar.pt

iOS: https://apps.apple.com/es/app/fitbit-coach/id535640259

Ejercicios caseros:

Android: https://play.google.com/store/apps/details?id=com.ejercicioscaseros

iOS: https://apps.apple.com/us/app/ejercicios-caseros/id1321412738?l=es

Entrenamiento de 7 minutos:

Android: https://play.google.com/store/apps/details?id=com.popularapp.sevenmins

iOS: https://apps.apple.com/us/app/7-minute-workout-fitness/id1014299720

2. Ideas prácticas exposición a la luz solar.

¿Cómo afecta la luz solar a nuestro estado de ánimo y a nuestra salud?

¡La luz solar es clave para nuestro estado de ánimo! Y llevamos desde mediados de marzo sin poder hacer nuestra vida normal. La mayoría hemos estado confinados, o también en cuarentena. Con largas jornadas laborales y con restricciones de movimiento. La cantidad predominante de luz solar afecta a la actividad de la serotonina cerebral, una sustancia que actúa como hormona en diferentes partes del cuerpo (por ejemplo, regulando la digestión o la temperatura corporal) y como neurotransmisor en nuestro cerebro (participando, principalmente, en la regulación de los estados de ánimo, pero también del apetito, el deseo sexual y el sueño, entre otros).

La exposición a la luz solar, a diferencia de la luz azul emitida por las pantallas de móviles, ordenadores y otros dispositivos electrónicos, es fundamental para mantener un ritmo regular del ciclo sueño-vigilia, ya que es el principal sincronizador de nuestro sistema circadiano y es necesaria para que se pueda sintetizar melatonina durante la noche para favorecer un descanso saludable.

Te recomendamos que encuentres 10-15 minutos al día para exponerte al Sol, de manera cuidadosa, protegiéndote bien y evitando quemaduras solares.

3. Ideas prácticas alcohol y tabaco

A menor consumo de alcohol, menor riesgo

Si no bebes nada, continúa así, porque es la opción más segura.

Si bebes a diario, pero no superas los máximos recomendados, proponte **días libres de alcohol** (es decir, intenta no beber nada de alcohol al menos 1 o 2 días a la semana).

Si bebes ocasionalmente, pero de forma intensiva, aunque no superes los máximos recomendados, intenta reducir tu consumo. ¿Cómo?

1. Elige bebidas alternativas a las alcohólicas.
2. No bebas alcohol para calmar la sed ni mientras practicas deporte, ya que tiene un efecto diurético.
3. Bebe con calma, procurando disfrutar del sabor y no de los efectos del alcohol, y mejor con el estómago lleno.
4. Fíjate un límite razonable de consumo y lleva la cuenta de lo que bebes.
5. No bebas por beber, sáltate alguna ronda.

Si quieres dejar de fumar, estas son algunas **recomendaciones**:

1. Sólo es posible conseguirlo si llegas al convencimiento de que es un reto que puedes alcanzar. ¡Evita que las dudas te paralicen, aunque lo hayas intentado en otras ocasiones y hayas recaído, no renuncies a conseguirlo!
2. Fija una fecha para comenzar, analizando previamente los pros y los contras de ese día y, sobre todo, evitando épocas con un exceso de estrés en tu vida. ¡Aunque no utilices el estrés como excusa para aplazar una decisión tan beneficiosa para la salud!
3. Prepara un plan, infórmate sobre las dificultades de las primeras semanas (la principal es el denominado síndrome de abstinencia) e intenta anticiparte a ellas, preparando el mayor número de alternativas posibles.
4. Ante la duda, busca apoyo en tu centro de salud, amigos, familiares u otros recursos, tantas veces como necesites.

4. Ideas prácticas dieta saludable.

En esta situación que estamos viviendo, sabemos que puede ser más difícil llevar una dieta saludable, por eso aquí te dejamos unas ideas prácticas y links que esperamos puedan ayudarte:

1. Diseña un menú semanal. Al hacerlo, presta atención tanto al tipo de alimentos como a las cantidades recomendables y cómo las distribuyes. Haz la lista de la compra a partir del menú. Lee en las etiquetas la composición nutricional. Existen algunas aplicaciones móviles de nutrición que

pueden resultarte útiles: https://www.midietacojea.com/2019/11/28/probamos-las-apps-de-nutricion-mas-conocidas-del-mercado-el-futuro-es-apasionantevodafone-y-el-pais/.

2. Cocina con poca sal. Tu paladar se irá acostumbrando y saborearás mejor los alimentos. Las especias pueden ser un buen sustituto y ayudan a mejorar el sabor de tus platos. Elige, siempre que puedas, aceite de oliva, tanto para aliñar en crudo (ensaladas, marinados) como para cocinar (al vapor, hervido, cocido, guisado, asado, horneado, frito). Si reutilizas el aceite, ten en cuenta que su calidad empeora.
3. Los desayunos son un buen momento para incluir una pieza de fruta. Mira cuantas opciones tienes de hacerlo: https://nortesalud.com/producto/preparate-un-desayuno-saludable/ y https://nortesalud.com/producto/ideas-de-desayuno-saludable/.
4. Comer sencillo y poco elaborado puede ser igualmente saludable. Consulta el menú tipo que te proponemos y cocina los ingredientes sugeridos como más te gusten. Por ejemplo, puedes preparar un cocido de garbanzos con verduras y carne o saltear los mismos garbanzos con verduras y carne en la sartén. El primer ejemplo tarda más de 1 hora en prepararse, el segundo solo te llevará 15 minutos si utilizas legumbres previamente cocidas. Ajusta tu menú saludable a tus necesidades.

Ensalada<br>Legumbres	Verdura<br>Pescado<br>Patata	Ensalada<br>Remolacha<br>Carne roja	Verdura<br>Legumbres	Ensalada<br>Pescado<br>Pan	Verdura<br>Legumbres	Ensalada<br>Marisco<br>Arroz integral
Verdura<br>Huevos<br>Pan	Ensalada<br>Pasta<br>Atún	Verdura<br>Guisantes<br>Huevos	Ensalada Cuscús<br>Carne blanca	Verdura<br>Patata<br>Huevos	Ensalada<br>Quinoa<br>Queso	Verdura<br>Pescado<br>Patata

5. Durante la comida, come despacio, masticando bien los alimentos. No uses el salero en la mesa y recuerda que la mejor bebida es el agua. Come sólo hasta saciarte, no termines con la sensación de haber comido de más. Si es posible, come acompañado, ya que es una buena manera de relajarse y estrechar relaciones sociales. Si te interesa aprender a comer de manera consciente, te recomendamos este libro (http://www.siglantana.com/editorial/mindful-eating-el-sabor-de-la-atencion/) y estos audios: https://www.webmindfulness.com/recursosaudioseating/.

Para más información

Consulta la página web del Ministerio de Sanidad, Consumo y Bienestar Social: www.estilosdevidasaludable.mscbd.gob.es. Encontrarás información acerca de los diferentes tipos de alimentos, podrás poner a prueba lo que sabes, descargar tablas con información útil sobre los alimentos, tablas de planificación de menús y aprender habilidades de compra y cocinado. Además, podrás conocer mejor cómo te alimentas y qué aspectos podrías mejorar.

También podrás encontrar qué recursos hay disponibles en tu localidad en: https://localizasalud.mscbs.es/maparecursos/main/Menu.action.

Otros recursos

Canada's Food Guide (en español): https://www.canada.ca/en/health-canada/services/canada-food-guide/resources/snapshot/languages/spanish-espagnol.html

Food and Nutrition Service (Spanish Materials Available from Team Nutrition): https://www.fns.usda.gov/tn/espanol

García, M. (2019). El jamón de York no existe: La guía para comprar saludable y descubrir los secretos del supermercado. Madrid: La esfera de los libros.

Ríos, C. (2019). Come comida real. Barcelona: Ediciones Paidós.

Sánchez, A. (2016). Mi dieta cojea. Barcelona: Ediciones Paidós.

Sánchez, A. (2018). Mi dieta ya no cojea. Barcelona: Ediciones Paidós.

Web de Carlos Ríos: https://realfooding.com

Web de Aitor Sánchez: https://www.midietacojea.com

MÓDULO 3.1. ESTRÉS LABORAL

En este módulo encontrarás información relativa al concepto de **estrés laboral.** Esta situación de pandemia que estamos viviendo tiene una gran capacidad de generar estrés laboral en muchos de nosotros. Te aconsejamos que visites este módulo varias veces y sigas sus recomendaciones, ¡pueden serte de mucha ayuda!

ESTRÉS LABORAL

En la actual situación de pandemia generada por el COVID-19, seguramente muchos de nosotros viviremos situaciones complicadas, que posiblemente nos generen un estrés laboral (debido a la incertidumbre de la situación, el exceso de trabajo, la falta de medios para hacer frente a la pandemia, entre otros), por ello te ofrecemos aquí información acerca de este concepto.

¿Qué es el estrés laboral?

El estrés es un desequilibrio sustancial entre las demandas de la situación y nuestra capacidad de afrontarlas.

Ocurre sobre todo si creemos que no afrontar las demandas de la situación tendrá importantes consecuencias para otros o para nosotros

Este desequilibrio se acompaña de reacciones fisiológico-psicológicas (malestar, irritabilidad, falta de concentración, insomnio, etc).

Los principales estresores laborales son:

- La sobrecarga de trabajo
- Tener un trabajo muy difícil (incertidumbre sobre la enfermedad y cura)
- Relaciones con los demás (pacientes, familiares)
- Los riesgos de infección
- La falta de recursos (materiales, humanos, etc)

¿Cómo afrontar el estrés?

- Deja de correr y párate a pensar un momento en lo que estás viviendo. Te ayudará a coger fuerzas.
- Identifica estresores. Cuando tengas un momento, ¿piensa qué es lo que más te está estresando de todo lo que te rodea?
- ¿Puedes cambiar esa fuente de estrés? Intenta cambiar la situación, reorganiza el trabajo, habla con tus compañeros/as, con tu supervisor. Busca ayuda.
- ¿No puedes cambiar el estresor? Seguramente en estos momentos no depende de ti, prueba a tomártelo de otra manera, a mirar las cosas con otros ojos porque eso sí puedes cambiarlo.

- Prioriza. Muchas veces nos estresa tener demasiadas cosas que hacer o pendientes. Haz las más importantes primero, luego tendrás tiempo para el resto. Y hazte listas, si está anotado en un papel no te rondará la cabeza y dormirás mejor.
- Tómate un respiro. Haz pausas durante tu turno para descansar, estirarte o hablar con colegas, compañeros de trabajo, amigos y familiares que puedan brindarte apoyo.
- Cuídate. Intenta mantener conductas de salud (dormir, ejercicio físico, comer bien) para recargar pilas y tener más fuerzas para el día siguiente
- Intenta relajarte. Dedícate unos minutos para relajarte o hacer respiraciones profundas. Te dará fuerzas.
- Apóyate en tus seres queridos. Habla o llama a tu familia y amigos e intenta reírte con ellos o desahogarte. Te sentirás mejor.
- Apóyate en tus compañeros/as. Comparte tu estrés laboral con ellos y buscad juntos información o estrategias para llevar mejor la situación de estrés.
- Intenta mantener una perspectiva positiva. No hay mal que 100 años dure y entre todos conseguiremos resolver la situación.
- Aprovecha o busca oportunidades para mejorar la situación. Expresa tus opiniones o sugerencias sobre cómo organizar el trabajo, obtener medios, resolver problemas.
- No estés siempre conectado. Tómate descansos y deja de mirar, leer o escuchar las noticias. Esto incluye redes sociales. Escuchar hablar de la pandemia todo el tiempo puede ser desagradable y mentalmente agotador, especialmente porque trabajas con personas afectadas directamente por el virus.

MÓDULO 3.2. *BURNOUT*

El *burnout* o ‘estar quemado’ es muy frecuente cuando vivimos situaciones muy estresantes y muy intensas. Sobre todo si nuestro trabajo es ‘intentar ayudar a los demás’ y tenemos poco recursos.

Los tres síntomas principales de *burnout* son:

- *Agotamiento emocional.* Sentimientos de cansancio físico o/y psicológico. La sensación de que ‘se te han acabado las pilas’, que tienes la sensibilidad a flor de piel y que te resulta difícil sentir o gestionar tus emociones.

- *Despersonalización.* Dejas de ver a tus pacientes como personas y empiezas a verlos como un número. Si es muy acusada, puedes desarrollar actitudes negativas, hostiles, y distantes hacia los pacientes y sus familias.

- *Baja realización personal.* Por todo lo anterior te sientes un mal profesional, y padeces una pérdida de autoestima tanto personal como profesional. Lo peor es que sientes que no cumples con tus expectativas.

La **fatiga por compasión** es la pérdida de la sensación de satisfacción que resulta de hacer bien el trabajo, o el estrés relacionado con el trabajo que supera las satisfacciones laborales.

Si has llegado a este punto o ves que vas por este camino, NECESITAS AYUDA.

La ayuda más efectiva si es posible, es desahogarse con grupos de ayuda de tu centro sanitario o con compañeros que han vivido situaciones similares a la tuya. Reír, llorar, quejarse, y comentar juntos la situación vivida te permitirá:

- desahogo emocional (hablar de lo que has vivido con compañeros o profesionales psicólogos/psiquiatras te ayudará a expresar lo que has vivido y elaborarlo mejor)
- darte cuenta de que no estás sólo y de que otros han vivido la situación igual que tú,

- aprender de las estrategias positivas que otros utilizan para llevar mejor la situación e intentar copiarlas.

Además, puedes recurrir a las prácticas de mindfulness para la prevención del estrés y el *burnout*.
VISITA PARA ELLO EL MÓDULO DE MINDFULNESS

En casos graves, te aconsejamos que valores solicitar ayuda psicológica profesional para su tratamiento.

En este vídeo encontrarás una guía de consejos que te ayudarán a superar el estrés laboral:
https://www.youtube.com/watch?v=W7I_jtzVB6s

MÓDULO 4.1. APOYO SOCIAL

La importancia del entorno. Aprender a relacionarse con los demás

Este módulo tiene como objetivo recordarnos que el apoyo social está relacionado con nuestro bienestar mental y que, en situaciones de alta presión o estrés, como es el caso de la pandemia actual, es especialmente importante seguir manteniendo un buen nivel de apoyo social. Encontrarás información relativa a los diferentes tipos de apoyo social existentes, ¡encuentra el que más te puede ayudar!

Ante una situación de estrés como la generada por el COVID-19 es importante buscar apoyo social en las personas que nos rodean. **Existen cuatro tipos de apoyo social:**

i) Apoyo emocional. Implica tener a alguien que nos escuche de forma activa y abierta, alguien que percibas que se preocupa por ti. No consiste en recibir consejo o instrucciones de alguien, sino de encontrar a una persona que te ofrezca un hombro sobre el que llorar.

Estrategia positiva: ¿Qué podría ayudar a alguien a superar una situación de crisis emocional? Una buena estrategia sería buscar un compañero o compañera para explicarle cómo te sientes. Tú harías lo mismo ¿verdad?

ii) Apoyo informativo. Implica recibir información útil o fuentes de donde puedes extraerla para poder desempeñar tu tarea con éxito.

Estrategia positiva: Si tienes una cuestión que te preocupa, puedes consultar a tu supervisor/a, a un compañero/a de servicio con el que tengas confianza o a grupos de especialistas sobre cómo resolver esa cuestión.

iii) Apoyo instrumental. Se trata del apoyo práctico, "de hacer", cuando alguien de tu entorno realiza una acción práctica para ayudarte a conseguir tus objetivos.

Estrategia positiva: Si no puedes con la situación, ¿por qué no pedir a alguien que te cubra una hora para poder irte antes? También puedes pedir a alguien que te ayude a realizar un trabajo difícil.

iv) Apoyo valorativo. Es el apoyo que te pueden ofrecer tus compañeros y compañeras para evaluar situaciones de conflicto concretas.

Estrategia positiva: Hablar con otra persona y tener en cuenta su opinión puede ayudarte a considerar otra perspectiva o encontrar nuevas soluciones.

MÓDULO 4.2. RECURSOS Y HABILIDADES DE APOYO SOCIAL

En este módulo te aconsejamos visitar 2 recursos web. En el primer recurso encontrarás un vídeo que te ayudará a entender rápidamente qué es el apoyo social. En el segundo podrás profundizar en el porqué de la importancia de la conexión y el apoyo social, con aspectos clave relacionados, como la empatía, la compasión, la salud, etc.

Además, te proporcionamos una serie de consejos, estrategias o habilidades para conseguir apoyo social y para que los puedas integrar en tu código de conducta social.

Recursos

1. El apoyo social: aspectos conceptuales (@ Juan María Prieto - Universidad de Valladolid)

https://www.youtube.com/watch?v=Qv1S4xtoh2g

Estas son las **principales preguntas** a las que se da respuesta en este video divulgativo, y que te ayudarán a entender rápidamente qué es el apoyo social:

- ¿Qué es el apoyo social?
- ¿Qué tipos de apoyo social existe?
- ¿Por qué es importante el apoyo social?
- ¿Cuáles son los distintos niveles de apoyo social que podemos encontrar?

2. El poder y la ciencia de la conexión social (Título original: THE POWER & SCIENCE OF SOCIAL CONNECTION) (@ TEDx Talks) (video en inglés, con subtítulos en castellano -activar en barra de herramientas) (duración aproximada: 14 minutos)

https://www.youtube.com/watch?v=WZvUppaDfNs

Estas son las **principales preguntas** a las que se da respuesta en este video divulgativo, y que te ayudarán a entender de manera muy didáctica el porqué de la importancia de la conexión y el apoyo social:

- ¿Cuántas personas cercanas suele tener la gente para compartir con ellas un problema personal?
- ¿Qué explica la ciencia sobre los efectos de la conexión social?
- ¿Cómo podemos mejorar en nuestra vida la conexión social?
- ¿Qué efectos tiene en la persona una conexión social baja o nula?
- ¿Qué actividades se asocian a la salud? ¿Se asocia a la salud el concepto de conexión social?
- ¿Qué relación hay entre la empatía y la conexión social?
- ¿Qué relación hay entre la compasión y la conexión social?

Consejos, estrategias y habilidades

Las personas a menudo realizamos cambios en nuestra red de apoyos sociales cuando experimentamos importantes cambios en nuestras vidas, en situaciones de presión o estrés, como es el caso de la pandemia actual, o en otras situaciones más habituales: padres primerizos, divorcio o muerte de cónyuge, querer evitar hábitos no saludables (fumar, beber,…), nuevos pasatiempos o actividades, necesidad de conocimiento especializado u opinión experta, etc.

¿Qué puedo hacer para conseguir el apoyo social necesario que me ayude en esta nueva situación de mi vida?

- Haz crecer tu red de apoyo social:
    - Busca personas en las que puedas confiar, para diferentes tipos de apoyo. Una sola persona no sirve para ayudarte en cualquier tipo de necesidad de apoyo. Hay que diferenciar cuatro tipos de apoyo social: emocional (p.ej. tener a alguien al que poder explicarle cómo nos sentimos), formativo (p.ej. acudir a un grupo de especialistas para resolver un problema concreto), instrumental (p.ej. cuando alguien hace algo que te ayuda a conseguir tu objetivo, como cubrirte una hora en el trabajo) y valorativo (p.ej. hablar con una persona y tener en cuenta su punto de vista). Diferentes personas te pueden ayudar con cada una de estas necesidades. Puedes volver a consultar en el módulo 4.1 las diferencias entre estos cuatro tipos de apoyo social.

- Sé proactivo. No esperes a que otros se acerquen a ti sin extender antes tu mano; debes hacer un esfuerzo para estar disponible para otros, y probablemente ellos lo estarán para ti.

- Aprovecha las tecnologías de redes sociales. Aunque es más agradable sentarse con un amigo o una amiga cara a cara, no siempre es posible; un correo electrónico, un mensaje de texto, o una videollamada pueden servirte para encontrar el desahogo necesario y recibir palabras de consuelo.

- Aprovecha tus intereses y pasatiempos. Es más probable que conectes con personas que coinciden con tus aficiones e intereses; únete a un club, inscríbete en una clase, haz voluntariado, etc.

- Mejora tus habilidades sociales. Ante situaciones de incomodidad o timidez en ambiente social, o quizás porque ahora no estás en tu mejor momento, puedes probar por hacer preguntas simples sobre la otra persona, para que ruede fácilmente la conversación, o romper el hielo planteando preguntas relacionadas con actividades o pasatiempos habituales entre las personas; en última instancia, puedes formarte en habilidades sociales a través de un terapeuta experto en el tema.

- Pide ayuda. Si no estás seguro o segura de por dónde empezar a ampliar tu red social, siempre hay recursos a los que puedes recurrir: los centros de actividades para adultos, lugares de culto, o bibliotecas locales pueden ayudarte a localizar servicios, grupos de apoyo y otros programas de tu zona. Ahora puede que estén un poco limitados, pero poco a poco podremos acceder a más recursos sociales de este tipo.

● Cuida tu red social actual: es más probable que recibas apoyo de tu red social actual si cuidas esas relaciones; devuelve llamadas y mensajes de texto, involúcrate en videollamadas, ofrece ayuda a otros cuando lo necesiten, y hazles saber que los aprecias, pide ayuda también tú si lo necesitas. Y siempre mantén una comunicación asertiva y de respeto.

● Evita las relaciones negativas: las relaciones negativas son perjudiciales para la salud emocional; evita las personas que abusan de poder, aquellas con negatividad constante en sus interacciones, las que son excesivamente dependientes de otros, y aquellas que tienen problemas de control de conducta. Tú no eres responsable de la conducta de otra persona, pero sí que puede afectar o empeorar tu propio bienestar. Por eso, en situaciones de conflicto personal, evita buscar ayuda en este tipo de relaciones.

● Intégrate en un grupo de apoyo: si necesitas apoyo para un problema muy específico, como manejar un problema de salud físico o mental, un grupo de apoyo formal puede ser la mejor opción; en este sentido, puede ser de gran ayuda acudir a personas que se enfrentan a desafíos similares. Quizá en tu hospital o servicio el hablar con compañeros que están pasando por lo mismo que tú puede ayudarte. Si no existen estos grupos de apoyo social, tal vez puedas iniciar o liderar la formación de un grupo de apoyo en tu centro de trabajo.

● Expresa con claridad tus necesidades de apoyo social: no se debe suponer que otras personas de tu entorno han detectado tus conflictos personales y que saben lo que necesitas; al contrario, debes informar de ello y ser muy específico y claro en tu solicitud de ayuda, las personas que te aprecian seguro que quieren saber cuándo algo no va bien.

● No tengas miedo de asumir riesgos sociales: puedes crear nuevas oportunidades para conocer a otros cuando vas más allá de tus actividades habituales; para ello, es recomendable asistir a reuniones informales a las que hayas sido invitado, realizar cursos recreativos, integrarte en puestos de voluntariado, etc. Pide a tus amistades que te presenten a nuevas personas.
