# Supplementary material for: A Mobile Phone–Based Intervention to Reduce Mental Health Problems in Health Care Workers During the COVID-19 Pandemic (PsyCovidApp): Randomized Controlled Trial
Source: JMIR Mhealth Uhealth. 2021 May 18;9(5):e27039. doi: 10.2196/27039 (PMC8133164; doi:10.2196/27039)
Supplement: Multimedia Appendix 2 [file mhealth_v9i5e27039_app2.pdf]

## Multimedia Appendix 2. Content of the Control App

### MÓDULO 1. RETOS A LOS QUE SE ENFRENTA EL PERSONAL SANITARIO

Los brotes epidémicos, como el actual coronavirus (COVID-19), exigen una especial atención a las necesidades de apoyo emocional del personal sanitario, que podría verse sometido a situaciones de incertidumbre y estrés ante las dificultades en la atención médica.

Cuidarse a sí mismo y animar a otros a autocuidarse mantiene la capacidad de cuidar a los pacientes.

Los retos a los que se enfrenta el personal sanitario durante esta crisis del coronavirus son:

1.- Desbordamiento en la demanda asistencial

2.- El riesgo de infección no se detiene

3.- Equipos insuficientes e incómodos

4.- Proporcionar apoyo y atención sanitaria

5. - Gran estrés en las zonas de atención directa

6.- Exposición al desconsuelo de las familias

7.- Dilemas éticos y morales

**1.- Desbordamiento en la demanda asistencial**
En circunstancias como la del COVID-19 muchas personas acuden a los servicios sanitarios reclamando atención médica, al tiempo que el personal sanitario también se contagia o ve a sus personas cercanas enfermar, debiendo guardar cuarentena o precisar ellos mismos atención médica.

**2.- El riesgo de infección no se detiene**
Se trata de una situación de mucha incertidumbre ante el riesgo de contraer enfermedades temidas y transmitirlas a familiares, amigos y otras personas en el trabajo.

**3.- Equipos insuficientes e incómodos**
El equipo de protección individual puede dar una sensación de seguridad incierta, puede ser insuficiente, poco confortable, limita la movilidad y la comunicación.

**4.- Proporcionar apoyo y atención sanitaria**
Para el personal sanitario puede ser cada vez más difícil manejar la situación según aumenta la demanda y la asistencia, así como la angustia del paciente y las familias.

**5. - Gran estrés en las zonas de atención directa**
Ante situaciones de crisis y emergencias, el trabajo sanitario puede resultar gratificante, pero también puede ser una fuente de miedo, pena, frustración, culpa, insomnio y agotamiento.
Se trata de reacciones esperables en estas situaciones. Cuidarse en estos escenarios implica comprender que son reacciones normales ante situaciones anormales.

**6.- Exposición al desconsuelo de las familias**
El personal sanitario se expone a situaciones de sufrimiento intenso motivadas por el aislamiento de pacientes y el desconsuelo de las familias por no poder acompañar y ayudar a sus seres queridos en sus últimos momentos.

**7.- Dilemas éticos y morales**
La crisis del COVID-19 obliga al personal sanitario a tomar decisiones complejas en un breve tiempo, y todo ello en un contexto de falta de medios, sobrecarga y una evolución incierta de los pacientes, generando intensos dilemas morales y culpa.

## MÓDULO 2. REACCIONES EN SITUACIONES DE ESTRÉS INTENSO

Reacciones posibles en situaciones de estrés intenso: ante situaciones de estrés intenso como las que se han vivido durante esta crisis, es posible experimentar distintos tipos de reacciones.

Podemos clasificarlas en 4 grupos:

- Reacciones emocionales
- Reacciones conductuales
- Reacciones cognitivas
- Reacciones físicas

Emocionales

- Ansiedad
- Impotencia
- Frustración
- Miedo
- Culpa
- Irritabilidad
- Tristeza
- Anestesia emocional

Conductuales

- Hiperactividad
- Aislamiento
- Evitación de situaciones de personas o de conflictos
- Verborrea
- Llanto incontrolado
- Dificultad para el autocuidado y descansar/ desconectarse del trabajo

Cognitivas

- Confusión o pensamientos contradictorios
- Dificultades de concentración para pensar de forma clara o para tomar decisiones
- Dificultades de memoria
- Pensamientos obsesivos y dudas
- Pesadillas
- Imágenes intrusivas
- Fatiga por compasión
- Negación
- Sensación de irrealidad

Físicas

- Dificultades respiratorias: presión en el pecho, hiperventilación…
- Sudoración excesiva
- Temblores
- Cefaleas
- Mareos
- Molestias gastrointestinales
- Contracturas musculares
- Taquicardias
- Parestesias
- Agotamiento físico

- Insomnio
- Alteraciones del apetito

Conocer estas posibles reacciones te ayudará a identificarlas en caso de que las experimentes.

En el módulo M3. encontrarás algunas recomendaciones que pueden ayudarte a lidiar con esta situación.

## MÓDULO 3. RECOMENDACIONES PARA CUIDAR TU SALUD MENTAL

### Consejos para mejorar el bienestar de los y las profesionales sanitarios

**El cuidado de las necesidades básicas.**
En situaciones de crisis sanitaria, como profesionales de salud solemos pensar que siempre hay que estar disponibles para otras personas y que nuestras necesidades personales básicas son secundarias. Es importante que las cuides, asegúrate de comer, beber y dormir regularmente. También es importante descansar.

**Planificar rutinas fuera del trabajo.**
Es importante tratar de mantener los hábitos que ya van permitiendo las medidas de restricción.

**El contacto con compañeros/as.**
Es importante no dejar de lado las amistades y relaciones que ya tenías.

**Respetar a las diferencias.**
Cada persona tiene unas necesidades diferentes al experimentar sentimientos de ansiedad o estrés. Algunas necesitan hablar mientras que otras necesitan estar solas. Trata de reconocer y respetar estas diferencias.

**Compartir información constructiva.**
Todas las personas de un equipo nos complementamos: los elogios pueden ser motivadores poderosos y reductores del estrés.

**Estar en contacto con la familia y seres queridos.**
Ponte en contacto con tus seres queridos, si es posible. Esas personas son el sostén fuera del sistema de salud, por lo que es importante compartir y mantenerse conectado.

**Actualizarse.**
Es importante actualizar conocimientos sobre la pandemia, confiando y acudiendo a fuentes fiables de conocimiento. Participa en reuniones para mantenerte informado de la situación, planes y eventos, limitando la exposición a los medios de comunicación

**Permitirse pedir ayuda.**
Una de las maneras de regulación interna es reconocer signos de estrés, pedir ayuda y aprender a pararse para atenderlos. De esta manera se favorece la estabilidad frente a una situación de estrés mantenido en el tiempo.

**Auto-observación: tomar conciencia de las emociones y sensaciones.**
Ante una situación de peligro, la mente reacciona y se defiende con emociones desagradables. No se trata de una amenaza, es una reacción normal. Sin embargo, se debe vigilar a lo largo del tiempo para detectar cualquier síntoma de depresión o de ansiedad.

**Recordar que lo que es posible no es probable.**
Es importante no perder la esperanza y recordar también que una parte importante de las personas enfermas padecen este virus en otras formas más leves.

**Reconocer al equipo asistencial.**

Reconozcamos a nuestros colegas, ya sea formal o informalmente. Hay que recordar que todos los que en estas circunstancias están trabajando en hospitales, hagan lo que hagan, son los auténticos héroes para la población general.
