# Supplementary material for: A Mobile Phone–Based Intervention to Reduce Mental Health Problems in Health Care Workers During the COVID-19 Pandemic (PsyCovidApp): Randomized Controlled Trial
Source: JMIR Mhealth Uhealth. 2021 May 18;9(5):e27039. doi: 10.2196/27039 (PMC8133164; doi:10.2196/27039)
Supplement: Multimedia Appendix 3 [file mhealth_v9i5e27039_app3.pdf]

Multimedia Appendix 3. Subgroup analyses: comparison of outcome measures between the PsyCovidApp and the Control App groups at 2 weeks in prespecified subgroups (high DASS-21 baseline scores; users of psychotherapy; consumers of psychotropic medications)

Table S5.1: DASS-21 baseline scores subgroups (high and low scores, with threshold based on median DASS-21 score)

	Sample of completers at follow-up (n=203)			Overall sample b (n=231)			Interaction [c]
	Estimated mean difference (95% CI) [a]	Standardized group difference (95% CI) [a]	*P* value	Estimated mean difference (95% CI) [a]	Standardized group difference (95% CI) [a]	*P* value	*P* value
DASS-21 overall score							.20
High baseline DASS-21 overall score	-1.49 (-4.05 to 1.08)	-0.08 (-0.20 to 0.06)	.13	-1.42 (-4.02 to 1.17)	-0.07 (-0.20 to 0.063)	.15	
Low baseline DASS-21 overall score	-0.10 (-1.81 to 1.61)	-0.01 (-0.12 to 0.11)	.46	-0.11 (-1.80 to 1.59)	-0.01 (-0.12 to 0.11)	.45	
DASS-21- depression subscale							.35
High baseline DASS-21 overall score	-0.11 (-1.06 to 0.84)	-0.01 (-0.14 to 0.11)	.41	-0.01 (-1.06 to 0.96)	-0.01 (-0.13 to 0.11)	.43	
Low baseline DASS-21 overall score	0.14 (-0.43 to 0.71)	0.30 (-0.94 to 0.16)	.32	0.12 (-0.45 to 0.70)	0.03 (-0.10 to 0.15)	.33	
DASS-21- anxiety subscale							.07
High baseline DASS-21 overall score	-0.69 (-1.57 to 0.19)	-0.10 (-0.23 to 0.03)	.06	-0.66 (-1.54 to 0.23)	-0.10 (-0.23 to 0.03)	.41	
Low baseline DASS-21 overall score	0.12 (-0.41 to 0.64)	0.03 (-0.10 to 0.15)	.33	0.10 (-0.42 to 0.63)	0.02 (-0.10 to 0.14)	.35	
DASS-21- stress subscale							.31
High baseline DASS-21 overall score	-0.73 (-1.84 to 0.39)	0.09 (-0.22 to 0.05)	.10	-0.75 (-1.88 to 0.39)	-0.09 (-0.22 to 0.05)	.12	
Low baseline DASS-21 overall score	0.31 (-0.17 to 0.54)	-0.04 (-0.15 to 0.07)	.24	-0.31 (-1.18 to 0.56)	-0.05 (-0.15 to 0.07)	.24	
Posttraumatic stress disorder (DTS)							.36
High baseline DASS-21 overall score	-0.44 (-5.03 to 4.15)	-0.01 (-0.12 to 0.10)	.43	-0.57 (-5.15 to 4.00)	-0.01 (-0.12 to 0.10)	.43	
Low baseline DASS-21 overall score	0.58 (-2.11 to 3.26)	0.02 (-0.08 to 0.12)	.34	0.63 (-2.04 to 3.30)	0.03 (-0.07 to 0.12)	.30	
Burnout (MBI-HSS) emotional exhaustion subscale							.26
High baseline DASS-21 overall score	-0.37 (-3.30 to 2.56)	-0.01 (-0.13 to 0.10)	.40	-0.23 (-3.13 to 2.67)	-0.03 (-0.13 to 0.10)	.34	
Low baseline DASS-21 overall score	0.94 (-1.12 to 3.05)	0.04 (-0.05 to 0.14)	.19	0.91 (-1.19 to 3.00)	0.04 (-0.05 to 0.14)	.19	
Burnout (MBI-HSS) professional accomplishment subscale [d]							.05
High baseline DASS-21 overall	0.43 (-1.20 to 2.06)	0.03 (-0.09 to 0.15)	.30	0.39 (-1.27 to	0.06 (-0.09 to 0.15)	.23	

score				2.05)			
Low baseline DASS-21 overall score	-1.44 (-2.55 to -0.25)	-0.10 (-0.20 to -0.01)	.02	-1.39 (-2.59 to - 0.20)	-0.12 (-0.22 to -0.02)	.01	
Burnout (MBI-HSS) depersonalization subscale							.25
High baseline DASS-21 overall score	0.46 (-0.45 to 0.67)	0.04 (-0.07 to 0.16)	.24	0.42 (-0.83 to 1.67)	0.04 (-0.07 to 0.15)	.23	
Low baseline DASS-21 overall score	-0.11 (-1.01 to 0.79)	-0.01 (-0.11 to 0.09)	.41	-0.11 (-1.01 to 0.79))	-0.01 (-0.11 to 0.09)	.40	
Insomnia (ISI)							.31
High baseline DASS-21 overall score	0.45 (-0.86 to 1.75)	0.03 (-0.65 to 0.13)	.25	0.40 (-0.90 to 1.70)	0.04 (-0.06 to 0.13)	.23	
Low baseline DASS-21 overall score	-0.03 (-0.99 to 0.94)	-0.01 (-0.10 to 0.09)	.48	-0.03 (-1.00 to 0.93)	-0.01 (-0.10 to 0.09)	.47	
Self-efficacy (GSE) [d]							.35
High baseline DASS-21 overall score	-0.62 (-1.74 to 0.51)	-0.06 (-0.18 to 0.05)	.14	-0.68 (-1.82 to 0.46)	-0.07 (-0.18 to 0.05)	.13	
Low baseline DASS-21 overall score	0.19 (-0.72 to 1.09)	0.02 (-0.08 to 0.12)	.34	0.20 (-0.69 to 1.10)	0.03 (-0.08 to 0.11)	.31	

Data are adjusted estimated (unstandardized) and standardized between-group mean differences, with 95% Confidence Intervals (CIs) in parentheses. P values not adjusted for multiple testing. DASS-21, Depression, Anxiety and Stress Scale; DTS, Davidson Trauma Scale; MBI-HSS, Maslach Burnout Inventory - Human Services Survey; ISI, Insomnia Severity Index; GSE, General Self-Efficacy Scale.

[a] Adjusted by baseline values
[b] Overall sample, derived by multiple imputation (50 imputations)
[c] Interaction *P* value for overall sample
[d] Scale scores were reversed to homogeneously convey that a negative difference favors the PsyCovidApp intervention

Table S5.2: Baseline use of psychotherapy (yes vs no)

	Sample of completers at follow-up (n=40)			Overall sample [b] (n=43)			Interaction [c]
	Estimated mean difference (95% CI) [a]	Standardized group difference (95% CI) [a]	*P* value	Estimated mean difference (95% CI) [a]	Standardized group difference (95% CI) [a]	*P* value	*P* value
DASS-21 overall score							.02
Psychotherapy users: Yes	-5.88 (-11.49 to -0.27)	-0.25 (-0.49 to -0.01)	.02	-5.90 (-11.55 to -0.24)	-0.25 (-0.49 to -0.02)	.02	
Psychotherapy users: No	-0.26 (-1.81 to 1.28)	-0.01 (-0.09 to 0.07)	.37	-0.26 (-1.82 to 1.30)	-0.01 (-0.09 to 0.07)	.39	
DASS-21- depression subscale							.06
Psychotherapy users: Yes	-1.29 (-3.46 to 0.88)	-1.39 (-0.37 to 0.10)	.13	-1.35 (-3.58 to 0.88)	-0.14 (-0.38 to 0.01)	.11	
Psychotherapy users: No	0.16 (-0.38 to 0.70)	0.02 (-0.06 to 0.10)	.28	0.19 (-0.35 to 0.74)	0.02 (-0.06 to 0.10)	.30	
DASS-21- anxiety subscale							.01
Psychotherapy users: Yes	-2.05 (-4.08 to -0.03)	-0.24 (-0.48 to 0.00)	.02	-2.00 (-4.04 to 0.04)	-0.24 (-0.48 to 0.00)	.03	
Psychotherapy users: No	-0.08 (-0.58 to 0.42)	-0.01 (-0.10 to 0.07)	.38	-0.07 (-0.58 to 0.44)	--0.01 (-0.10 to 0.07)	.37	
DASS-21- stress subscale							.05
Psychotherapy users: Yes	-2.51 (-4.92 to -0.10)	-0.28 (-0.55 to -0.01)	.02	-2.55 (-4.95 to -0.15)	-0.273 (-0.55 to 0.00)	.02	
Psychotherapy users: No	-0.37 (-1.09 to 0.36)	-0.04 (-0.12 to 0.04)	.16	-0.38 (-1.10 to 0.35)	-0.04 (-0.12 to 0.04)	.15	
Posttraumatic stress disorder (DTS)							.09
Psychotherapy users: Yes	-5.35 (-15.81 to 5.12)	-0.11 (-0.32 to 0.10)	.15	-5.27 (-15.76 to 5.22)	-0.10 (-0.31 to 0.12)	.16	
Psychotherapy users: No	0.70 (-1.94 to 3.28)	0.02 (-0.05 to 0.08)	.31	0.58 (-1.92 to 3.27)	0.02 (-0.05 to 0.08)	.30	
Burnout (MBI-HSS) emotional exhaustion subscale							.05
Psychotherapy users: Yes	-4.86 (-11.92 to 2.20)	-0.17 (-0.42 to 0.08)	.09	-5.04 (-12.10 to 2.02)	-0.17 (-0.42 to 0.08)	.08	
Psychotherapy users: No	0.84 (-0.97 to 2.65)	0.03 (-0.04 to 0.11)	.18	0.88 (-0.95 to 2.71)	0.04 (-0.04 to 0.11)	.17	
Burnout (MBI-HSS) professional accomplishment subscale [d]							.07
Psychotherapy users: Yes	-2.71 (-7.48 to 2.07)	-0.14 (-0.40 to 0.11)	.13	-2.59 (-7.29 to 2.12)	-0.15 (-0.41 to 0.10)	.14	
Psychotherapy users: No	-0.31 (-1.30 to 0.67)	-0.03 (-0.11 to 0.06)	.27	-0.27 (-1.23 to 0.70)	-0.02 (-0.10 to 0.06)	.29	

Burnout (MBI-HSS) depersonalization subscale							.19
Psychotherapy users: Yes	-0.76 (-3.12 to 1.59)	-0.06 (-0.26 to 0.13)	.26	-0.74 (-3.09 to 1.61)	-0.07 (-0.26 to 0.13)	.26	
Psychotherapy users: No	0.24 (-0.56 to 1.04)	0.02 (-0.06 to 0.10)	.27	0.26 (-0.54 to 1.05)	0.02 (-0.06 to 0.10)	.30	
Insomnia (ISI)							.03
Psychotherapy users: Yes	-2.29 (-4.74 to 0.16)	-1.99 (-0.41 to 0.01)	.03	-2.36 (-4.89 to 0.11)	-0.20 (-0.42 to 0.02)	.03	
Psychotherapy users: No	0.40 (-0.43 to 1.23)	0.03 (-0.03 to 0.10)	.17	0.37 (-0.45 to 1.19)	0.03 (-0.03 to 0.10)	.16	
Self-efficacy (GSE) [d]							.39
Psychotherapy users: Yes	-0.76 (-3.26 to 1.75)	-0.08 (-0.34 to 0.18)	.27	-0.77 (-3.31 to 1.78)	-0.08 (-0.34 to 0.18)	.27	
Psychotherapy users: No	-0.20 (-0.95 to 0.55)	- -0.02 (-0.10 to 0.06)	.30	-0.19 (-0.95 to 0.56)	-0.02 (-0.10 to 0.06)	.32	

Data are adjusted estimated (unstandardized) and standardized between-group mean differences, with 95% Confidence Intervals (CIs) in parentheses. P values not adjusted for multiple testing. DASS-21, Depression, Anxiety and Stress Scale; DTS, Davidson Trauma Scale; MBI-HSS, Maslach Burnout Inventory - Human Services Survey; ISI, Insomnia Severity Index; GSE, General Self-Efficacy Scale.

[a] Adjusted by baseline values
[b] Overall sample, derived by multiple imputation (50 imputations)
[c] Interaction *P* value for Overall sample
[d] Scale scores were reversed to homogeneously convey that a negative difference favors the PsyCovidApp intervention

Table S5.3: Baseline consumption of psychotropic medications (yes vs no)

	Sample of completers at follow-up (n=74)			Overall sample [b] (n=79)			Interaction [c]
	Estimated mean difference (95% CI) [a]	Standardized group difference (95% CI) [a]	*P* value	Estimated mean difference (95% CI) [a]	Standardized group difference (95% CI) [a]	*P* value	*P* value
DASS-21 overall score							.01
Psychotropic medication: Yes	-5.22 (-8.74 to -1.69)	-0.29 (-0.48 to -0.09)	<.01	-5.18 (-8.69 to -1.67)	-0.28 (-0.48 to -0.09)	<.01	
Psychotropic medication: No	0.10 (-1.54 to 1.74)	0.01 (-0.08 to 0.09)	.45	0.09 (-1.59 to 1.77)	0.01 (-0.08 to 0.09)	.45	
DASS-21- depression subscale							.06
Psychotropic medication: Yes	-1.07 (-2.61 to 0.46)	-0.14 (-0.35 to 0.06)	.08	-1.11 (-2.65 to 0.43)	-0.14 (-0.35 to 0.06)	.08	
Psychotropic medication: No	0.22 (-0.34 to 0.79)	0.03 (-0.05 to 0.12)	.50	0.24 (-0.33 to 0.81)	0.03 (-0.05 to 0.12)	.22	
DASS-21- anxiety subscale							<.001
Psychotropic medication: Yes	-1.94 (-3.34 to -0.55)	-0.26 (-0.45 to -0.08)	<.01	-1.94 (-3.34 to -0.54)	-0.26 (-0.45 to -0.07)	<.01	
Psychotropic medication: No	0.08 (-0.43 to 0.60)	0.01 (-0.08 to 0.10)	.37	0.09 (-0.42 to 0.60)	0.01 (-0.07 to 0.10)	.37	
DASS-21- stress subscale							.02
Psychotropic medication: Yes	-2.19 (-3.71 to -0.67)	-0.30 (-0.50 to -0.09)	<.01	-2.25 (-3.77 to -0.72)	-0.30 (-0.50 to -0.09)	<.01	
Psychotropic medication: No	-0.22 (-0.99 to 0.55)	-0.02 (-0.11 to 0.06)	.29	-0.19 (-0.95 to 0.56)	-0.02 (-0.11 to 0.06)	.29	
Posttraumatic stress disorder (DTS)							<.001
Psychotropic medication: Yes	-8.65 (-16.06 to -1.23)	-0.20 (-0.37 to -0.03)	.01	-8.80 (-16.26 to -1.33)	-0.20 (-0.37 to -0.03)	.01	
Psychotropic medication: No	1.90 (-0.78 to 4.58)	0.05 (-0.02 to 0.12)	.08	1.98 (-0.69 to 4.65)	0.05 (-0.02 to 0.12)	.09	
Burnout (MBI-HSS) emotional exhaustion subscale							.07
Psychotropic medication: Yes	-2.64 (-7.55 to 2.27)	-0.11 (-0.32 to 0.10)	.14	-2.78 (-7.71 to 2.16)	-0.12 (-0.32 to 0.09)	.13	
Psychotropic medication: No	0.91 (-0.94 to 2.76)	0.37 (-0.04 to 0.11)	.17	0.93 (-0.90 to 2.75)	0.37 (-0.04 to 0.11)	.16	
Burnout (MBI-HSS) professional accomplishment subscale [d]							.45
Psychotropic medication: Yes	-0.67 (-3.25 to 1.90)	-0.05 (-0.24 to 0.14)	.30	-0.61 (-3.20 to 1.98)	-0.05 (-0.24 to 0.14)	.30	
Psychotropic medication: No	-0.54 (-1.63 to 0.55)	-0.04 (-0.13 to 0.04	.17	-0.51 (-1.62 to 0.59)	-0.04 (-0.12 to 0.04)	.17	

Burnout (MBI-HSS) depersonalization subscale							.26
Psychotropic medication: Yes	-0.37 (-2.54 to 1.80)	-0.04 (-0.25 to 0.18)	.37	-0.38 (-2.56 to 1.79)	-0.04 (-0.25 to 0.17)	.36	
Psychotropic medication: No	0.29 (-0.51 to 1.08)	0.03 (-0.05 to 0.11)	.36	0.29 (-0.53 to 1.11)	0.03 (-0.05 to 0.10)	.25	
Insomnia (ISI)							.01
Psychotropic medication: Yes	-2.10 (-3.92 to -0.27)	-0.16 (-0.30 to -0.02)	.01	-2.08 (-3.92 to -0.24)	-0.16 (-0.30 to -0.02)	.01	
Psychotropic medication: No	0.53 (-0.29 to 1.39)	0.06 (-0.03 to 0.13)	.08	0.55 (-0.31 to 1.41)	0.05 (-0.02 to 0.12)	.09	
Self-efficacy (GSE) [d]							.49
Psychotropic medication: Yes	-0.11 (-1.89 to 1.66)	-0.01 (-0.21 to 0.19)	.45	-0.08 (-1.85 to 1.67)	-0.01 (-0.21 to 0.19)	.45	
Psychotropic medication: No	-0.23 (-1.01 to 0.54)	-0.02 (-0.11 to 0.06)	.27	-0.23 (-0.98 to 0.55)	-0.03 (-0.11 to 0.06)	.27	

Data are adjusted estimated (unstandardized) and standardized between-group mean differences, with 95% Confidence Intervals (CIs) in parentheses. P values not adjusted for multiple testing. DASS-21, Depression, Anxiety and Stress Scale; DTS, Davidson Trauma Scale; MBI-HSS, Maslach Burnout Inventory - Human Services Survey; ISI, Insomnia Severity Index; GSE, General Self-Efficacy Scale; SD, standard deviation.

[a] Adjusted by baseline values
[b] Overall sample, derived by multiple imputation (50 imputations)
[c] Interaction P value for overall sample
[d] Scale scores were reversed to homogeneously convey that a negative difference favors the PsyCovidApp intervention
