# Supplementary material for: A Mobile Phone–Based Intervention to Reduce Mental Health Problems in Health Care Workers During the COVID-19 Pandemic (PsyCovidApp): Randomized Controlled Trial
Source: JMIR Mhealth Uhealth. 2021 May 18;9(5):e27039. doi: 10.2196/27039 (PMC8133164; doi:10.2196/27039)
Supplement: Multimedia Appendix 4 [file mhealth_v9i5e27039_app4.pdf]

Multimedia Appendix 4. Number of health care workers recruited by Spanish region

	Number of health care workers (%)	Prevalence of COVID-19 cases at the onset of the recruitment period [a]
Illes Balears	114 (23.65%)	0.18% (2,090/1,149,460)
Cataluña	69 (14.32%)	0.54% (41,573/7,675,217)
Comunidad de Madrid	64 (13.28%)	0.97% (64,408/6,663,394)
Castilla y León	58 (12.03%)	0.97% (23,192/2,399,548)
Andalucía	46 (9.54%)	0.18% (15,466/8,414,240)
Aragón	39 (8.09%)	0.51% (6,767/1,319,291)
Comunitat Valenciana	34 (7.05%)	0.28% (13,779/5,003,769)
Castilla-La Mancha	15 (3.11%)	0.27% (20,477/7,675,217)
Galicia	15 (3.11%)	0.36% (9,838/2,699,499)
País Vasco	12 (2.49%)	0.61% (13,543/2,207,776)
Comunidad Foral de Navarra	5 (1.04%)	0.97% (6,340/654,214)
Extremadura	3 (0.62%)	0.46% (4,960/1,067,710)
Región de Murcia	3 (0.62%)	0.15% (2,307/1,493,898)
Ciudades autónomas de Ceuta y Melilla	2 (0.41%)	0.17% (299/171,264)
La Rioja	2 (0.41%)	1.24% (3,940/316,798)
Principado de Asturias	1 (0.21%)	0.26% (2,690/1,022,800)
Canarias	0 (0%)	0.11% (2,312/2,153,389)
Cantabria	0 (0%)	0.45% (2,630/581,078)
Total	482 (100%)	0.50% (236,611/47,026,208)

Healthcare workers were recruited between 14 May 2020 and 25 July 2020. Almost two thirds of the participants (63%) belonged to four of the 17 regions in Spain. The unequal distribution of health care workers across the Spanish regions indicates that the recruitment strategy did not homogenously reach all the regions.

[a] Computed based on the number of COVID-19 cases notified in Spain on 14 May 2020 (source: Informe sobre la situación de COVID-19 en España Informe COVID-19 nº 31. 14 de mayo de 2020. Available at: https://www.isciii.es/QueHacemos/Servicios/VigilanciaSaludPublicaRENAVE/EnfermedadesTransmisibles/Paginas/-COVID-19.-Informes-previos.aspx) and number of inhabitants (source: Spanish National Institute of Statistics (available at: https://www.ine.es/dyngs/INEbase/es/operacion.htm?c=Estadistica_C&cid=1254736177011&menu=resultados&secc=1254736195458&idp=1254734710990)
