# Supplementary material for: A Mobile Phone–Based Intervention to Reduce Mental Health Problems in Health Care Workers During the COVID-19 Pandemic (PsyCovidApp): Randomized Controlled Trial
Source: JMIR Mhealth Uhealth. 2021 May 18;9(5):e27039. doi: 10.2196/27039 (PMC8133164; doi:10.2196/27039)
Supplement: Multimedia Appendix 5 [file mhealth_v9i5e27039_app5.pdf]

Multimedia Appendix 5. Trial results reported in terms of adjusted mean between-group differences (overall sample)

Table S4.1: Comparison of outcome measures between the PsyCovidApp intervention and the Control App groups at 2 weeks in the overall sample

	Adjusted between-group mean differences (95% CI) [a]	
	Sample of completers at follow-up (n=436)	Overall sample [b] (n=482)
DASS-21 – overall score	-0.78 (-0.28 to 0.72)	-0.74 (-0.23 to 0.75)
DASS-21- depression	0.26 (-0.52 to 0.56)	0.26 (-0.52 to 0.57)
DASS-21- anxiety	-0.26 (-0.76 to 0.23)	-0.29 (-0.79 to 0.21)
DASS-21- stress	-0.57 (-1.26 to 0.13)	-0.58 (-1.27 to 0.12)
Posttraumatic stress disorder (DTS)	0.08 (-2.48 to 2.64)	0.04 (-2.53 to 2.60)
Burnout (MBI-HSS) emotional exhaustion	0.29 (-1.48 to 2.05)	0.26 (-1.51 to 2.02)
Burnout (MBI-HSS) professional accomplishment [c]	-0.57 (-1.57 to 0.43)	-0.55 (-1.55 to 0.45)
Burnout (MBI-HSS) depersonalization	0.14 (-0.62 to 0.90)	0.14 (-0.63 to 0.90)
Insomnia (ISI)	0.12 (-0.67 to 0.91)	0.14 (-0.66 to 0.94)
Self-efficacy (GSE) [c]	-0.23 (-0.94 to 0.48)	-0.24 (-0.96 to 0.47)

Data are adjusted between-group mean differences, with 95% Confidence Intervals (CIs) in parentheses. DASS-21, Depression, Anxiety and Stress Scale; DTS, Davidson Trauma Scale; MBI-HSS, Maslach Burnout Inventory - Human Services Survey; ISI, Insomnia Severity Index; GSE, General Self-Efficacy Scale.

[a] Adjusted by baseline values

[b] Overall sample, derived by multiple imputation (50 imputations)

[c] Scale scores were reversed to homogeneously convey that a negative difference favors the PsyCovidApp intervention
