# Supplementary material for: A Mobile Phone–Based Intervention to Reduce Mental Health Problems in Health Care Workers During the COVID-19 Pandemic (PsyCovidApp): Randomized Controlled Trial
Source: JMIR Mhealth Uhealth. 2021 May 18;9(5):e27039. doi: 10.2196/27039 (PMC8133164; doi:10.2196/27039)
Supplement: Multimedia Appendix 6 [file mhealth_v9i5e27039_app6.pdf]

# Multimedia Appendix 6. Credibility of Effect Modification Analyses (ICEMAN) instrument

## Credibility of Effect Modification Analyses (ICEMAN) instrument

**1: Was the direction of the effect modification correctly hypothesized a priori?**

| | | | |
|---|---|---|---|
| [ ] Definitely no | [ ] Probably no or unclear | [ ] Probably yes | [X] Definitely yes |
| *Clearly post-hoc or results inconsistent with hypothesized direction or biologically very implausible* | *Vague hypothesis or hypothesized direction unclear* | *No prior protocol available but unequivocal statement of a priori hypothesis with correct direction of effect modification* | *Prior protocol available and includes correct specification of direction of effect modification, e.g., based on a biologic rationale* |

Comment: *Described in the protocol available at https://www.medrxiv.org/content/10.1101/2020.11.03.20225102v1.full.pdf*

**2: Was the effect modification supported by prior evidence?**

| | | | |
|---|---|---|---|
| [ ] Inconsistent with prior evidence | [ ] Little or no support or unclear | [ ] Some support | [X] Strong support |
| *Prior evidence suggested a different direction of effect modification* | *No prior evidence or consistent with weak or very indirect prior evidence (e.g., animal study at high risk of bias) or unclear* | *Consistent with more limited or indirect prior evidence (e.g., large observational study, non-significant effect modification in prior RCT, or different population)* | *Consistent with strong prior evidence directly applicable to the clinical scenario (e.g., significant effect modification in related RCT)* |

Comment: *Results supported by two recent RCTs assessing the effectiveness of a digital health intervention to promote mental health in the general population:*

- Journal of affective disorders 2016; 203: 30-7.
- Journal of affective disorders 2019; 246: 695-705.

**3: Does a test for interaction suggest that chance is an unlikely explanation of the apparent effect modification?** (consider irrespective of number of effect modifiers)

| | | | |
|---|---|---|---|
| [ ] Chance a very likely explanation | [ ] Chance a likely explanation or unclear | [X] Chance may not explain | [ ] Chance an unlikely explanation |
| *Interaction p-value >0.05* | *Interaction p-value ≤0.05 and >0.01, or no test of interaction reported and not computable* | *Interaction p-value ≤0.01 and >0.005* | *Interaction p-value ≤0.005* |

Comment:

**4: Did the authors test only a small number of effect modifiers or consider the number in their statistical analysis?**

| | | | |
|---|---|---|---|
| [ ] Definitely no | [ ] Probably no or unclear | [ ] Probably yes | [X] Definitely yes |
| *Explicitly exploratory analysis or large number of effect modifiers tested (e.g., greater than 10) and multiplicity not considered in analysis* | *No mention of number or 4-10 effect modifiers tested and number not considered in analysis* | *No protocol available but unequivocal statement of 3 or fewer effect modifiers tested* | *Protocol available and 3 or fewer effect modifiers tested or number considered in analysis* |

Comment: Three effect modifiers tested

**5: If the effect modifier is a continuous variable, were arbitrary cut points avoided?** [X] not applicable: not continuous (for use of psychotherapy and of psychotropic medications)

| | | | |
|---|---|---|---|
| [ ] Definitely no | [ ] Probably no or unclear | [X] Probably yes | [ ] Definitely yes |
| *Analysis based on exploratory cut point (e.g., picking cut point associated with highest interaction p-value)* | *Analysis based on cut point(s) of unclear origin* | *Analysis based on pre-specified cut points, e.g., suggested by prior RCT* | *Analysis based on the full continuum, e.g., assuming a linear or logarithmic relationship* |

Comment: For the only continuous effect modifier variable the cut point was based on statistical criteria (median)

**6 Optional: Are there any additional considerations that may increase or decrease credibility?** (manual section 2.6)

[ ] Yes, probably decrease [X] Yes, probably increase

Comment: the effect modification is consistent across related outcomes

**7: How would you rate the overall credibility of the proposed effect modification?**

The overall rating should be driven by the items that decrease credibility. The following provides a sensible strategy:

- All responses definitely or probably reduced credibility or unclear → very low
- Two or more responses definitely reduced credibility → maximum usually low even if all other responses satisfy credibility criteria
- One response definitely reduced credibility → maximum usually moderate even if all other responses satisfy credibility criteria
- Two responses probably reduced credibility → maximum usually moderate even if all other responses satisfy credibility criteria
- No response options definitely or probably reduced credibility → high very likely

Place a mark on the continuous line (or type "x" in electronic version)

X

| **Very low credibility** | **Low credibility** | **Moderate credibility** | **High credibility** |
|---|---|---|---|
| Very likely no effect modification<br>Use overall effect for each subgroup | Likely no effect modification<br>Use overall effect for each subgroup but note remaining uncertainty | Likely effect modification<br>Use separate effects for each subgroup but note remaining uncertainty | Very likely effect modification<br>Use separate effects for each subgroup |
